# Supramolecular “baking powder”: a hexameric halogen-bonded phosphonium salt cage encapsulates and functionalises small-molecule carbonyl compounds†

**DOI:** 10.1039/d2sc04615f

**Published:** 2023-11-28

**Authors:** Joseph M. Marrett, Hatem M. Titi, Yong Teoh, Tomislav Friščić

**Affiliations:** a School of Chemistry, University of Birmingham Edgbaston Birmingham B15 2TT UK t.friscic@bham.ac.uk; b Department of Chemistry, McGill University 801 Sherbrooke St. W. Montreal H3A 0B8 Canada

## Abstract

We report a hexameric supramolecular cage assembled from the components of a Wittig-type phosphonium salt, held together by charge-assisted halogen bonds. The cage reliably encapsulates small polar molecules, including aldehydes and ketones, to provide host–guest systems where components are pre-formulated in a near-ideal stoichiometry for a mechanochemical base-activated Wittig olefination. These pre-formulated solids represent a proof-of-principle for a previously not reported supramolecular design of solid-state reactivity in which the host for molecular inclusion also acts as a complementary reagent for the subsequent chemical transformation of an array of guests. The host–guest solid-state complexes can act as supramolecular surrogates to their Wittig olefination vinylbromide products in a Sonogashira-type coupling that enables one-pot mechanochemical conversion of an aldehyde to an enediyne.

## Abstract 2

We report halogen-bonded supramolecular cages based on a Wittig-type phosphonium salt, which pre-formulate aldehydes and ketones for a mechanochemical Wittig olefination and enable the conversion of an aldehyde to an enediyne.

## Graphical abstract

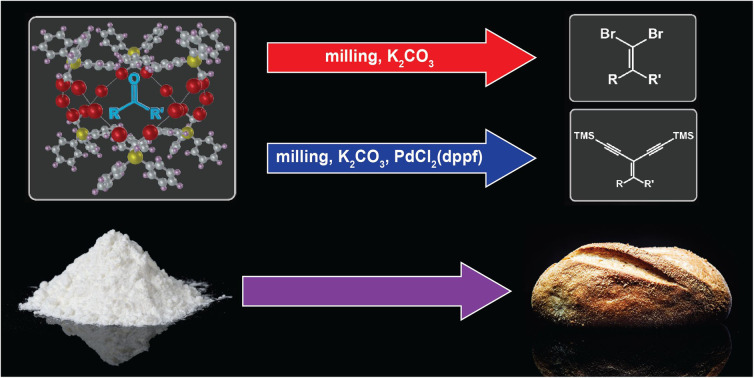

## Introduction

**Fig. 1:**
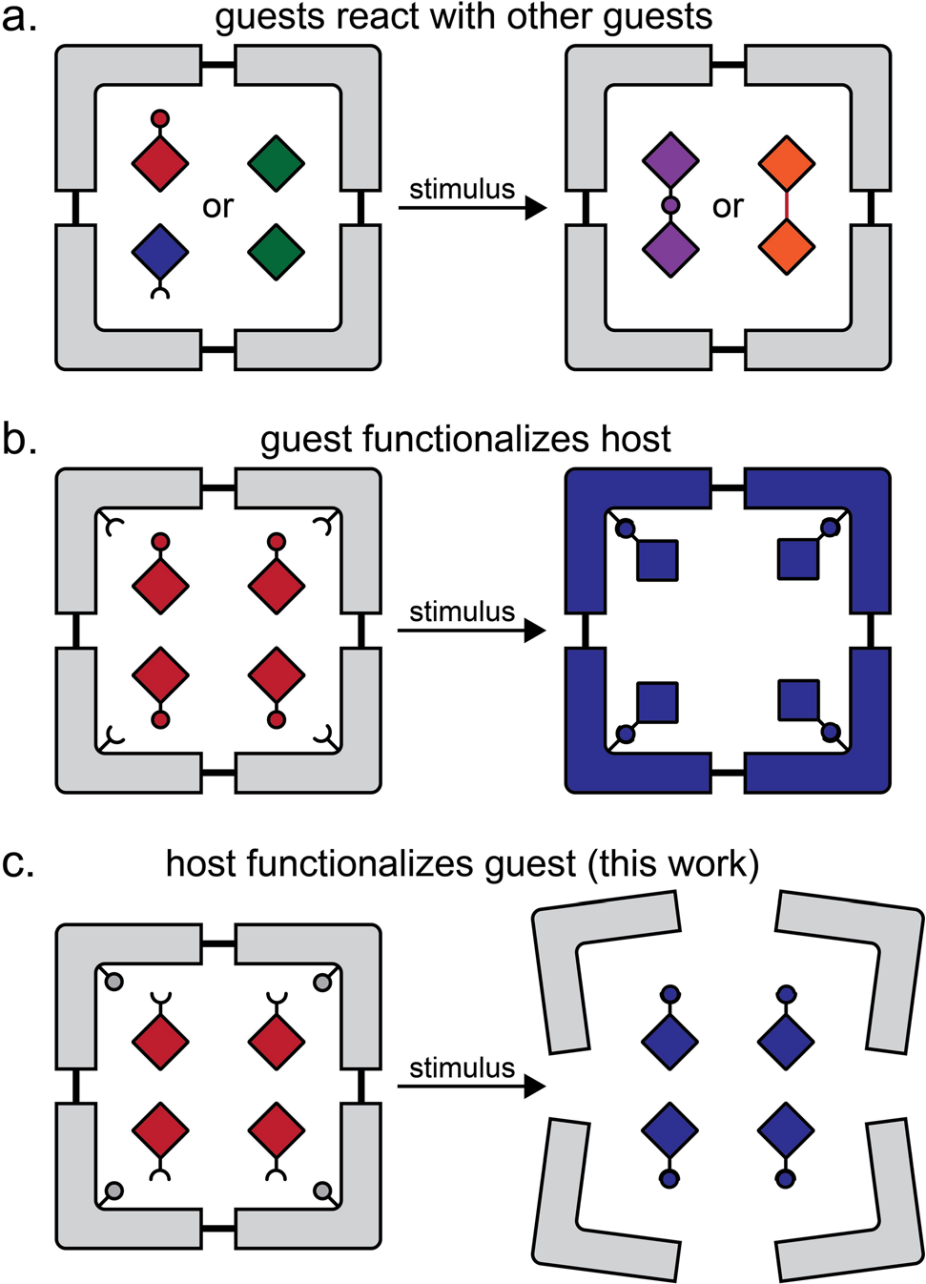
Comparison of the herein presented type of host–guest reactivity to other previously demonstrated ones: (a) reaction between similar or dissimilar guest molecules in a host structure;^16–18,26,27^ (b) a reaction in which the guest molecules functionalise the host structure, as exemplified by reactivity of ZIF-90 metal–organic framework with small molecule guests;^30^ and (c) the herein presented approach in which the host components functionalise the guest.

Formation of multi-component crystals, such as cocrystals^1–6^ lattice inclusion compounds^7–10^ and solid solutions,^11–14^ is one of the principal crystal engineering^15^ strategies for the design of reactivity in the organic solid-state. Molecular inclusion in host–guest complexes in the solid state, as well as in solution, has provided access to a range of transformations, including photodimerisations,^16–18^ isomerisations,^19–21^ photochemical oxidations,^22,23^ decarbonylations,^24,25^ Diels–Alder reactions,^26,27^ and more.^28^ In the majority of cases, the host molecule or lattice acts as a chemically inert container, with reactivity confined to the included guests (Fig. 1a).^29^ Recent work in the metal–organic framework (MOF) area has introduced the concept of a “crystal as a molecule”, wherein inclusion of a small molecule guest leads to the functionalisation of a suitably designed host framework (Fig. 1b).^30^

Here, we present a different and, to the best of our knowledge, not yet explored approach to design reactivity in organic solids, where the host component acts both as a container for inclusion and subsequently a reagent for chemical derivatisation of guests (Fig. 1c). We show the pre-formulation of two reactants into well-defined solid-state host–guest cages that can be used for “on demand” solvent-free Wittig olefination^31^ of small-molecule aldehydes and a ketone, induced by milling with a solid base. Moreover, we show that these inclusion compounds can be used to generate 1,1-dibromoolefins *in situ* for their further derivatisation, *i.e.* by a Sonogashira-type coupling.^32^ Effectively, these host–guest cage complexes represent a solid-state supramolecular equivalent of difficult-to-store dibromolefin liquids, enabling the one-pot conversion of an aldehyde into an eneyne and/or an enediyne, which proceeds readily by mechanochemistry, but with difficulty or not at all in solution.

## Results

**Fig. 2:**
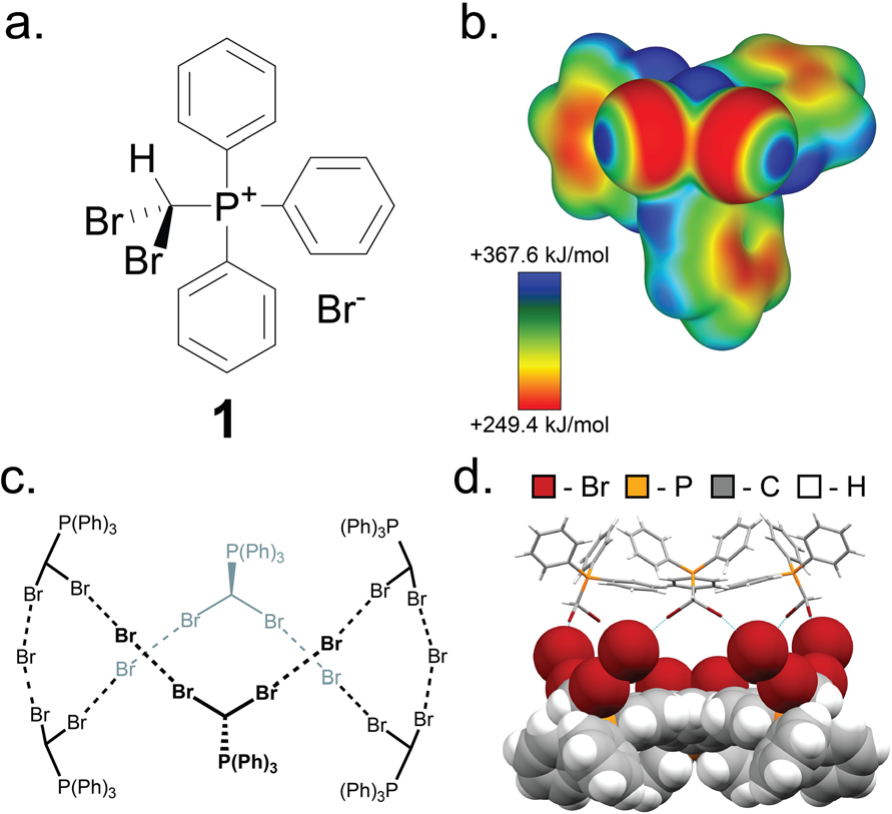
The XB cage based on 1. (a) Schematic representation of the salt (dibromomethyl)triphenylphosphonium bromide (1). (b) Electrostatic surface potential (ESP) map of the (dibromomethyl)triphenylphosphonium cation of 1, at an 0.0025 a.u. isosurface level. (c) Chemical diagram of the hexameric cage based on 1, and (d) fragment of the single crystal X-ray structure of 1·MeCN with the MeCN guest molecules omitted. One-half of the cage is displayed as capped sticks, and one-half in space-filling mode. Crystallographic data for all herein determined structures has been provided in the ESI† in CIF format, and also deposited with the Cambridge Structural Database (CCDC codes 2095086-2095103).

Key to this work is the herein shown ability of the (dibromomethyl)triphenylphosphonium bromide salt (PPh_3_CHBr_2_)^+^Br^−^ (1) to reliably form small molecule inclusion complexes in the solid state, based on a self-assembled hexameric cage held by charge-assisted halogen bonds (XBs, Fig. 2).^33–38^ Whereas phosphonium salts of the general type (PPh_3_CHX_2_)^+^X^−^ (X = Cl, Br, I) have been studied^39–41^ in the context of the Wittig olefination reaction as precursors to synthetically valuable *gem*-dihaloolefins,^42–46^ their structures and solid-state properties remain largely unexplored.

**Fig. 3:**
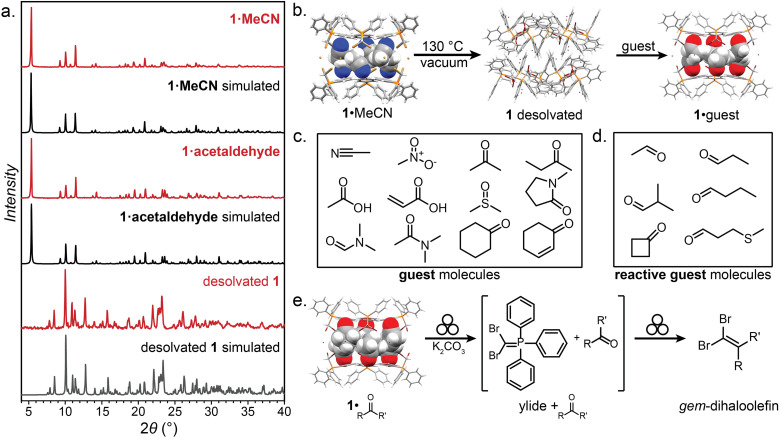
Selected PXRD patterns, guests, and transformations of 1. (a) The PXRD patterns of 1·MeCN and 1·acetaldehyde, which are similar, indicating isostructurality, and the pattern for desolvated 1, which is unique. (b) Schematic of the desolvation and solvation of 1·MeCN. (c) The guest molecules included in the hexameric XB cage of 1. (d) The reactive-guest molecules included in the hexameric XB cage of 1. (e) Reaction scheme for the mechanochemical Wittig olefination of the 1·reactive-guest materials induced by milling with a base (K_2_CO_3_). Crystallographic data for all herein determined structures has been provided in the ESI† in CIF format, and also deposited with the Cambridge Structural Database (CCDC codes 2095086-2095103). Symbol for mechanochemical reaction conditions in (e) was adopted from Rightmire and Hanusa.^53^

Compound 1 was synthesised according to a procedure by Wolkoff,^47^ and recrystallised from acetonitrile (MeCN) to yield large colorless crystals. Single crystal X-ray structural analysis revealed that the crystals are composed of hexameric cages, each containing six ordered MeCN molecules, held together by R–Br⋯Br^−^⋯Br–R halogen bonds as part of an apparently unique motif among so far reported halogen-bonded capsules, which are mostly dimeric.^48–52^ The resulting material (1·MeCN) was desolvated by heating (130 °C) under high vacuum for 12 hours, forming a new phase (1) with a distinct powder X-ray diffraction (PXRD) pattern (Fig. 3a and b). Dissolution of 1 in hot nitrobenzene, followed by slow cooling to room temperature, afforded colorless crystals, identified as the solvent-free salt (PPh_3_CHBr_2_)^+^Br^−^ by single crystal X-ray diffraction. The PXRD pattern simulated for the structure of 1 matched to that of the bulk material (Fig. 3a and b).

Compound 1 was found to consistently form the XB cage structure observed in 1·MeCN upon crystallisation from various liquids. Recrystallisation from a set of 11 additional small-molecule polar liquids (nitromethane (MeNO_2_), acetone, 2-butanone, acetic acid, acrylic acid, dimethylsulfoxide (DMSO), *N*-methylpyrrolidone (NMP), *N*,*N*-dimethylformamide (DMF), and dimethylacetamide (DMA), Fig. 3c) provided colorless crystalline 1·guest solids whose PXRD patterns were in all cases almost identical to that of 1·MeCN, indicating isostructurality (MeNO_2_), acetone, 2-butanone, acetic acid, acrylic acid, dimethylsulfoxide (DMSO), *N*-methylpyrrolidone (NMP), *N*,*N*-dimethylformamide (DMF), and dimethylacetamide (DMA)). Diffraction-quality single crystals were obtained for nine of these additional materials, confirming isostructurality and revealing the anticipated inclusion of solvent guest into the hexameric cage held together by R–Br⋯Br^−^⋯Br–R halogen bonds between 3.18 Å (for 1·MeNO_2_) and 3.45 Å (for 1·NMP) in length. In most structures, the guest molecules were sufficiently ordered for single crystal X-ray diffraction to reveal six of them located within each cage, with the electron-rich portions of each guest molecule engaging in short C–H⋯O (2.37–2.65 Å) or C–H⋯N (2.30 Å, for 1·MeCN) interactions with the phenyl groups of 1. In almost all cases, residual electron density which could not be modelled was also found at the center of each cage, indicating the presence of additional disordered guest.

The quantity of guest included in each hexameric cage was further investigated using proton nuclear magnetic resonance spectroscopy (^1^H NMR) in CDCl_3_ solution and by thermogravimetric analysis (TGA) (see ESI†). Analysis by ^1^H NMR revealed between 6 and 7 guest molecules per cage, commensurate with the X-ray single crystal structure analyses, and supporting the presence of additional highly disordered guests in some 1·guest materials. Similar results were obtained using TGA, where the amount of included guest was evaluated by the height of the first weight loss step observed upon heating each 1·guest material under a flow of N_2_. The number of guest molecules per cage for each 1·guest material, as determined by NMR and TGA, is given in the ESI.† All 1·guest materials were also analysed by Fourier-Transform Infrared Attenuated Total Reflectance spectroscopy (FTIR-ATR) (see ESI†).

The ready encapsulation of small polar molecules in 1 encouraged us to explore the possibility of encapsulating aldehydes and ketones, which could act as reactive complements to 1, which is an ylide precursor, in a Wittig olefination. We envisaged that encapsulation of carbonyl compounds (Fig. 3d and e) within the hexameric cage of 1 could lead to self-assembled 1·reactive-guest materials isostructural to 1·guest, but where the included guests would be susceptible to controlled chemical modification by the components of the host cage. Such an arrangement supposes that there is a sufficient barrier to the reaction between the host and guest, so that the solid-state complex can be isolated, stored and characterised without spontaneous reaction. In such a scenario, 1 represents a stable precursor to the phosphorus ylide which can react with carbonyl compounds to form olefins and is easily and reliably accessible by exposure to a base. This system can be seen as analogous to baking powder, a chemical leavening agent used in baking which is a pre-arranged mixture of two solids (a carbonate base and an acid) which are in close contact, but do not react until an external stimulus (*i.e.*, heat or liquid) is applied.^54^

A set of aldehydes and a cyclic ketone were selected as potentially reactive guests for this purpose (acetaldehyde, propionaldehyde, isobutyraldehyde, *n*-butyraldehyde, cyclobutanone, and 3-methylthiopropionylaldehyde (methional), Fig. 3d). The salt 1 could be recrystallised directly from cyclobutanone and methional by slow cooling, yielding colorless crystals isostructural to 1·MeCN, as confirmed by their PXRD patterns. Isostructurality was additionally confirmed for the cyclobutanone inclusion compound by single crystal X-ray diffraction (scXRD) structure analysis. The other reactive guest molecules dissolved 1 only slightly, and the 1·reactive-guest materials were obtained by soaking 1 in an excess of the pure liquid guest overnight. Soaking led to microcrystalline materials whose PXRD patterns indicated isostructurality to 1·MeCN. Single crystals of the 1·reactive-guest materials containing acetaldehyde and propionaldehyde were obtained by crystallisation from a mixture of the guest and a solvent which dissolves 1 but does not enter the cage (nitrobenzene and diethylpropionamide, respectively). For butyraldehyde and isobutyraldehyde as guests, placing a small amount of 1 in a large excess of the liquid guest overnight yielded single crystals suitable for scXRD structural analysis. Diffraction-quality single crystals could not be obtained for 1·methional and 1·butyraldehyde, but the PXRD patterns of these solids strongly indicate isostructurality to other XB cages.

Formation of 1·reactive-guest materials was also possible mechanochemically, by milling 1 with a guest compound. In a typical experiment, 100 mg of 1 and 100 μL of the guest were milled for between 5 and 30 minutes in a 15 mL volume zirconia milling jar, using a single zirconia ball of 3.2 g weight, leading to the formation of the 1·reactive-guest XB cages that were identified by PXRD after milling. Importantly, milling 1 on its own did not lead to the formation of a cage-type phase, instead causing amorphisation evident by the disappearance of well-defined reflections in the X-ray powder diffractogram. All six 1·reactive-guest materials were also analyzed by ^1^H NMR, TGA, and FTIR-ATR (see ESI†). The ^1^H NMR and TGA analyses indicated that the materials contained between four and seven guest molecules per each hexameric cage. Importantly, as each cage is expected to produce six equivalents of a phosphorus ylide upon milling with a base, the herein established compositions of 1·reactive-guest materials serendipitously correspond to a near-ideal stoichiometry for a Wittig olefination reaction. Solution ^1^H NMR spectra of all 1·reactive-guest materials showed the presence of only 1 and the guest molecular species, confirming that the host and guest are stable when in contact and that there was no reactivity between the host and reactive guest in the XB cage.

Next, we explored the potential of the 1·reactive-guest materials to undergo base-induced Wittig olefination by ball milling each material with K_2_CO_3_. Milling was performed at 30 Hz for 20–180 min, using a 15 mL volume zirconia milling jar containing a single 3.2 g weight zirconia ball. Immediate ^1^H NMR analysis of the milled materials showed a complete or nearly complete absence of 1 and the presence of triphenylphosphine oxide (TPPO), consistent with a Wittig olefination reaction.^55,56^ Similarly, PXRD analysis of the milled materials revealed the complete disappearance of Bragg reflections corresponding to the hexameric cage materials, and the appearance of reflections corresponding to TPPO and KBr (See ESI†). Moreover, the signals of the reactive guests in the ^1^H NMR spectra of milled materials were either significantly reduced or completely absent, replaced by those of the 1,1-dibromoalkenes expected from a Wittig olefination reaction using 1. The formation of the 1,1-dibromoalkenes was confirmed by comparing solution ^1^H and ^13^C NMR spectra to those for isolated pure compounds, which were also characterised by high-resolution mass spectrometry (HRMS) (see ESI†).

Conversions of 1·reactive-guest solids into 1,1-dibromoalkenes were determined by ^1^H NMR spectroscopic analysis of crude reaction mixtures after milling, using an internal standard approach in which the signal integration for the 1,1-dibromoalkene product was compared to that of the byproduct TPPO (see ESI†). Notably, ^1^H analysis of crude reaction mixtures showed in all cases the complete transformation of 1 into TPPO, which was also separately verified by ^31^P NMR spectroscopy in selected systems, indicating that the latter can be used as a suitable, non-volatile internal standard for analysis. Conversions were generally found to be >90% for aldehyde-based guests, but were lower for cyclobutanone (Table 1). In the case of isobutyraldehyde, the reaction led to a mixture of products which has so far been challenging to analyse. Notably, when conducted at the same scale (90 mg of 1·reactive-guest, which corresponds to 0.15–0.16 mmol, depending on the system), reactions of acetaldehyde and propionaldehyde guests gave conversions which were lower than those of butyraldehyde and methional. With the assumption that the lower conversions were due to the evaporative loss of the volatile guests or products into the milling jar headspace, the reactions were conducted at a larger scale (250 mg), and in the presence of a larger amount of base, in that way minimising the effect of evaporative loss on the measured conversion.‡‡Reactions of all substrates other than methional were found to improve upon increasing the amount of carbonate base beyond 1.1 equivalents. Since 1.1 equivalents of K_2_CO_3_ was found to be sufficient for complete conversion of 1 to TPPO for all substrates, we believe that improved conversions when using excess base may be due to reduced evaporation of reactants and/or products due to adsorption on the extra solid material.

**Table 1:** ^1^H NMR conversions for the mechanochemical Wittig reactions conducted by milling 1·reactive-guest with K_2_CO_3_ or Cs_2_CO_3_ as a base (also see ESI)

Product	Scale^a^ (mg)	Base (equivalents)	Time (min)	Conversion (%)
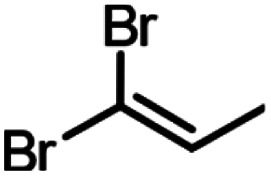	250	3	20	99, 94^b^
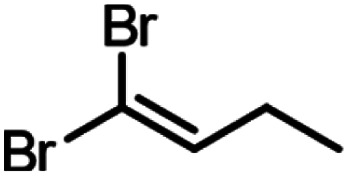	250	1.5	60	94, 99^b^
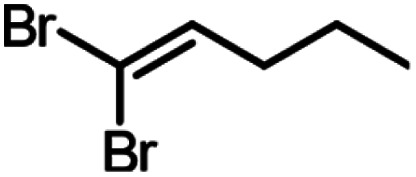	90	1.5	30	99, 98^b^
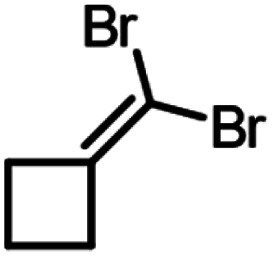	90	1.5	180	75, 77^b^
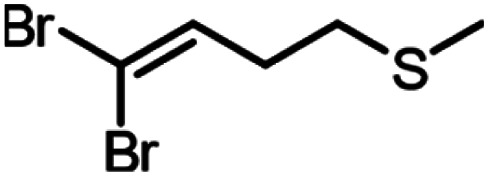	90	1.1	60	99, 92^b^

a Corresponds to the amount of 1·reactive guest.

b ^1^H NMR conversions for mechanochemical Wittig olefination where the K_2_CO_3_ is replaced with an equimolar quantity of Cs_2_CO_3_.

The effect of the base on reaction progress was evaluated by substituting K_2_CO_3_ with Cs_2_CO_3_ in the optimised milling experiments. The resulting conversions were similar to those seen when using K_2_CO_3_. We also attempted to investigate the importance of guest inclusion in the halogen-bonded cage of 1 by milling of non-solvated 1 with cyclobutanone in the presence of K_2_CO_3_. Cyclobutanone was chosen because the reaction of 1·cyclobutanone does not lead to complete conversion, in that way facilitating the observation of any reaction-enhancing or -retarding effects. This experiment revealed that the 1·reactive-guest complex formed rapidly, as shown by PXRD analysis of the reaction mixture after only 15 seconds of milling (see ESI†). While this prevents the exploration of the ball-milling reaction in the absence of guest encapsulation, it also provides a different perspective of 1·reactive-guest, at least in the case of cyclobutanone reactant: as a self-assembled intermediate in the Wittig reaction of 1 and reactive-guest. Such a perspective would be consistent with the emergent realisation that self-assembled solid-state complexes, such as cocrystals, can play an important role as intermediates in mechanochemical reactions.^57–59^

As another pathway to estimate how encapsulation in the cage of 1 could be affecting the reaction, we also explored milling of cyclobutanone with 1·MeCN in the presence of K_2_CO_3_ in equimolar quantities to the optimised reaction with 1·cyclobutanone. Pre-complexation of 1 with MeCN serves to limit the encapsulation of cyclobutanone, providing an experiment where the Wittig olefination can proceed without the influence of substrate capture. This experiment resulted in a conversion of 67%, a small, but noticeable decrease compared to the reaction of 1·cyclobutanone, for which the conversion was 75%. This result suggests a modest increase in reactivity is associated with substrate encapsulation by 1, with the caveat that additional MeCN could also be affecting the reaction.

**Fig. 4:**
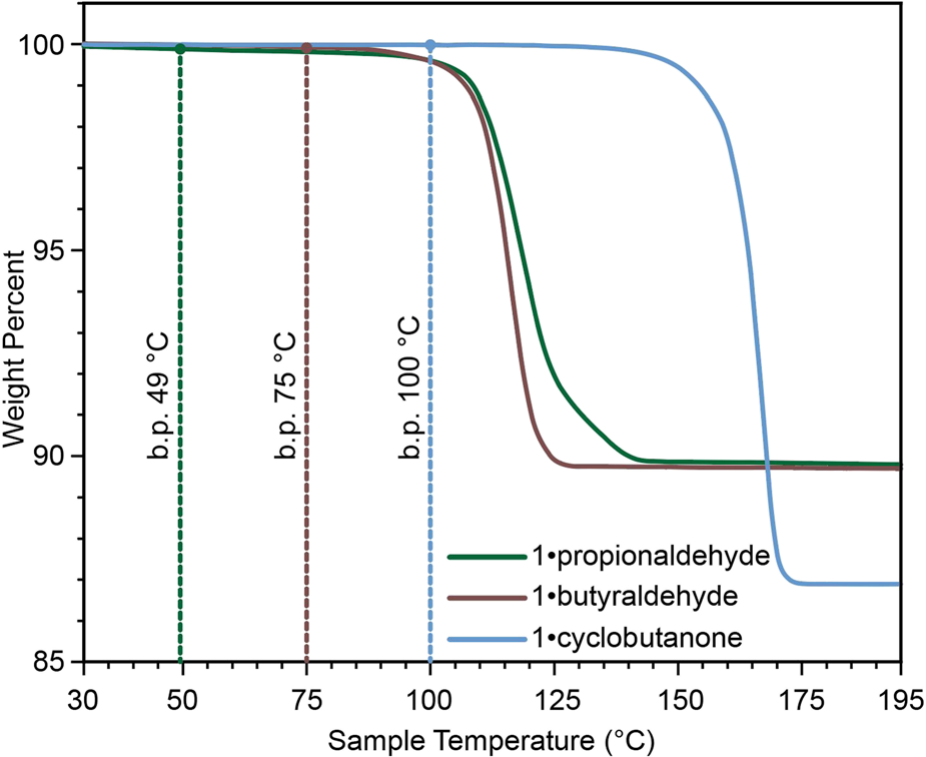
Comparison of TGA thermograms of 1·reactive-guest materials, with the boiling point of each reactive-guest marked with a dotted line, demonstrating that the 1·reactive-guest materials containing propionaldehyde, butyraldehyde, and cyclobutanone are stable at temperatures above the boiling points of the guests themselves.

The described 1·reactive-guest materials are unique examples of a supramolecular host encapsulating a molecular species which it is capable of derivatising upon chemical stimulus, such as base addition. For volatile and liquid carbonyl substrates like acetaldehyde, propionaldehyde, and cyclobutanone, this arrangement also provides the additional benefit of mitigating issues related to the storage and measurement of these liquids. Whereas small-molecule aldehydes and ketones are volatile, with boiling points between 20 °C and 100 °C, their associated 1·reactive-guest forms are stable to higher temperatures, as shown by TGA (Fig. 4).^10,60,61^ Additionally, the formation of 1·reactive-guest materials by crystallisation, soaking, or milling acts as a means by which to select an approximately stoichiometrically-appropriate quantity of substrate.

**Fig. 5:**
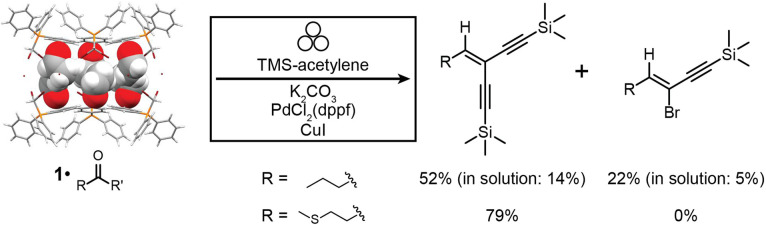
Reaction scheme for the one-pot mechanochemical Wittig olefination and Sonogashira coupling of aldehydes to form enediyne and eneyne products using 1·reactive-guest solids as starting materials. Conversions are given for mechanochemical reactions after 90 minutes milling, and for 1·butyraldehyde are compared to the best ones achieved for analogous reactions in solution after 24 hours. Symbol for mechanochemical reaction conditions was adopted from Rightmire and Hanusa.^53^

Based on the reliability and simplicity of the olefination reactions conducted by milling 1·reactive-guest with a base, we hypothesised that these materials could also act as supramolecular solid-state equivalents of difficult to store reactive liquid 1,1-dibromoalkenes in the context of further chemical derivatisation. Such a possibility would allow for the replacement of *gem*-dihaloolefins with stable precursor 1·reactive-guest solids based on aldehydes or ketones. To validate this possibility, we focused on the palladium- and copper-catalysed Sonogashira cross-coupling of alkynes with vinyl dibromides (Fig. 5), anticipated to yield eneyne or enediyne products.^62–64^ Molecular species containing the enediyne motif are of considerable synthetic interest as precursors in the synthesis of expanded radialenes, cross-conjugated macrocycles with interesting electronic properties.^65^

Milling 1·butyraldehyde with 2 equivalents of anhydrous K_2_CO_3_ and 2.2 equivalents of (trimethylsilyl)acetylene (TMS-acetylene) in the presence of 10 mol% of PdCl_2_(dppf)_2_ and 8 mol% of CuI for 30 minutes in a zirconia milling jar using a single zirconia ball (3.2 g weight) led to one-pot conversion of butyraldehyde to a mixture of mono- (eneyne) and di-coupled (enediyne) Sonogashira products, with a significant amount of the intermediate *gem*-dihaloolefin remaining. The stereochemistry of the eneyne product was confirmed by examination of *J*_CH_ couplings using the selHSQMBC-TOCSY experiment,^66^ which yielded values consistent with DFT calculations (see ESI†). Optimisation of the milling reaction by altering the milling time, quantity, solid form (anhydrous or sesquihydrate) of the K_2_CO_3_ base, and quantity of TMS-acetylene allowed for the near-complete disappearance of the *gem*-dihaloolefin intermediate and conversions of 52% for the enediyne and 22% for the eneyne Sonogashira products as determined by ^1^H NMR spectroscopy (Fig. 5 and Table 2). The enediyne product was isolated by column chromatography for the best performing reaction (reaction 5, Table 2), and gave an isolated yield of 45%, in good agreement with the 52% conversion determined by ^1^H NMR of the crude mixture. The sesquihydrate of K_2_CO_3_ generally performed better than the anhydrate as a base in these milling experiments. For the cage material containing 3-methylthiopropionaldehyde (1·methional), the enediyne product was obtained in 79% conversion, as determined by ^1^H NMR (see ESI†).

**Table 2:** Reaction conditions and conversions for mechanochemical one-pot Wittig olefination and Sonogashira coupling transformation of 1·butyraldehyde into the corresponding eneyne and enediyne. Conversions were determined by ^1^H NMR and, unless otherwise noted, K_2_CO_3_ was used in sesquihydrate form (see ESI). To illustrate the efficiency of the mechanochemical procedure, the outcomes two analogous solution reactions performed at 40 °C under argon have been included in this table. For comparisons with other analogous solution-based reactions, see ESI (Table S2.1)^a^

Entry	K_2_CO_3_ (equivalents)	TMS-acetylene (equivalents)	Reaction time (min)	1,1-Dibromoalkene	Eneyne conversion	Enediyne conversion
1	2^b^	2.2	30	17%	17%	17%
2	2	2.2	30	9%	17%	22%
3	2.5	2.2	30	9%	35%	39%
4	2.5	3.3	30	9%	43%	22%
5	2.5	3.3	90	Trace	22%	52% (45%)
6	2.5	3.3	180	Trace	4%	40%
7^c^	2.5	3.3	90	3%	0%	0%
8^c^	2.5	3.3	24 h	Trace	5%	14%

a All mechanochemical reactions demonstrated complete disappearance of starting materials by ^1^H NMR analysis, and the less-than-quantitative conversions to Sonogashira products are explained by the formation of poorly soluble side-products which are observed as a dark brown solid.

b Anhydrous K_2_CO_3_ was used.

c Reactions performed in solution at 40 °C, analogous to the mechanochemical reaction 5, and reaction 8 exhibits the highest yield herein achieved by solution methods.

Importantly, several attempts to perform this one-pot Wittig olefination and Sonogashira coupling from 1·butyraldehyde in solution, using either K_2_CO_3_ or diisopropylamine^44^ as a base, have produced zero or significantly lower conversions to eneyne or enediyne products compared to the optimised milling protocol (see ESI†). Moreover, Sonogashira coupling in solution starting from purified pre-synthesised 1,1-dihaloolefins using K_2_CO_3_ as a base produced only modest conversions to eneyne and enediyne products after 24 hours. In our hands, the only solution-based route to obtain the Sonogashira reaction coupling products in high yields was by first isolating the corresponding vinyl bromide reactant, followed by coupling in the presence of diisopropylamine base (see ESI†).^44^ Overall, the use of 1·butyraldehyde as a supramolecular surrogate of the corresponding vinyl bromide enabled the direct one-pot mechanochemical conversion of butyraldehyde into corresponding eneyne and enediyne derivatives without the need, apparently necessary in a solution-based protocols, to isolate the 1,1-dibromolefin and resort to an amine base.

**Fig. 6:**
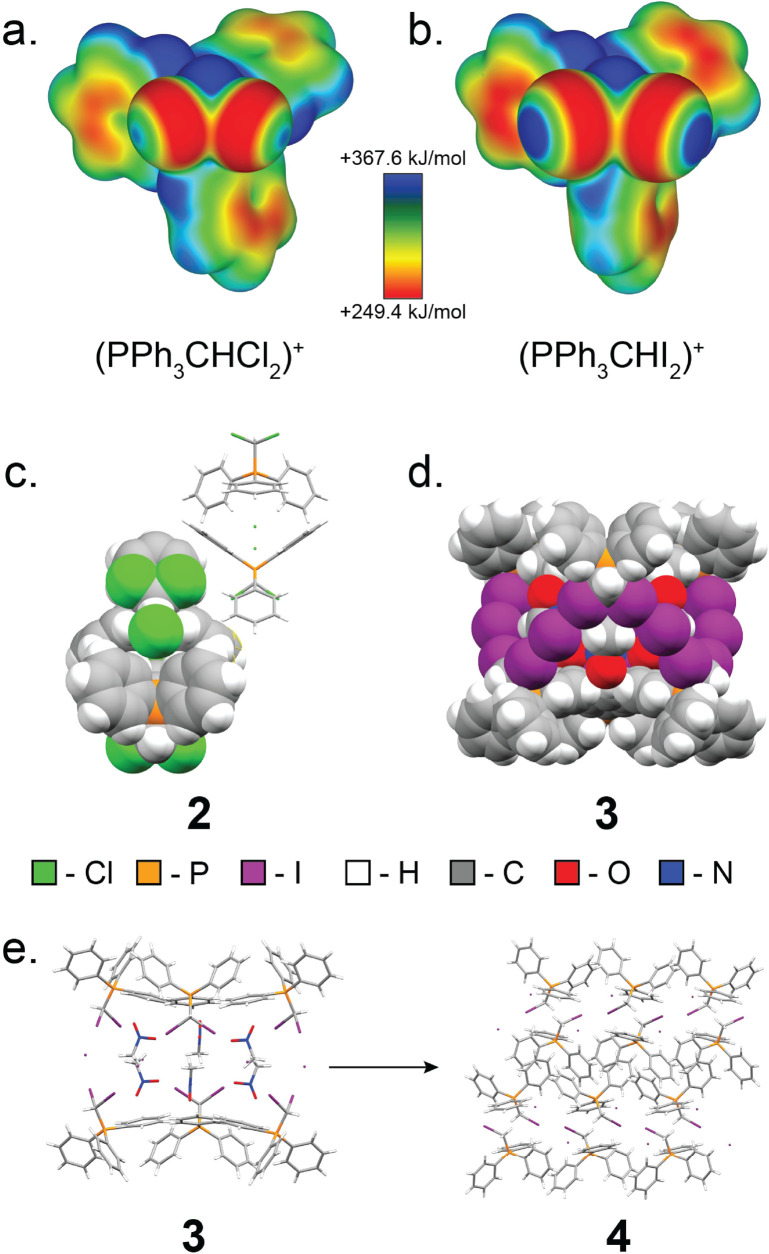
Overview of the structures and characteristics of 2 and 3. (a) Electrostatic surface potential (ESP) map of the (dichloromethyl)triphenylphosphonium cation, 2, plotted at an 0.0025 a.u. isosurface level. (b) ESP map of the (diiodo)triphenylphosphonium cation, 3, plotted at an 0.0025 a.u. isosurface level. (c) Fragment of the single crystal X-ray structure of 2, with one formula unit displayed in space-filling mode, and one formula unit displayed as capped sticks. (d) Fragment of the single crystal X-ray structure of 4, exhibiting the XB cage structure with nitromethane as the guest. (e) Reaction scheme for the decomposition of 3 MeNO_2_ to 4. The salt 3 converts to its monoiodinated analogue, 4, when dissolved in solvent and heated or left to crystallise in open air for extended periods of time.

Next, we explored expanding the 1·guest cage motif to chloro- and iodo-congeners of 1. To this end, (dichloromethyl)triphenylphosphonium chloride (2, Fig. 6) was synthesised following a modified procedure reported by Appel,^67^ and was recrystallised from MeCN to yield single crystals suitable for scXRD analysis. Instead of forming a halogen-bonded cage analogous to 1·MeCN, compound 2 adopts a dense-packed non-solvated structure exhibiting C–H⋯Cl^−^ hydrogen bonding interactions. All attempts to obtain a cage based on 2 have been unsuccessful, consistent with weaker capacity of chlorine as XB donor.

Iodine is known to engage in XB interactions with greater propensity than bromine or chlorine,^33–35,68^ suggesting a way to obtain materials analogous to 1·guest. Heating to reflux a solution of iodoform with one equivalent of triphenylphosphine in MeCN gave mostly (diiodomethyl)triphenylphosphonium iodide, 3, as a yellow solid with a PXRD pattern suggesting a structure similar to that of 1·MeCN. Dissolution of this powder in nitromethane followed by rapid crystallisation under reduced pressure yielded crystals suitable for scXRD analysis, which revealed XB cages isostructural to those in 1·MeCN (Fig. 6). However, 3 was found to readily decompose, forming (iodomethyl)triphenylphosphonium iodide (4), which was identified by scXRD analysis of crystals grown by heating 3 in MeNO_2_ until boiling, followed by slow cooling of the solution (Fig. 6).

## Conclusions

We have presented a proof-of-principle of a supramolecular halogen-bonded host that encapsulates molecular species which are susceptible to subsequent chemical transformation by the host itself. This strategy allows for the creation of thermally stable complexes of guest aldehydes or ketones with a phosphonium salt host, in a stoichiometric ratio that is very close to that required for the Wittig olefination reaction. Consequently, these host–guest complexes can be used as solid-state supramolecular surrogates of reactive liquid vinyl dibromides, that are readily and in high conversions generated by mechanochemical treatment of the solid-state complexes with a carbonate base. This approach also enables more complex one-pot transformations by mechanochemistry, illustrated herein by the tandem Wittig olefination/Sonogashira coupling,^55,56,62–64^ which readily took place mechanochemically, but much less efficiently or not at all when attempted in solution. These results, while currently limited in the choice of reagents, serve as a proof-of-principle to illustrate how the use of a reactive host–guest complex as a supramolecular surrogate of a vinyl bromide in the solid state not only simplifies the derivatisation of volatile aldehydes and a ketone, but also enables otherwise inefficient, more complex one-pot transformations. Work is ongoing to expand this methodology to additional carbonyl-containing substrates, and to explore other reaction systems which could benefit from using host–guest assemblies as supramolecular surrogates.

## Data availability

Crystallographic data in CIF format has been deposited with the Cambridge Structural Database. Data supporting this manuscript has been provided in the ESI.†

## Author contributions

J. M. M. performed synthesis, characterisation, and calculations. H. M. T. performed scXRD analysis. T. Y. assisted with mechanochemical synthesis optimisation. The manuscript was written by J. M. M. and T. F. with contributions from all authors.

## Conflicts of interest

There are no conflicts to declare.

## Supplementary Material

SC-015-D2SC04615F-s001

SC-015-D2SC04615F-s002
