# Supplementary material for: Supramolecular “baking powder”: a hexameric halogen-bonded phosphonium salt cage encapsulates and functionalises small-molecule carbonyl compounds
Source: Chem Sci. 2023 Nov 28;15(1):298–306. doi: 10.1039/d2sc04615f (PMC10732138; doi:10.1039/d2sc04615f)
Supplement: SC-015-D2SC04615F-s001 [file SC-015-D2SC04615F-s001.pdf]

Electronic Supplementary Material (ESI) for Chemical Science.
This journal is © The Royal Society of Chemistry 2023

Supplementary Information

Wittig olefination "baking powder": a hexameric halogen-bonded phosphonium salt cage for encapsulation and solid-state transformation of small-molecule aldehydes and ketones

Joseph M. Marrett,[a,b] Hatem M. Titi,[b] Yong Teoh,[b] and Tomislav Friščić[a,b]*

a) School of Chemistry, University of Birmingham, Edgbaston, Birmingham B15 2TT, United Kingdom; b) Department of Chemistry, McGill University, 801 Sherbrooke St. West, Montreal, Quebec H3A 0B8, Canada

Table of Contents

S1. Materials 2
S2. Synthetic methods 2
S3. Computational methods 7
S4. Instrumental methods 7
S5. Powder X-ray diffraction data 11
S6. Infrared spectroscopy 19
S7. Thermogravimetric analysis 21
S8. Number of guest molecules per cage as determined by TGA and NMR 23
S9. NMR determination of stereochemistry of eneyne product 24
S10. Analysis of organic products 25
S11. ^{1}H NMR quantitative methods 27
S12. ^{1}H NMR spectra of cage materials 28
S13. ^{1}H NMR spectra of crude Wittig olefination reaction milling products 38
S14. Raw ^{1}H and ^{13}C NMR spectra of purified products 43
S15. References 51

S1. Materials

Carbon tetrabromide (99%), acetaldehyde (≥99%), acrylic acid (99%), dimethylacetamide (DMA) (≥99%), nitromethane ($MeNO_2$) (≥95%), N-methyl-2-pyrrolidone (NMP) (99%), nitrobenzene (99%), cyclohexanone (99.8%), iodoform (99%), benzene (anhydrous, 99.8%), carbon tetrachloride (99.9%), cesium carbonate (99%), and copper(I) iodide (≥99.5%) were obtained from Sigma Aldrich. Triphenylphosphine (99%), cyclohex-2-enone (98%), propionaldehyde (97%), isobutyraldehyde (99%), butyraldehyde (96%), 3-(methylthio)propionaldehyde (methional) (97%), cyclobutanone (≥98%), diethyl propionamide (≥95%), (trimethylsilyl)acetylene (98%) and 1,1'-bis(diphenylphosphino)ferrocene-palladium(II) dichloride dichloromethane complex (98%) were obtained from Oakwood Chemical. Dimethyl sulfoxide (DMSO) (99.9%), dimethyl formamide (DMF) (99.8%), acetic acid (99.5%), and potassium carbonate (99%) were obtained from ACP Chemicals. Butanone (99.9%), and acetone (99.5%) were obtained from Fischer chemical. Methional was distilled under high vacuum before use. Benzene was stored over molecular sieves and subject to three cycles of freeze-pump-thaw degassing under argon before use in solution experiments. All other chemicals were used without purification.

S2. Synthetic methods

S2.1. Synthesis of (dibromomethyl)triphenylphosphonium bromide (1)

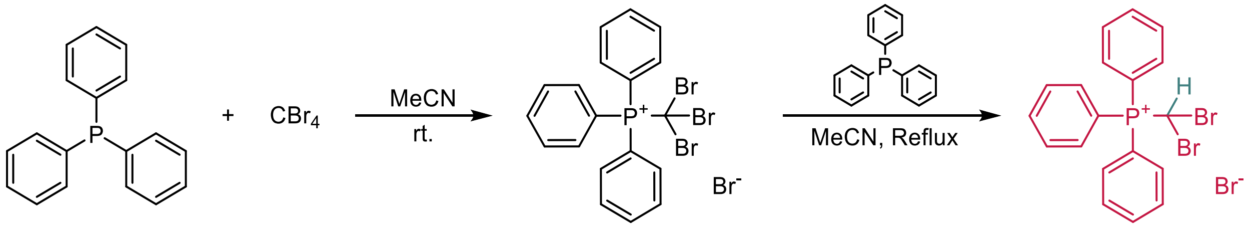

Scheme 1. Reaction scheme for the synthesis of **1**.

Compound **1** was synthesised according to a 2-step procedure previously reported by Wolkoff.[1] The general scheme for this reaction is shown above. The product of this synthesis is microcrystalline **1•MeCN** which can be recrystallized carefully from MeCN to yield crystals suitable for single-crystal X-ray diffraction analysis (scXRD). Yield for step 1: 84 %. Yield for step 2: 44%.

S2.2. Desolvation of 1•MeCN to form desolvated 1

Microcrystalline **1•MeCN** was exposed to 130 °C under high vacuum for 14 hours to yield desolvated **1**. Recrystallisation of this powder by slow cooling from nitrobenzene produces single crystals suitable for scXRD analysis, which revealed the structure of solvent-free **1**.

S2.3. Procedure for synthesis of 1•guest materials

Materials of the type **1•guest** were formed by recrystallisation of **1** from hot liquid **guest** followed by collection by filtration. In the case of materials grown from non-volatile liquids (NMP, DMF, DMA, acetic acid, acrylic acid, DMSO, cyclohexanone, cyclohex-2-enone), the solid product is rinsed with benzene during the filtration step before drying on the frit for 5 minutes. In the case of cyclohexanone and cyclohex-2-eneone, precipitation was carried out in the freezer (-18 °C). In cases where single crystal X-ray diffraction structures were collected, suitable crystals were collected from the mother liquor by pipette before filtration.

S2.4. Procedure for the synthesis of 1•reactive-guest materials

Bulk powders of the **1•reactive-guest** materials used in all milling experiments were obtained by soaking 200 mg (0.39 mmol) of **1** in 1 mL of the liquid guest for 24 hours followed by filtration and washing with benzene. Single crystals of **1•acetaldehyde** were grown by recrystallisation of **1** from a hot mixture of 50 v/v% acetaldehyde in nitrobenzene. Single crystals of **1•propionaldehyde** are grown in the same fashion from a 50 v/v% mixture of propionaldehyde in N,N-diethylpropionamide. Single crystals of **1•butyraldehyde** and **1•isobutyraldehyde** were obtained by adding a very small amount (~5 mg) of **1** to a large excess (1 mL) of liquid aldehyde and letting the powder soak for 24 hours to yield single crystals. X-ray quality single crystals **1•cyclobutanone** could be obtained by simple recrystallisation of **1** from the hot liquid cyclobutanone.

S2.5. Procedure for the synthesis of reactive 1•guest materials by milling

Bulk powders of the **1•reactive-guest** materials can also be obtained by ball-milling **1** with the guest liquid. 100 mg (0.19 mmol) of **1** and 100 µL of the liquid guest are added to a 15 mL zirconia milling jar with a single zirconia ball of 3.2 g weight. The mixture is milled for 5 to 30 minutes (see PXRD data for specific times) at 30 Hz using a Form-Tech Scientific FTS-1000 shaker mill.

S2.6. General procedure for the mechanochemical Wittig olefination *via* 1•guest

Powders of **1•reactive-guest** and K_2CO_3 (between 1.1 and 3.0 equivalents, as calculated based on the guest loadings listed in section S8) are added to a 15 mL zirconia milling jar with a single 3.2 g zirconia ball, the jar is sealed tightly with electrical tape, and the mixture is milled at 30 Hz using a Form-Tech Scientific FTS-1000 shaker mill for between 20 and 180 minutes. Analysis was done immediately by ^{1}H NMR spectroscopy the entire product mixture dissolved in $CDCl_3$ and filtered over a cotton and celite plug to remove any undissolved solids (i.e. KBr). Conversions were determined by ^{1}H NMR as explained in S11.2. The *gem*-dibromoolefin product was purified either by distillation from the solid reaction mixture or column chromatography; specifics for each sample are listed in section S10.

Control reactions for the mechanochemical Wittig olefination reactions using Cs_2CO_3 were conducted with identical parameters to the optimized reactions for each substrate. The results of these control reactions are listed below in Table S2.1

Table S2.1. Conditions and conversions of mechanochemical Wittig olefination reactions using Cs_2CO_3 as a base.

Product	Scale	Cs_2CO_3 (equivalents)	Time	Conversion
1,1-dibromopropene	250 mg	3	20 min	94%
1,1-dibromobutene	250 mg	1.5	60 min	99%
1,1-dibromopentene	90 mg	1.5	30 min	98%
(dibromomethylene)cyclobutane	90 mg	1.5	180 min	77%
1,1-dibromo-4-methylthiobutene	90 mg	1.1	60 min	92%

S2.6. Control experiments to investigate the effect of substrate encapsulation on mechanochemical Wittig olefination reactions

Experiments to determine the effect of substrate encapsulation on the course of the mechanochemical Wittig olefination were first attempted by milling, at 30 Hz, desolvated **1** with K_2CO_3 and cyclobutanone in molar quantities equal to those of the optimized reaction based on the pre-complexed material. However, PXRD analysis (see Figure S6.5) of the milled mixture after 15 seconds showed the formation of **1•reactive-guest**, revealing that the experiment is invalid to control for encapsulation. To reduce the possibility of complexation of the reactive guest by **1**, the experiment was repeated on the cyclobutanone with an equimolar quantity of **1•MeCN** instead of **1**. Conversions were determined using the approach detailed in S11.2. The results of both of these experiments are tabulated below in Table S2.2.

Table S2.2. Conditions and conversions of mechanochemical Wittig olefination reactions using **1•MeCN** or **1** with pure reactive guest

Source of **1**	Cyclobutanone	K_2CO_3 (equivalents)	Time	Conversion
87 mg **1•MeCN**	10.8 mg	1.5	180 min	67%
79 mg **1**	10.8 mg	1.5	180 min	66%

S2.7. Procedure for the one-pot mechanochemical combination of Wittig olefination and Sonogashira coupling using 1•guest as the starting material

In a typical experiment, 90 mg of **1•reactive**-**guest** material is added to a 15 mL zirconia milling jar with one 3.2 g zirconia ball. 10 mol % $PdCl_2(dppf)\cdot CH_2Cl_2$, 8 mol % CuI, potassium carbonate, and (trimethylsilyl)acetylene are added and the mixture is milled at 30 Hz using a Form-Tech Scientific FTS-1000 shaker mill. The required mass of catalyst was calculated based on the quantity of guests, assuming a loading of 5 molecules per cage. The temperature of the reaction mixtures immediately after milling was measured using an infrared thermometer and was found to never exceed 29 °C. Analysis proceeded immediately by ^{1}H NMR spectroscopy of a small amount of product mixture dissolved in $CDCl_3$ and filtered over a cotton plug to remove any undissolved solids. Conversions were determined by ^{1}H NMR as explained in S11.2. Purification of the product(s) proceeds by column chromatography of the jar contents; specifics for each product are listed in section S10.

S2.8. Procedure for attempted one-pot combination of Wittig olefination and Sonogashira coupling in solution

For each experiment, 90 mg of **1•butyraldehyde**, 2 mL of anhydrous benzene degassed by three freeze-pump-thaw cycles under argon, a base, $PdCl_2(dppf)\cdot CH_2Cl_2$ (10 mol %), CuI (8 mol %), and (trimethylsilyl)acetylene (3.3 eq.) were added to a sealed, oven-dried vial and stirred under argon for the time indicated in Table S2.3. Reactions using diisopropylamine mimic conditions used by Uenishi *et al.* for the coupling of terminal acetylenes with 1,1-dibromoolefins.[2] Workup for all reactions involved the removal of solvent *in vacuo* before ^{1}H NMR analysis of the crude product mixtures. Conversions were determined by ^{1}H NMR as explained in S11.2.

Table S2.3. Conditions and conversions for attempted one-pot combination of Wittig olefination and Sonogashira coupling in solution.

Base	Reaction time	Reaction temperature	Wittig product	Sonogashira products
2.5 eq. K_2CO_3	90 min	room temperature	3%	none
2.5 eq. K_2CO_3 sesquihydrate	90 min	room temperature	3%	none
2.5 eq. K_2CO_3	24 hours	room temperature	23%	none
2.5 eq. K_2CO_3 sesquihydrate	24 hours	room temperature	none	eneyne: 17%<br>enediyne: 8%
2.5 eq. K_2CO_3	90 min	40 °C	5%	none
2.5 eq. K_2CO_3 sesquihydrate	90 min	40 °C	3%	none
2.5 eq. K_2CO_3	24 hours	40 °C	14%	none
2.5 eq. K_2CO_3 sesquihydrate	24 hours	40 °C	none	eneyne: 5%<br>enediyne: 14%
4 eq. *i*-Pr_2NH See Ref.[2]	90 min	room temperature	25%	none
4 eq. *i*-Pr_2NH See Ref.[2]	90 min	40 °C	41%	none
4 eq. *i*-Pr_2NH See Ref.[2]	24 hours	room temperature	30%	eneyne: 9%<br>enediyne: none
4 eq. *i*-Pr_2NH See Ref.[2]	24 hours	40 °C	22%	eneyne: 7%<br>enediyne: none

S2.9. Procedure for attempted solution-based Sonogashira coupling from pre-synthesised 1,1-dibromopentene

To an oven-dried vial were added 30 mg (0.13 mmol) 1,1-dibromopentene, $PdCl_2(dppf)\cdot CH_2Cl_2$ (10 mol %), CuI (8 mol %), (trimethylsilyl)acetylene (3.3 eq), and 2 mL of degassed anhydrous benzene. As a base were used either 1.5 equivalents K_2CO_3 (anhydrous or sesquihydrate) or three equivalents of diisopropylamine.[2] The reaction vial was sealed, and the reaction mixture was stirred under argon for the time indicated in Table S2.4. Conversions were determined by comparing ^{1}H NMR integration of signals of the dibromide starting material and the signals of the eneyne and enediyne products.

Table S2.4. Conditions and conversions for solution-based Sonogashira coupling reactions starting from 1,1-dibromopentene.

Base	Reaction time	Reaction temperature	Remaining starting material (1,1-dibromopentene)	eneyne conversion	enediyne conversion
1.5 eq. K_2CO_3	90 min	room temperature	100%	none	none
1.5 eq. K_2CO_3 sesquihydrate	24 hours	room temperature	50%	39%	11%
3 eq. *i*-Pr_2NH See Ref.[2]	90 min	room temperature	none	79%	21%
3 eq. *i*-Pr_2NH See Ref.[2]	24 hours	room temperature	none	none	100%

S2.10. Procedure for the synthesis of (dichloromethyl)triphenylphosphonium chloride (2)

The salt **2** was synthesised according to a modified procedure reported by Appel *et al*,[3] scaled down 100-fold. 2 mL (20.7 mmol) of CCl_4 and 524 mg (2 mmol) of triphenylphosphine were added to a mixture of 4.2 mL benzene and 0.8 mL acetonitrile. The mixture was heated to 50 °C in a sealed vial with rapid stirring. After 5 minutes, the mixture turned a pale yellow, and the vial was opened to the air with continued stirring. After 10 minutes, a white precipitate formed which was isolated by filtration and washed with a minimal amount of benzene. Crystals for scXRD structural analysis were obtained by slow evaporation of a solution of this material in MeCN. Yield: 55%.

S2.11. Procedure for the synthesis of (diiodomethyl)triphenylphosphonium iodide (3) and (iodomethyl)triphenylphosphonium iodide (4)

1.18 g (3 mmol) of iodoform and 787 mg (3 mmol) of triphenylphosphine were added to 5 ml of MeCN and dissolved fully by sonication. The mixture was refluxed for 5 minutes, until a deep red paste formed on the walls of the vessel. This paste was broken up using a glass rod and by submersion of the sealed vessel in a sonicating bath, converting it to a yellow powder in the process. This powder was collected by filtration then was suspended in 10 mL of acetonitrile and again collected by filtration before drying briefly on the frit. After collection, the product was stored under an inert atmosphere. Single crystals of **3•MeNO$_2$** were grown by evaporation of a solution, made by dissolving this powder in $MeNO_2$, under high vacuum over the course of approximately 20 minutes. Longer evaporation times, or the use of elevated temperature produced single crystals of

(iodomethyl)triphenylphosphonium iodide (**4**) whose crystal structure is also reported here.

S3. Computational methods

S3.1 Electrostatic surface potential map (ESP) calculations

Electrostatic surface potential maps were calculated for the cations of salts **1**, **2**, and **3**, based on their structures after geometry optimisation using Gaussian 16[4] at the B3LYP/cc-pVTZ level of theory.[5] The basis set aug-cc-pVTZ was used for the iodine atoms of **3**, and was sourced from the basis set exchange.[6] ESPs were visualized in Vesta.[7]

S3.2. Nuclear magnetic resonance (NMR) calculations

Nuclear magnetic resonance spectra (chemical shifts and *J*-couplings) were simulated for both possible isomers (E and Z) of the eneyne produced from **1·butyraldehyde**. Geometry optimization was performed using Gaussian 16[4] at the B3LYP/6-31+G(d,p) level of theory. Single-point NMR calculations were then performed at the MPW1PW91/6-311+G(2d,p) level of theory, in chloroform as a solvent, using the Gauge-Independant Atomic Orbital (GIAO) method[8] in Gaussian 16. *J*-coupling constants were calculated using the *mixed* keyword.[9] These calculations yielded *J*-couplings which were compared to those determined by NMR, as described in S9.

S4. Instrumental methods

S4.1. Nuclear magnetic resonance spectroscopy (NMR)

Solution ^{1}H and ^{13}C nuclear magnetic resonance spectra were collected using either a Varian Inova 500 MHz spectrometer operating at 500 MHz for ^{1}H nuclei and 125 MHz for ^{13}C nuclei or a Varian VNMRS 500 MHz spectrometer operating at the same frequencies. Chemical shifts are reported relative to $CDCl_3$ (δ = 7.26 ppm for ^{1}H; δ = 77.16 ppm for ^{13}C). Exceptionally, in the case where the stereochemistry of a product was determined by examining J_{CH} couplings (section S9.), a Bruker AVIIIHD 800 MHz spectrometer was used.

S4.2. Mass spectrometry (MS)

GCMS data was obtained using a Bruker Scion single quadrupole GC/MS. APCI-MS data was obtained using a Bruker Maxis Impact API QqTOF spectrometer.

S4.3. Thermogravimetric Analysis (TGA)

Thermogravimetric analysis was performed using a Mettler Toledo TGA/DSC 1 instrument. The samples (approximately 10 mg each) were placed in 70 µL open-top alumina crucibles and measurements were conducted under a stream of nitrogen gas (50 ml min^{-1}) from room temperature to 200 °C at a rate of 10 °C min^{-1}.

S4.4. Powder x-ray diffraction (PXRD)

Powder X-ray diffraction data were collected using a Bruker D2 PHASER diffractometer which was outfitted with a LynxEye linear position-sensitive detector using nickel-filtered CuK_α X-ray radiation.

S4.5. Single crystal X-ray crystal structure determination

Single crystal X-ray diffraction (scXRD) data were measured on a Bruker D8 Venture diffractometer equipped with a Photon 200 area detector, and *IμS* microfocus X-ray source (Bruker AXS, Cu*K*α source). Measurements were carried out at 180(2) K for **1**, **1·MeNO$_2$**, **1·acetic acid**, and **1·acrylic acid**, and 153(2) K for **1·DMA,** while the rest were mounted at 298(2) K. Crystals were coated with a thin layer of paratone oil before mounting on the diffractometer. Structure solution was carried out using the SHELXTL package.[10] The parameters were refined for all data by full-matrix-least-squares refinement of F^2 using SHELXL.[11] The structures **1·MeCN**, **1·acetone**, **1·2-butanone**, **1·acetic acid**, **1·DMSO**, **1·acetaldehdye**, and **1·cyclobutanone** exhibit disordered moieties that were modeled successfully. Due to the large thermal motion in **1·MeCN,** the atoms of the included guest molecules were modeled isotropically. The crystal structure of compound **4** was determined from a single crystal that was found to be twinned by inversion. Crystalline compounds **1·MeCN**, **1·MeNO$_2$**, **1·acetone**, **1·acetic acid**, **1·acrylic acid**, **1·DMSO**, **1·NMP**, **1·DMF**, **1·isobutyraldehyde**, and **1·propionaldehyde** contain disordered guest molecules that are positioned on a 3-fold screw axis, and therefore were challenging to model. The electron densities corresponding to these symmetry-disordered guests were subtracted using the SQUEEZE procedure, as included in the PLATON software package.[12] All of the non-hydrogen atoms were refined with anisotropic thermal parameters, and the coordinates of all hydrogen atoms were constrained to ride on their carrier atom.

Crystallographic data in CIF format for all herein determined crystal structures can be accessed using the joint CCDC/FIZ Karlsruhe online deposition service (www.ccdc.cam.ac.uk/structures/), under the deposition numbers 2095086-2095103.

Table S4.1. Crystallographic data for herein determined single crystal structures.

Compound	1	1·MeCN	1·MeNO$_2$	1·acetone	1·2-butanone	1·acetic acid	1·acrylic acid	1·DMSO	1·NMP	1·DMF
**Empirical formula**	$C_{19}H_{16}Br_3P$	$C_{21}H_{19}Br_3NP$	$C_{20}H_{19}Br_3NO_2P$	$C_{22}H_{22}Br_3OP$	$C_{23}H_{24}Br_3OP$	$C_{21}H_{20}Br_3O_2P$	$C_{22}H_{20}Br_3O_2P$	$C_{21}H_{22}Br_3OPS$	$C_{24}H_{25}Br_3NOP$	$C_{22}H_{23}Br_3NOP$
M_r	515.02	556.07	576.06	573.09	587.12	575.07	587.08	593.14	614.15	588.11
T/K	180(2)	298(2)	180(2)	298(2)	298(2)	180(2)	180(2)	298(2)	298(2)	298(2)
**Crystal system**	monoclinic	trigonal	trigonal	trigonal	trigonal	trigonal	trigonal	trigonal	trigonal	trigonal
**Space group**	*C*2/c	*R*-3	*R*-3	*R*-3	*R*-3	*R*-3	R-3	*R*-3	*R*-3	*R*-3
a/Å	17.7546(4)	33.1499(5)	32.270(2)	33.0003(6)	33.4263(4)	32.4936(8)	32.283(2)	33.4643(5)	33.612(2)	33.1823(9)
b/Å	15.5320(3)	33.1499(5)	32.270(2)	33.0003(6)	33.4263(4)	32.4936(8)	32.283(2)	33.4643(5)	33.612(2)	33.1823(9)
c/Å	28.3321(6)	11.1629(2)	11.3230(8)	11.1350(2)	11.2099(2)	11.3322(8)	11.5522(8)	11.1613(2)	11.4292(7)	11.4506(3)
α/°	90	90	90	90	90	90	90	90	90	90
β/°	99.228(1)	90	90	90	90	90	90	90	90	90
γ/°	90	120	120	120	120	120	120	120	120	120
V/Å^3	7711.9(3)	10623.6(4)	10211.4(15)	10501.6(4)	10847.0(3)	10361.9(9)	10426.7(15)	10824.5(4)	11182.4(17)	10918.7(7)
Z	16	18	18	18	18	18	18	18	18	18
ρ_{calc}/g cm^{-3}	1.774	1.565	1.686	1.631	1.618	1.659	1.683	1.638	1.642	1.610
μ/mm^{-1}	8.506	7.007	7.381	7.126	6.914	7.259	7.230	7.725	6.750	6.883
F(000)	4000.0	4896.0	5076.0	5076.0	5220.0	5076.0	5184.0	5256.0	5472.0	5220.0
**2θ range for data collection/°**	6.32 to 144.588	8.498 to 144.72	8.426 to 145.472	8.522 to 144.792	8.458 to 144.352	10.89 to 144.722	8.282 to 145.226	11.318 to 144.396	5.258 to 144.34	5.326 to 145.218
**Index ranges**	-21 ≤ h ≤ 20, -12 ≤ k ≤ 19, -34 ≤ l ≤ 34	-40 ≤ h ≤ 40, -40 ≤ k ≤ 40, -13 ≤ l ≤ 5	-39 ≤ h ≤ 39, -39 ≤ k ≤ 31, -11 ≤ l ≤ 14	-40 ≤ h ≤ 40, -40 ≤ k ≤ 40, -13 ≤ l ≤ 8	-31 ≤ h ≤ 41, -41 ≤ k ≤ 30, -8 ≤ l ≤ 13	-40 ≤ h ≤ 40, -40 ≤ k ≤ 40, -13 ≤ l ≤ 9	-28 ≤ h ≤ 39, -39 ≤ k ≤ 37, -14 ≤ l ≤ 11	-41 ≤ h ≤ 41, -40 ≤ k ≤ 41, -8 ≤ l ≤ 13	-41 ≤ h ≤ 41, -41 ≤ k ≤ 41, -14 ≤ l ≤ 11	-40 ≤ h ≤ 40, -40 ≤ k ≤ 40, -10 ≤ l ≤ 14
**Reflections collected**	67258	25997	17041	58815	20559	26076	20408	61014	76439	74379
**Independent reflections**	7620 [R_{int} = 0.0643, R_{sigma} = 0.0295]	4593 [R_{int} = 0.0364, R_{sigma} = 0.0284]	4416 [R_{int} = 0.0366, R_{sigma} = 0.0330]	4613 [R_{int} = 0.0416, R_{sigma} = 0.0167]	4617 [R_{int} = 0.0312, R_{sigma} = 0.0254]	4448 [R_{int} = 0.0306, R_{sigma} = 0.0226]	4593 [R_{int} = 0.0539, R_{sigma} = 0.0439]	4702 [R_{int} = 0.0371, R_{sigma} = 0.0183]	4907 [R_{int} = 0.0943, R_{sigma} = 0.0277]	4798 [R_{int} = 0.0540, R_{sigma} = 0.0203]
**Data /restraints /parameters**	7620/54/415	4593/42/230	4416/18/245	4613/253/263	4617/222/266	4448/309/275	4593/0/254	4702/309/263	4907/18/272	4798/338/255
S	1.029	1.064	1.042	1.083	1.077	1.055	1.054	1.065	1.086	1.056
**Final R values (I≥2σ_I)**	R_1 = 0.0328, wR_2 = 0.0806	R_1 = 0.0372, wR_2 = 0.0977	R_1 = 0.0278, wR_2 = 0.0647	R_1 = 0.0291, wR_2 = 0.0655	R_1 = 0.0291, wR_2 = 0.0700	R_1 = 0.0333, wR_2 = 0.0841	R_1 = 0.0411, wR_2 = 0.0957	R_1 = 0.0393, wR_2 = 0.1037	R_1 = 0.0419, wR_2 = 0.1021	R_1 = 0.0445, wR_2 = 0.1179
**Final R values [all data]**	R_1 = 0.043, wR_2 = 0.088	R_1 = 0.041, wR_2 = 0.101	R_1 = 0.031, wR_2 = 0.067	R_1 = 0.036, wR_2 = 0.071	R_1 = 0.032, wR_2 = 0.072	R_1 = 0.0352, wR_2 = 0.0859	R_1 = 0.046, wR_2 = 0.010	R_1 = 0.041, wR_2 = 0.106	R_1 = 0.074, wR_2 = 0.137	R_1 = 0.055, wR_2 = 0.132
**Largest diff. peak/hole / e Å^{-3}**	0.69/-0.47	0.69/-0.54	0.47/-0.64	1.44/-0.44	0.41/-0.52	0.90/-0.59	2.95/-0.78	1.20/-0.81	0.39/-0.63	0.65/-0.66

Table S4.2. Crystallographic data for herein determined single crystal structures.

Compound	1·DMA	1·acetaldehyde	1·isobutyraldehyde	1·cyclobutanone	1·propionaldehyde	2	3	4
Empirical formula	$C_{23}H_{25}Br_3NOP$	$C_{21}H_{20}Br_3OP$	$C_{22}H_{22}Br_3O_{0.75}P$	$C_{23}H_{22}Br_3OP$	$C_{42}H_{41}Br_6OP_2$	$C_{19}H_{16}Cl_3P$	$C_{20}H_{19}I_3NO_2P$	$C_{19}H_{17}I_2P$
M_r	602.14	559.07	569.09	585.10	1103.15	381.64	717.03	530.09
T/K	153(2)	298(2)	298(2)	298(2)	298(2)	298(2)	298(2)	298(2)
Crystal system	trigonal	trigonal	trigonal	trigonal	trigonal	orthorhombic	trigonal	orthorhombic
Space group	*R*-3	*R*-3	*R*-3	*R*-3	*R*-3	*Pnma*	*R*-3	$Pca2_1$
a/Å	33.0726(18)	32.8339(5)	33.1440(5)	33.2157(4)	32.8602(12)	12.2883(2)	32.9862(15)	14.8074(2)
b/Å	33.0726(18)	32.8339(5)	33.1440(5)	33.2157(4)	32.8602(12)	12.5399(2)	32.9862(15)	12.4892(2)
c/Å	11.3690(6)	11.1368(2)	11.4650(2)	11.3174(2)	11.2660(4)	12.1077(2)	12.0449(8)	20.5366(3)
α/°	90	90	90	90	90	90	90	90
β/°	90	90	90	90	90	90	90	90
γ/°	120	120	120	120	120	90	120	90
V/Å^3	10769.3(13)	10397.7(4)	10907.2(4)	10813.4(3)	10535.1(9)	1865.72(5)	11350.1(13)	3797.89(10)
Z	18	18	18	18	9	4	18	8
ρ_{calc} /g cm^{-3}	1.671	1.607	1.560	1.617	1.565	1.359	1.888	1.854
μ/mm^{-1}	6.993	7.182	6.849	6.936	7.062	5.214	29.905	26.752
F(000)	5364.0	4932.0	5040.0	5184.0	4869.0	784.0	6048.0	2016.0
2θ range for data collection/°	8.368 to 144.926	9.33 to 144.818	10.676 to 144.874	9.222 to 144.97	9.322 to 144.686	10.156 to 144.696	5.358 to 144.13	7.078 to 144.93
Index ranges	-40 ≤ h ≤ 40, -40 ≤ k ≤ 40, -14 ≤ l ≤ 12	-40 ≤ h ≤ 40, -40 ≤ k ≤ 40, -13 ≤ l ≤ 13	-40 ≤ h ≤ 40, -40 ≤ k ≤ 40, -14 ≤ l ≤ 10	-41 ≤ h ≤ 40, -40 ≤ k ≤ 41, -13 ≤ l ≤ 13	-33 ≤ h ≤ 40, -40 ≤ k ≤ 40, -5 ≤ l ≤ 13	-15 ≤ h ≤ 15, -15 ≤ k ≤ 15, -14 ≤ l ≤ 14	-40 ≤ h ≤ 40, -39 ≤ k ≤ 40, -14 ≤ l ≤ 11	-16 ≤ h ≤ 18, -15 ≤ k ≤ 15, -25 ≤ l ≤ 25
Reflections collected	70134	71554	74083	71483	25610	33961	23348	76797
Independent reflections	4728 [R_{int} = 0.0379, R_{sigma} = 0.0146]	4570 [R_{int} = 0.0601, R_{sigma} = 0.0218]	4764 [R_{int} = 0.0488, R_{sigma} = 0.0198]	4760 [R_{int} = 0.0421, R_{sigma} = 0.0163]	4601 [R_{int} = 0.0644, R_{sigma} = 0.0522]	1936 [R_{int} = 0.0645, R_{sigma} = 0.0201]	4966 [R_{int} = 0.1053, R_{sigma} = 0.0679]	7463 [R_{int} = 0.0464, R_{sigma} = 0.0242]
Data /restraints /parameters	4728/12/265	4570/219/257	4764/225/254	4760/30/275	4601/221/244	1936/0/112	4966/24/248	7463/25/398
S	1.067	1.046	1.046	1.053	1.029	1.053	1.047	1.054
Final R values (I≥2σ_I)	R_1 = 0.030, wR_2 = 0.069	R_1 = 0.031, wR_2 = 0.091	R_1 = 0.037, wR_2 = 0.089	R_1 = 0.032, wR_2 = 0.100	R_1 = 0.055, wR_2 = 0.160	R_1 = 0.037, wR_2 = 0.083	R_1 = 0.060, wR_2 = 0.152	R_1 = 0.022, wR_2 = 0.053
Final R values (all data)	R_1 = 0.0309, wR_2 = 0.0701	R_1 = 0.0382, wR_2 = 0.0980	R_1 = 0.0420, wR_2 = 0.0945	R_1 = 0.0356, wR_2 = 0.1052	R_1 = 0.0662, wR_2 = 0.1764	R_1 = 0.0539, wR_2 = 0.1000	R_1 = 0.0976, wR_2 = 0.1826	R_1 = 0.0232, wR_2 = 0.0538
Largest diff. peak/hole / e Å^{-3}	0.70/-0.48	1.09/-0.42	0.71/-0.86	1.20/-0.57	1.61/-0.79	0.30/-0.23	3.72/-1.51	0.95/-0.75

S5. Powder X-ray diffraction data

S5.1. PXRD patterns of 1•guest materials made *via* recrystallisation from solution

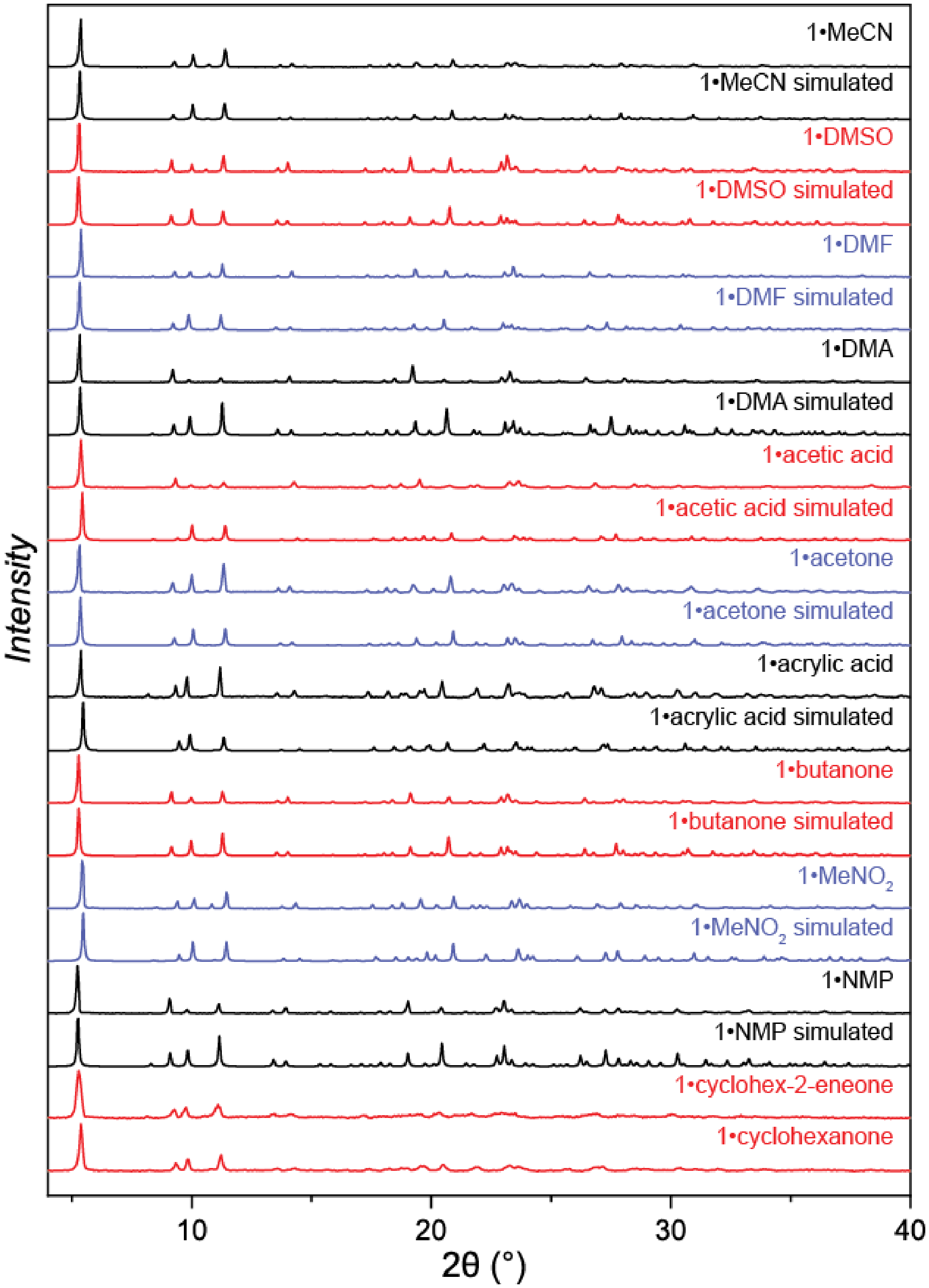

Figure S5.1. Comparisons of PXRD patterns of **1•guest** materials grown from solution. PXRD patterns simulated from scXRD crystal structures are shown for each material, if available.

S5.2. PXRD patterns of 1•reactive-guest materials made *via* soaking

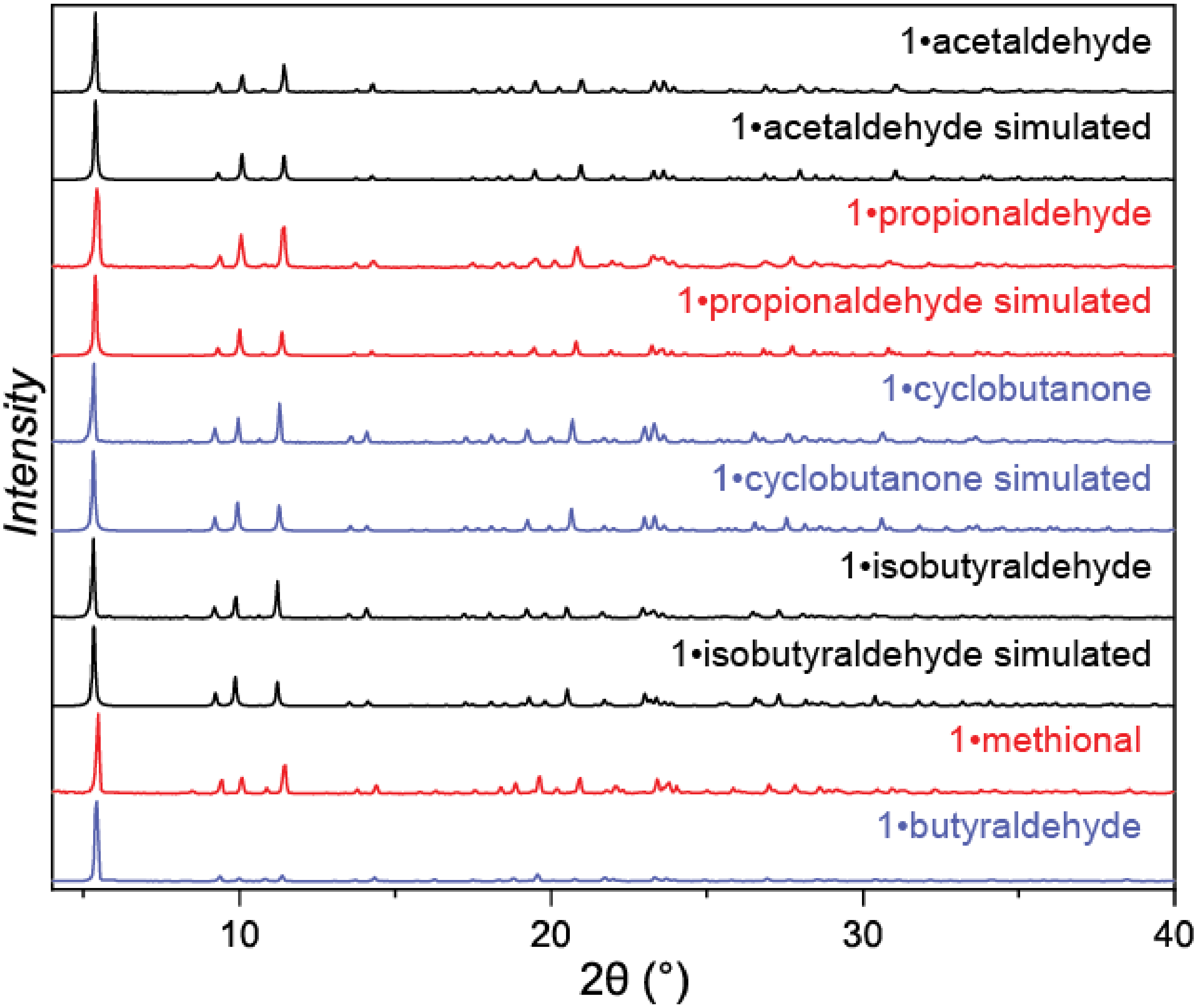

Figure S5.2. Comparisons of PXRD patterns of **1•reactive-guest** materials made by soaking of the apohost **1** in respective guest liquids. PXRD patterns simulated from scXRD crystal structures are shown for each material, if available.

S5.3. PXRD patterns of reactive 1•guest materials made *via* milling

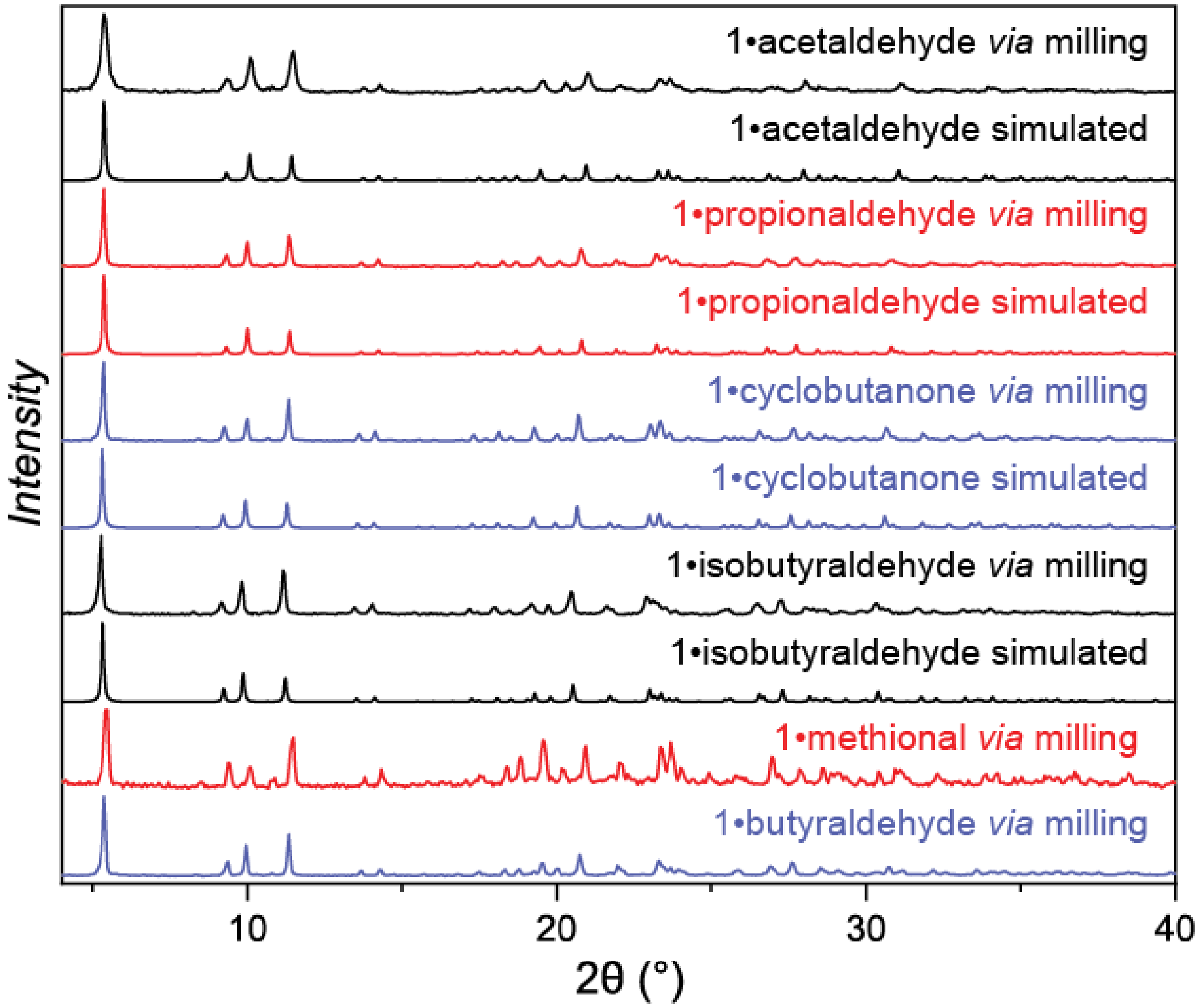

Figure S5.3. Comparisons of PXRD patterns of 1•**reactive-guest** made by milling of apohost **1** with respective guests. PXRD patterns simulated from scXRD crystal structures are shown for each material, if available.

S5.4. PXRD patterns of 1 after solvation, desolvation, and milling

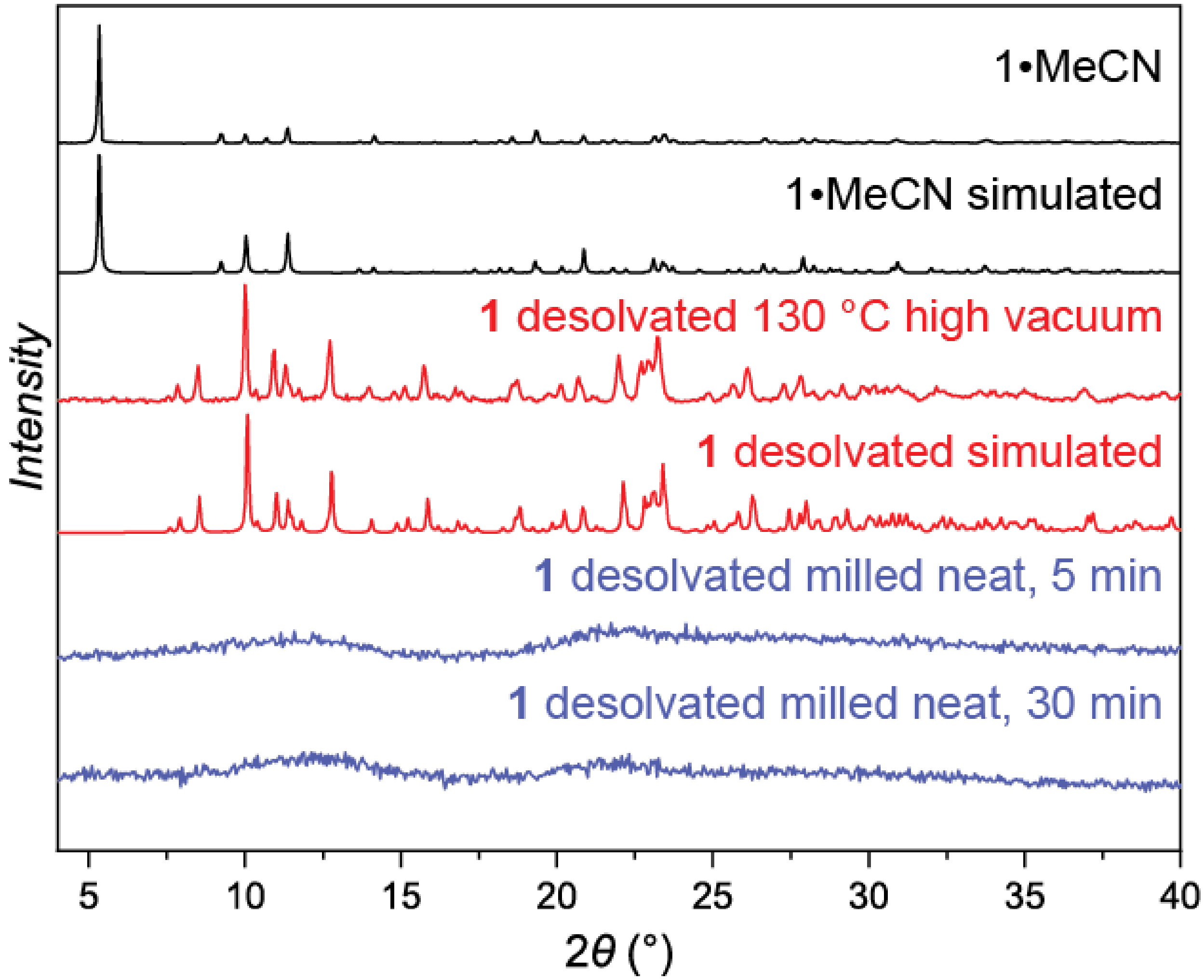

Figure S5.4. Comparisons of PXRD patterns of **1** (from top to bottom): solvated with MeCN; desolvated using heat *in vacuo*; amorphous due to milling.

S5.5. PXRD patterns of 1•butyraldehyde and the crystalline byproducts of the Wittig olefination using 1•butyraldehyde

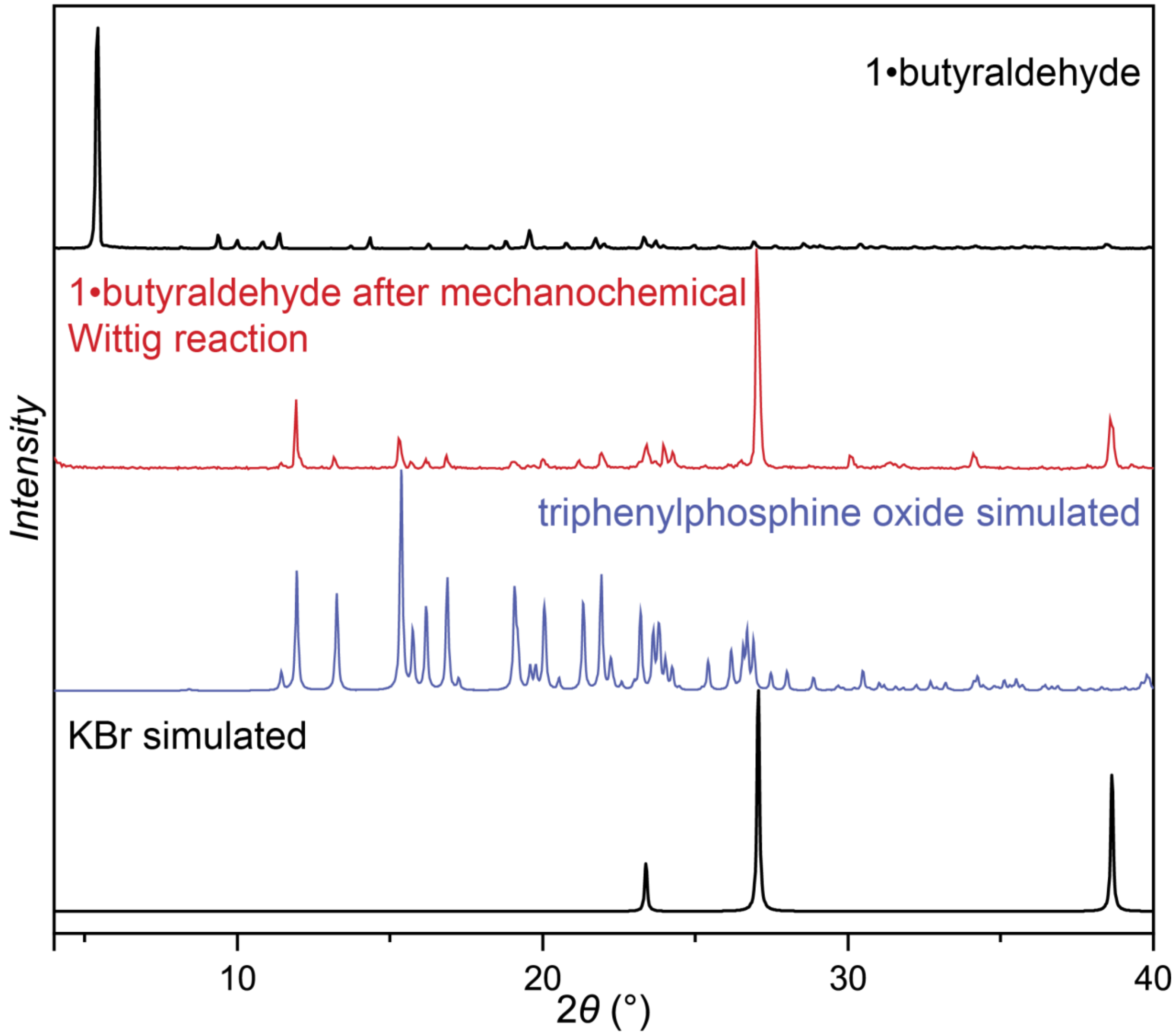

Figure S5.5. Comparison of PXRD patterns of **1•butyraldehyde** before and after Wittig olefination with potential crystalline byproducts of the reaction (from top to bottom): **1•butyraldehyde**; crude reaction product after Wittig olefination using **1•butyraldehyde**; triphenylphosphine oxide[13] simulated; KBr simulated.[14]

S5.6. PXRD patterns of selected materials in the synthesis of 2

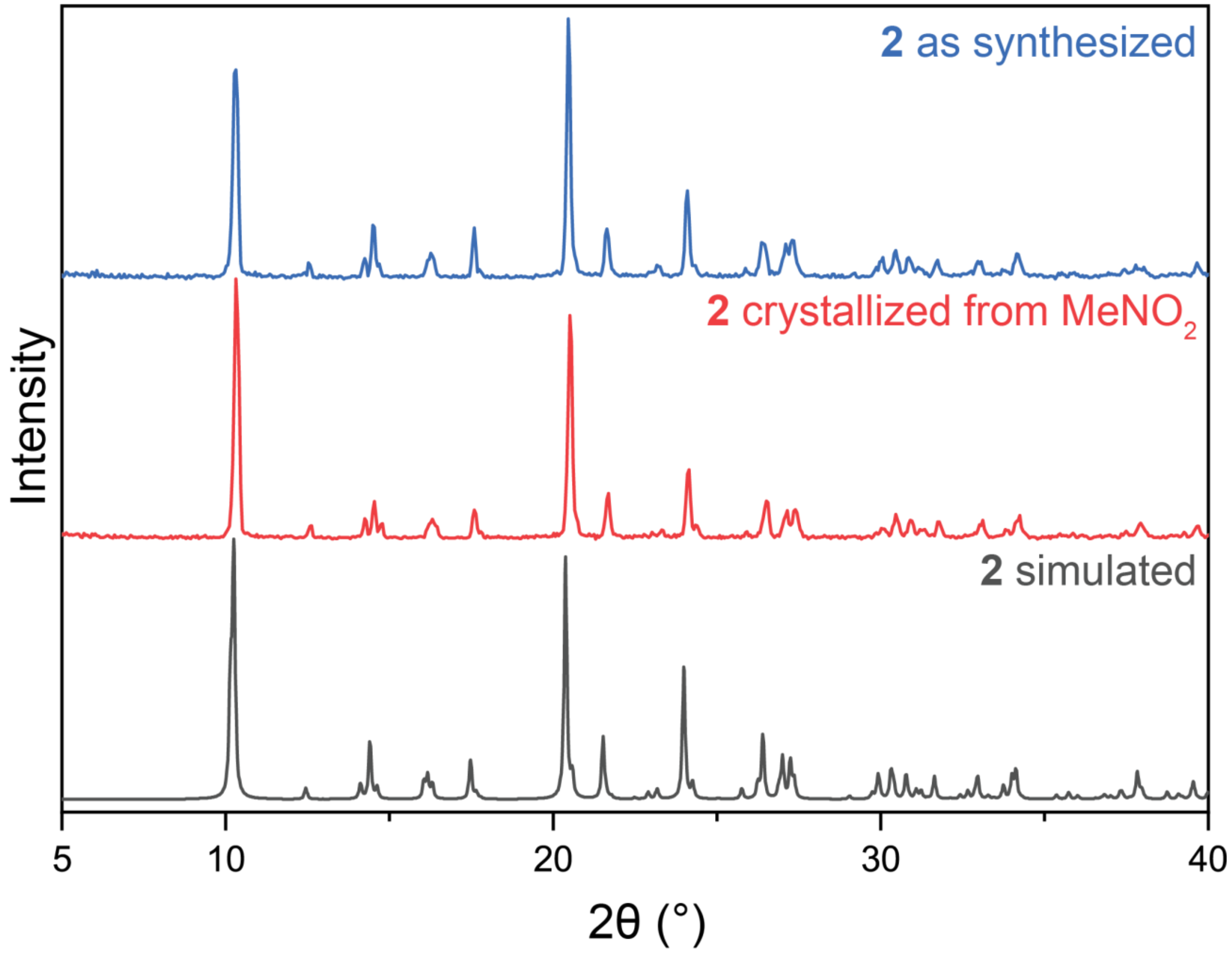

Figure S5.6. Comparison of PXRD patterns of compound **2** (from top to bottom): as-synthesized from benzene solution; after recrystallisation from $MeNO_2$; and simulated for the herein determined crystal structure.

S5.7. PXRD patterns of the selected materials in the synthesis of 3 and 4

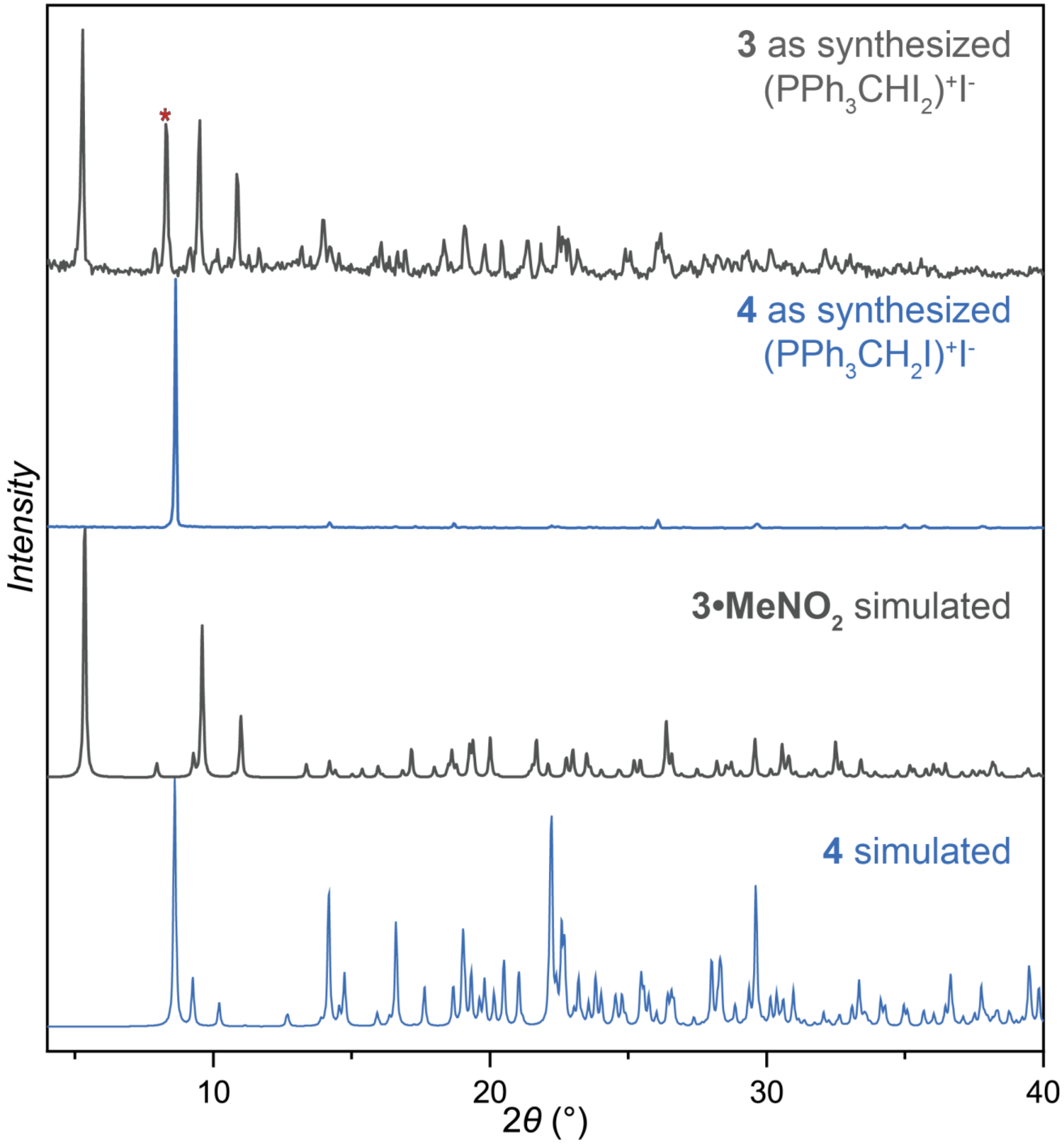

Figure S5.7. Comparison of PXRD patterns of powders of **3** and **4** (from top to bottom): bulk powdered **3** as synthesised from MeCN; bulk powdered **4** as synthesised from hot $MeNO_2$; **3•MeNO** simulated from the scXRD structure reported herein; **4** simulated from the scXRD structure reported herein.

S5.8. PXRD pattern of nonsolvated 1 milled with cyclobutanone

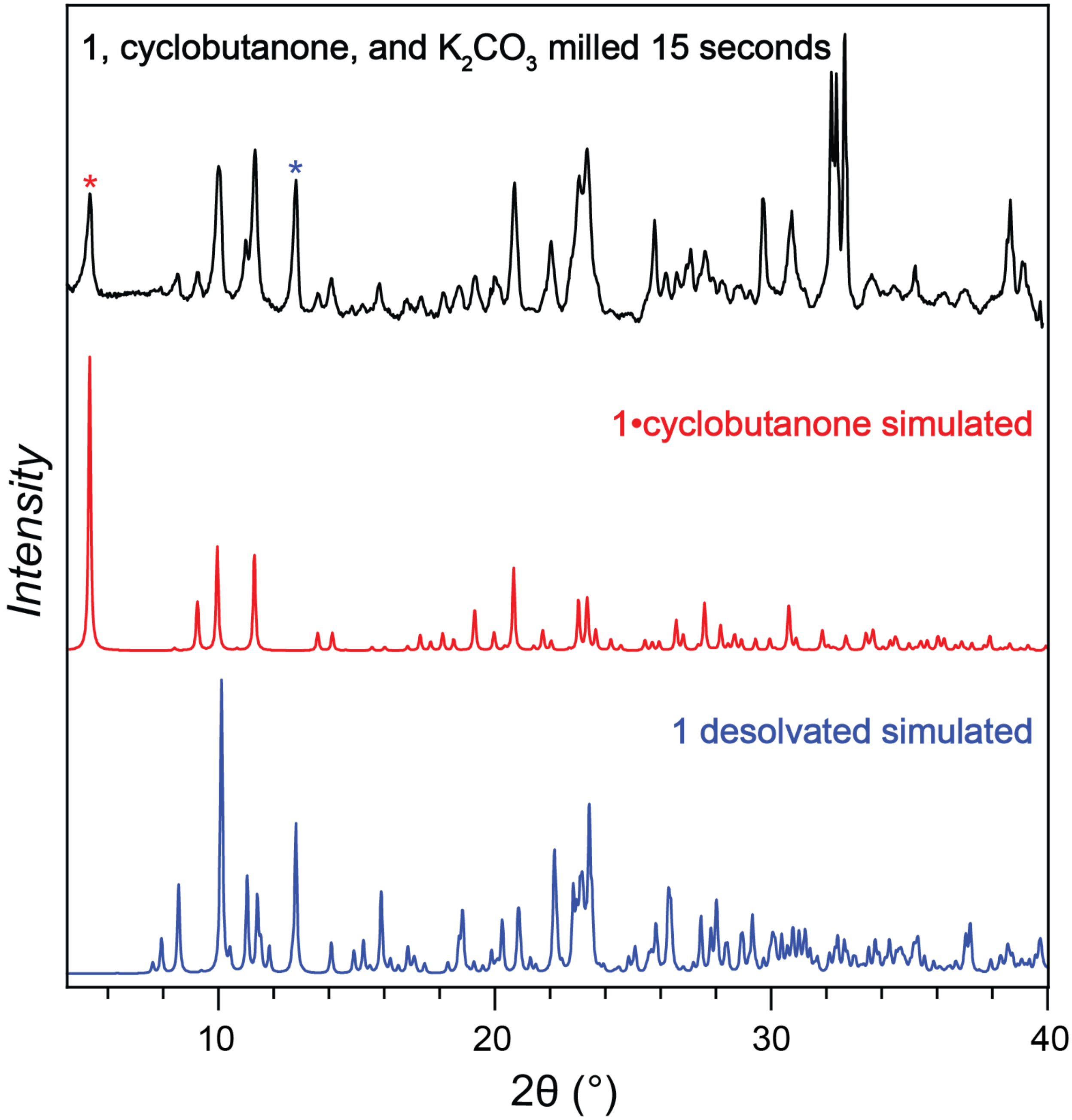

Figure S5.8. Comparison of PXRD patterns of a reaction mixture produced by milling **1**, cyclobutanone, and K_2CO_3 with patterns of **1•cyclobutanone** and apohost **1,** simulated from the respective scXRD crystal structures reported herein. X-ray reflections corresponding to **1•cyclobutanone** and apohost **1** are marked with red and blue asterisks, respectively.

S6. Fourier-transform infrared attenuated total reflectance (FTIR-ATR) spectroscopy

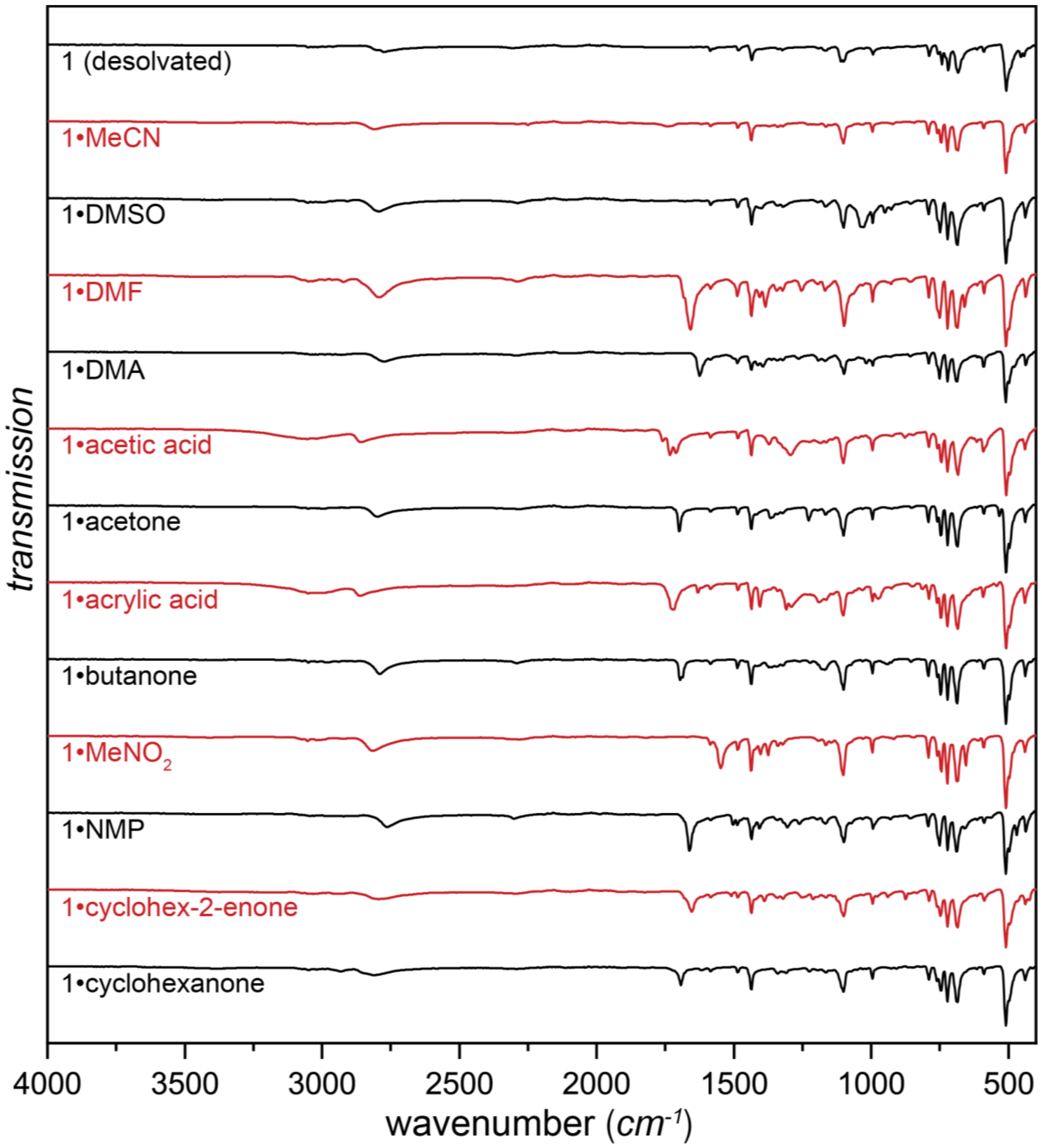

Figure S6.1. Comparison of FTIR-ATR spectra of **1•guest** materials

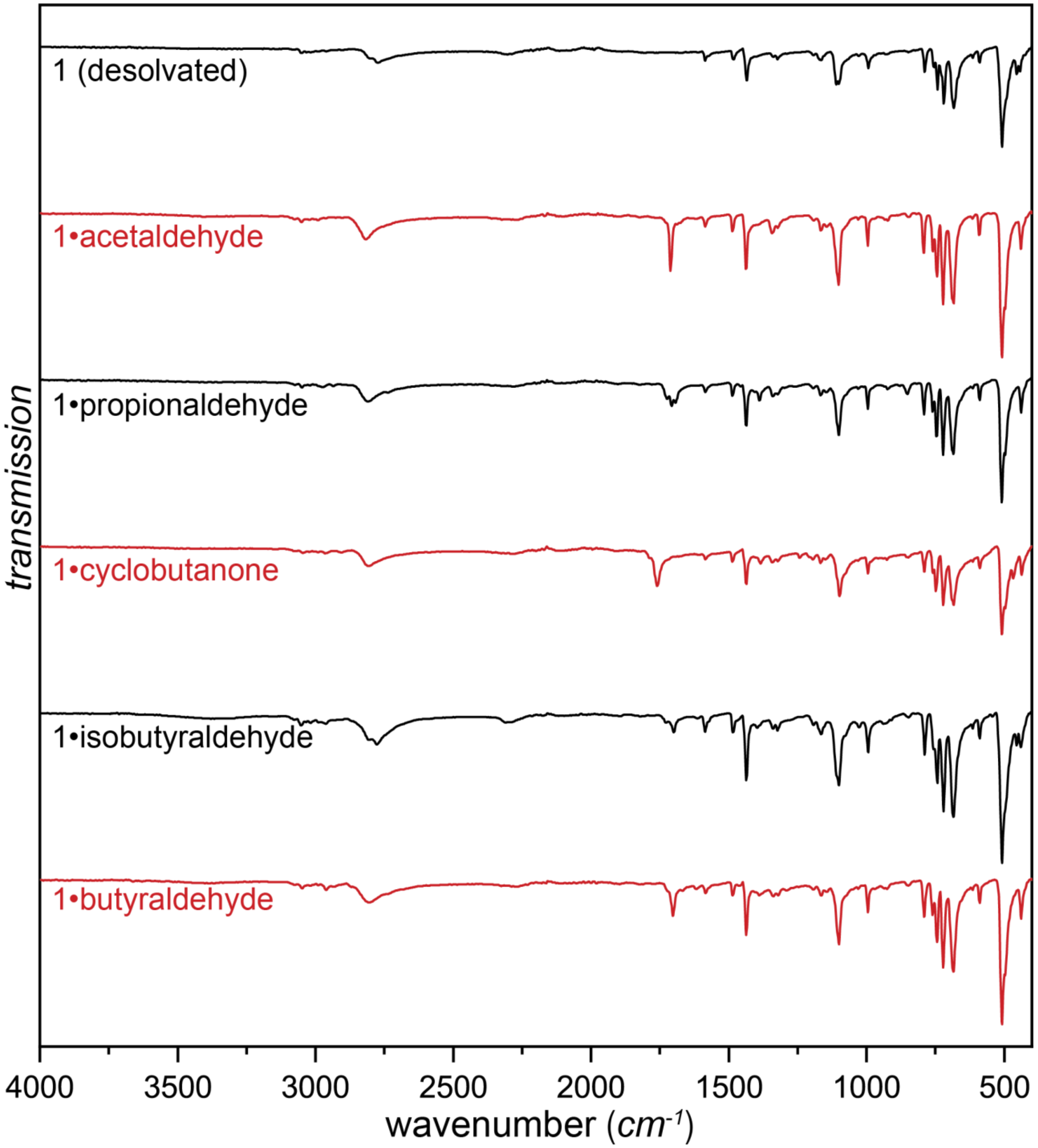

Figure S6.2. Comparison of FTIR-ATR spectra of **1•reactive-guest** materials.

S7. Thermogravimetric analysis

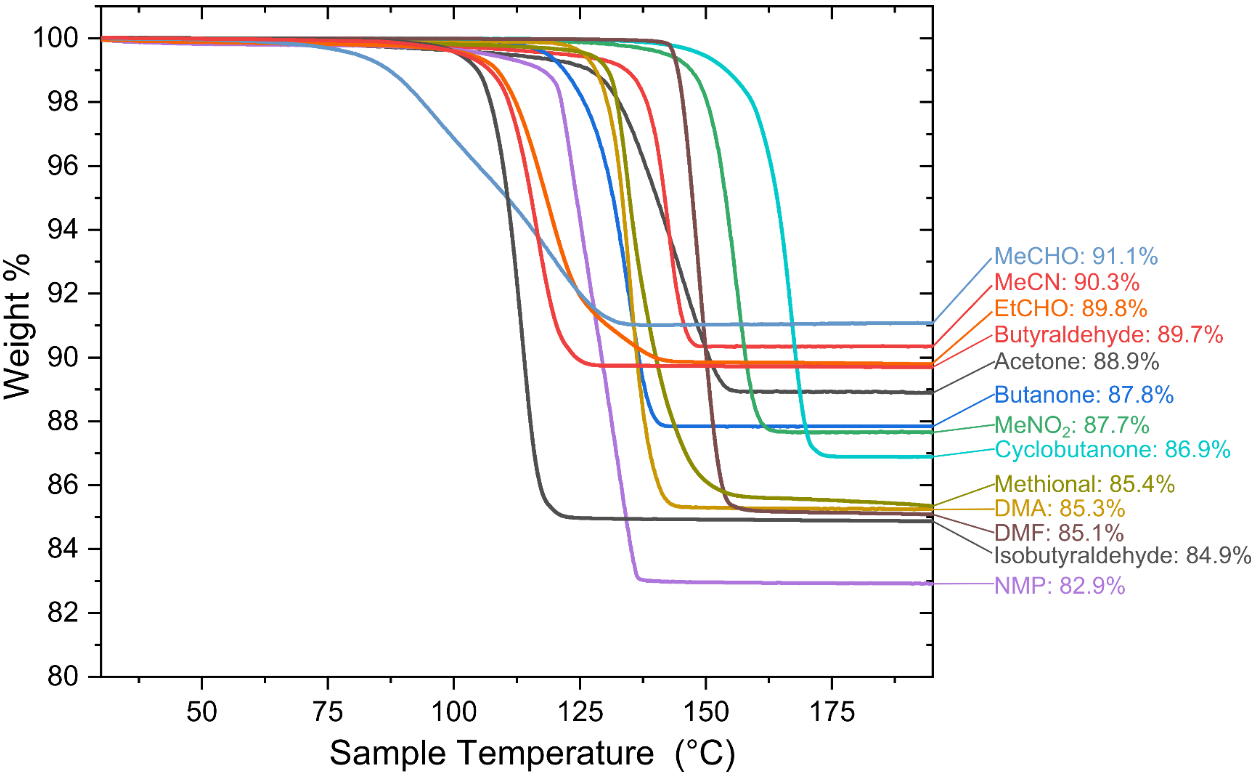

Figure S7.1. TGA thermograms **1•guest** and **1•reactive-guest** materials for which the regions before and after the initial loss of mass were sufficiently flat to allow for the calculation of guest quantities.

The number of guests per cage was calculated from TGA residues using the following equation:

$$Residue\ \% = \frac{6M_r(\mathbf{1})}{6M_r(\mathbf{1}) + nM_r(\boldsymbol{guest})} * 100$$

where:

n = number of guests per hexameric cage

$M_r(\mathbf{1})$ = molecular weight of **1**

$M_r(\boldsymbol{guest})$ = molecular weight of the **guest**

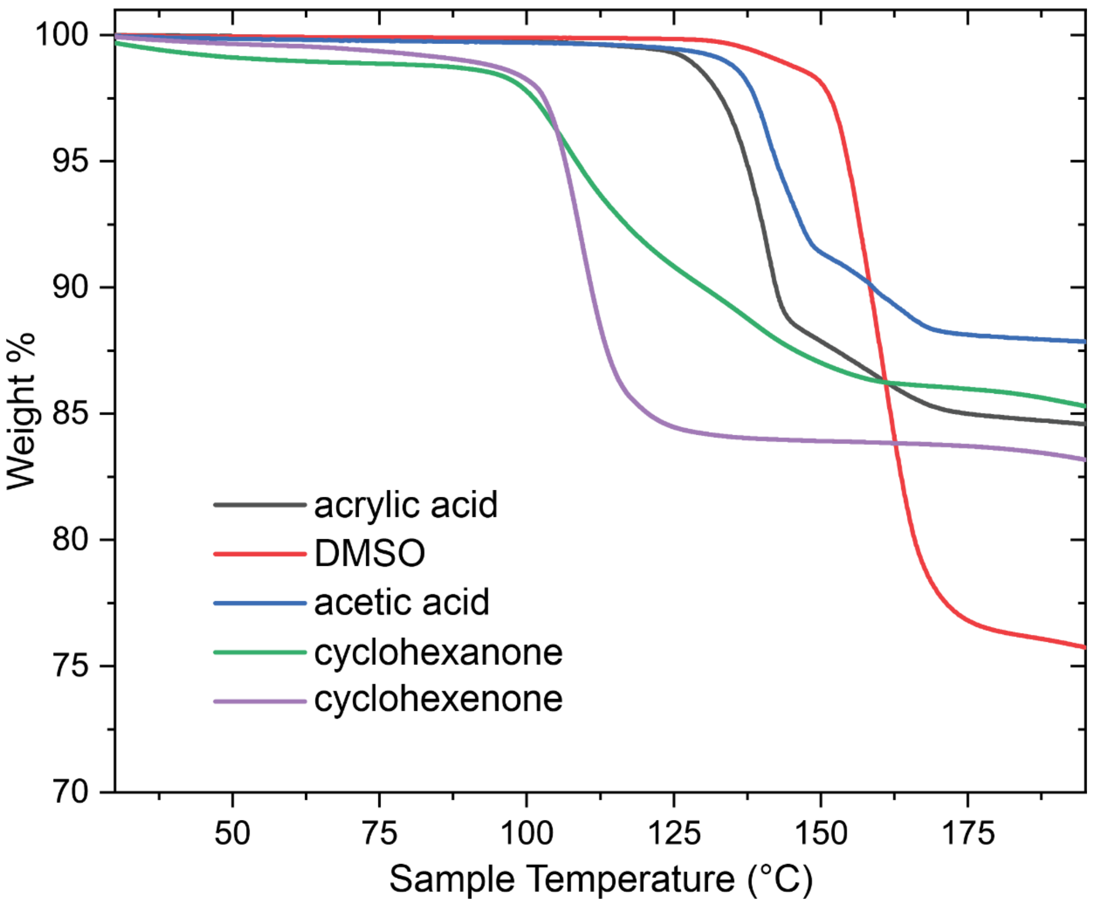

Figure S7.2. TGA thermograms **1•guest** and **1•reactive-guest** materials for which the regions before or after the initial loss of mass were not sufficiently flat to allow for the calculation of guest quantities.

S8. Number of guest molecules per cage in 1·guest materials as determined by TGA and NMR

Table S8.1. The number of guests per cage in **1·guest** materials as determined by TGA and ^{1}H NMR. The corresponding TGA data and the method for the calculation of guest loading based on it are shown in section S7**.** Calculations based on ^{1}H NMR are explained in section S11.1 and the raw data is shown in section S12.

material	guests per cage (TGA)	guests per cage (^{1}H NMR)
**1•acetaldehyde**	6.9	6.5
**1•MeCN**	8.1	6.8
**1•propionaldehyde**	6.0	5.9
**1•acetone**	6.6	5.7
**1•butanone**	6.0	5.6
**1•MeNO$_2$**	7.1	6.2
**1•cyclobutanone**	6.7	6.2
**1•methional**	5.1	4.2
**1•DMA**	6.1	6.0
**1•DMF**	7.4	6.8
**1•NMP**	6.4	6.0
**1•isobutyraldehyde**	7.7	4.3
**1•butyraldehyde**	4.9	4.4
**1•acetic acid**	-	7.1
**1•acrylic acid**	-	-*
**1•DMSO**	-	7.0
**1•cyclohex-2-enone**	-	5.4
**1•cyclohexanone**	-	4.4

*: The ^{1}H NMR for 1**•acrylic acid** contains impurities which obscure signals from acrylic acid, making the determination of the number of guests per cage unreliable.

S9. Determination of stereochemistry of eneyne product

The slice of the spectrum shown in Figure S9.1. was obtained using a selHSQMBC-TOCSY sequence[15] acquired on a Bruker AVIIIHD 800 MHz spectrometer equipped with a 5mm TCI cryoprobe. Approximately 5 mg of sample was used, dissolved in $CDCl_3$. The spectrum was acquired using the Bruker pulse program *hsqcdietgpiajclrndsp* with 8 scans for each of 128 increments, using an acquisition time of 0.4 ms and a recycle delay of 2 s, for an experiment time of 46 min. The experiment was optimized for the ^{1}H signal at 6.36 ppm and $^nJ_{CH}$ = 6 Hz using a TOCSY mixing time of 45 ms and processed using the Bruker AU program split (option ipap 2). The assignment of the unprotonated vinyl carbon was made definitively using a 1,1-ADEQUATE spectrum acquired on the same spectrometer (Bruker pulse program adeq11etgprdsp) and optimized for $^1J_{CC}$ = 80 Hz. The spectrum was acquired with 64 scans for each of 184 increments and a recycle delay of 1.7 s for a total experiment time of 6 hours.

Assignment of the stereochemistry as shown below (Z isomer) was determined by comparing J_{CH} couplings determined by DFT (see section S3.2) with those determined as described above. For example, DFT calculations yielded $^2J_{CH}$ = - 8.1 Hz (E isomer) and $^2J_{CH}$ = + 8.3 Hz (Z isomer) for the coupling between the brominated carbon and the vinyl proton. Experimental analysis (described above, spectra shown below) produced a value of $^2J_{CH}$ = +7.1 Hz, strongly suggesting Z stereochemistry for the eneyne product.

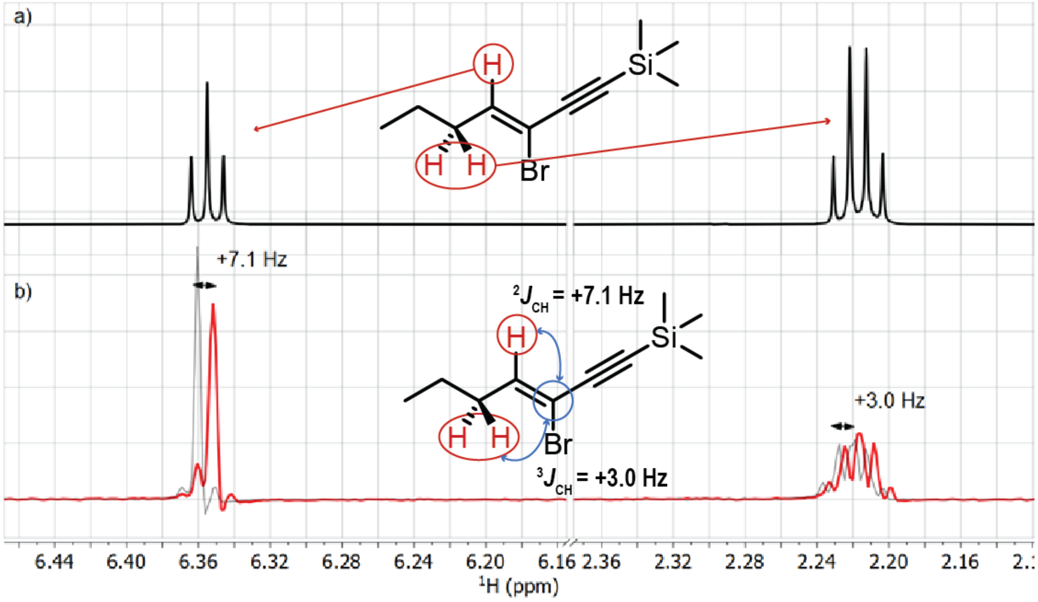

Figure S9.1. a) Extract of ^{1}H NMR spectrum of the eneyne, showing the vinyl ^{1}H (6.35 ppm) and allylic CH_2 ^{1}H (2.22 ppm). b) Processed IP/AP slices from 2D selHSQMBC-TOCSY spectrum[15] for the nonprotonated vinyl ^{13}C (102.4 ppm), showing the $^3J_{CH}$ coupling from this ^{13}C of +3.0 Hz and the $^2J_{CH}$ coupling of +7.1 Hz. The two-bond coupling must be positive as it displays the same relative shift of IP/AP slides as the three-bond coupling, which is known to be positive.

S10. Analysis of Organic Products

Raw ^{1}H and ^{13}C spectra are presented in section **S14.**

1,1-dibromopropene

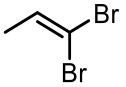

Colorless liquid isolated by distillation from the contents of the jar after milling. ^{1}H NMR (500 MHz, $CDCl_3$): 1.71 (d, J = 6.8 Hz, 3H), 6.46 (q, J = 6.8 Hz, 1H). ^{13}C NMR (125 MHz, $CDCl_3$): 18.5 (CH_3), 89.6 ($C_{dibromo}$), 133.5 (CH). GCMS (m/z): [M]+ calculated for $C_3H_4Br_2$: 199.9; found: 200.

1,1-dibromobutene

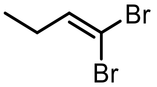

Colorless liquid, isolated by distillation from jar contents. ^{1}H NMR (500 MHz, $CDCl_3$): 1.04 (t, J = 7.5 Hz, 3H), 2.12 (p, J = 7.5 Hz, 2H), 6.40 (t, J = 7.2 Hz, 1H). ^{13}C NMR (125 MHz, $CDCl_3$): 12.3 (CH_3), 26.5 (CH_2), 88.2 ($C_{dibromo}$), 140.1 (CH). GCMS (m/z): [M]+ calculated for $C_3H_4Br_2$: 213.9; found: 214.

1,1-dibromopentene

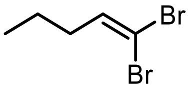

Colorless liquid, isolated by distillation from jar contents. ^{1}H NMR (500 MHz, $CDCl_3$): 0.95 (t, J = 7.4 Hz, 3H), 1.46 (h, J = 7.4 Hz, 2H), 2.09 (q, J = 7.3 Hz, 2H), 6.41 (t, J = 7.2 Hz, 1H). ^{13}C NMR (125 MHz, $CDCl_3$): 13.6 (CH_3), 21.2 (CH_2), 35.0 (CH_2), 88.6 ($C_{dibromo}$), 138.7 (CH). GCMS (m/z): [M]+ calculated for $C_5H_8Br_2$: 227.9; found: 228.

(dibromomethylene)cyclobutane

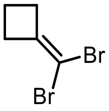

Colorless liquid, isolated by column chromatography in 100% hexanes. ^{1}H NMR (500 MHz, $CDCl_3$): 1.91 (p, J = 8.0 Hz, 2H), 2.63 (t, J = 8.0 Hz, 2H). ^{13}C NMR (125 MHz, $CDCl_3$): 13.4 (CH_2), 32.6 (CH_2), 78.9 ($C_{dibromo}$), 147.3 (C). GCMS (m/z): [M]+ calculated for $C_5H_8Br_2$: 225.9; found: 226.

1,1-dibromo-4-methylthiobutene

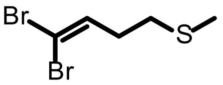

Colorless liquid, isolated by column chromatography in 100% hexanes. ^{1}H NMR (500 MHz, $CDCl_3$): 2.14 (s, 3H), 2.41 (q, J = 7.2 Hz, 2H), 2.59 (t, J = 7.3 Hz, 2H), 6.50 (t, J = 7.1 Hz, 1H). ^{13}C NMR (125 MHz, $CDCl_3$): 15.4 (CH_3), 32.0 (CH_2), 32.5 (CH_2), 90.3

(C$_{dibromo}$), 136.5 (CH). APCI-HRMS (m/z): [M+H]+ calculated for $C_5H_9Br_2S$: 258.88; found: 258.88.

eneyne Sonogashira product

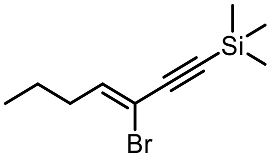

Yellow oil, isolated by column chromatography in 100% hexanes. Co-elutes with trimethylsilylacetylene homocoupling dimer, marked in NMR spectra. ^{1}H NMR (500 MHz, $CDCl_3$): 0.22 (s, 9H), 0.96 (t, J = 7.4 Hz, 3H), 1.47 (h, J = 7.3 Hz, 2H), 2.21 (q, J = 7.3 Hz, 2H), 6.35` (t, J = 7.2 Hz, 1H). ^{13}C NMR (125 MHz, $CDCl_3$): -0.3 ($CH_{3\ TMS}$), 13.7 (CH_3), 21.2 (CH_2), 34.0 (CH_2), 94.6 (C), 102.6 (C), 102.6 (C), 141.1 (CH). APCI-HRMS (m/z): [M]+ calculated for $C_{10}H_{17}BrSi$: 244.03; found: 244.03. The stereochemistry of this product was confirmed as shown in sections S3.2**.** and S9.

enediyne Sonogashira product

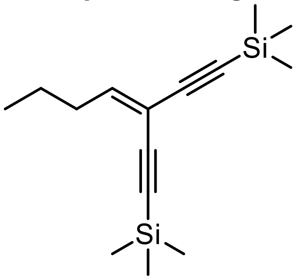

Yellow oil, isolated by column chromatography in 100% hexanes. Isolated yield: 13.7 mg (45%). ^{1}H NMR (500 MHz, $CDCl_3$): 0.20 (s, 9H), 0.22 (s, 9H), 0.95 (t, J = 7.4 Hz, 3H), 1.47 (h, J = 7.4 Hz, 2H), 2.33 (q, J = 7.4 Hz, 2H), 6.42 (t, J = 7.7 Hz, 1H). ^{13}C NMR (125 MHz, $CDCl_3$): -0.1 ($CH_{3\ TMS}$), 13.8 (CH_3), 21.7 (CH_2), 32.8 (CH_2), 91.5 (C), 98.4 (C), 99.8 (C), 102.5 (C), 106.0 (C), 151.8 (CH). APCI-HRMS (m/z): [M]+ calculated for $C_{15}H_{26}Si_2$: 262.16; found: 262.16.

enediyne Sonogashira product (thioether analogue)

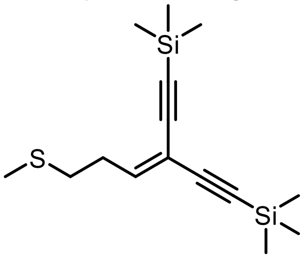

Yellow oil, isolated by column chromatography using a gradient elution from 100% hexanes to 5% ether in hexanes. ^{1}H NMR (500 MHz, $CDCl_3$): 0.20 (s, 9H), 0.22 (s, 9H), 0.95 (t, J = 7.4 Hz, 3H), 1.47 (h, J = 7.4 Hz, 2H), 2.33 (q, J = 7.4 Hz, 2H), 6.42 (t, J = 7.7 Hz, 1H). ^{13}C NMR (125 MHz, $CDCl_3$): -0.18 ($CH_{3\ TMS}$) -0.15 ($CH_{3\ TMS}$), 15.3 (CH_3), 30.3 (CH_2), 32.5 (CH_2), 92.4 (C), 99.1 (C), 99.4 (C), 102.0 (C), 107.2 (C), 148.5 (CH). APCI-HRMS (m/z): [M+H]+ calculated for $C_{15}H_{27}SSi_2$: 295.13; found: 295.13.

S11. ^{1}H NMR quantitative methods

S11.1. Calculation of the number of guests per cage

The phenyl group protons of **1** are used as an internal standard. Integrations are such that the signals which correspond to six protons of **1** are set to an integration value of 1.0. A characteristic signal of the **guest** molecule is chosen, its integration divided by the number of protons it corresponds to per each **guest** molecule, then multiplied by six to obtain the number of guests per salt **1**. Multiplying this number by 6 gives the approximate number of guest molecules per hexameric cage.

S11.2. Calculation of conversion for Wittig Olefinations and one-pot combination of Wittig Olefination and Sonogashira coupling reactions

The reaction byproduct triphenylphosphine oxide (TPPO) was used as an ^{1}H NMR internal standard to determine reaction conversions. This method relies on the fact that one equivalent of cage material produces one equivalent of TPPO during the reaction. Given the known initial quantity of cage material and guest, all product signal integrations can be compared to TPPO to calculate reaction conversion. Importantly, this method accounts for reactants or products which may be lost to processes like polymerization or decomposition. Exceptionally, in solution-based control reactions (Table S2.1) where the salt **1** had not yet fully converted to TPPO, combined ^{1}H NMR signal integrations of **1** and TPPO were used as the internal standard to determine conversions to products.

S12. ^{1}H NMR spectra of cage materials

In spectra of **1•guest** materials, the characteristic ^{1}H NMR signal for the guest which is integrated and compared to signals of the guest is denoted by a red “*” (asterisk).

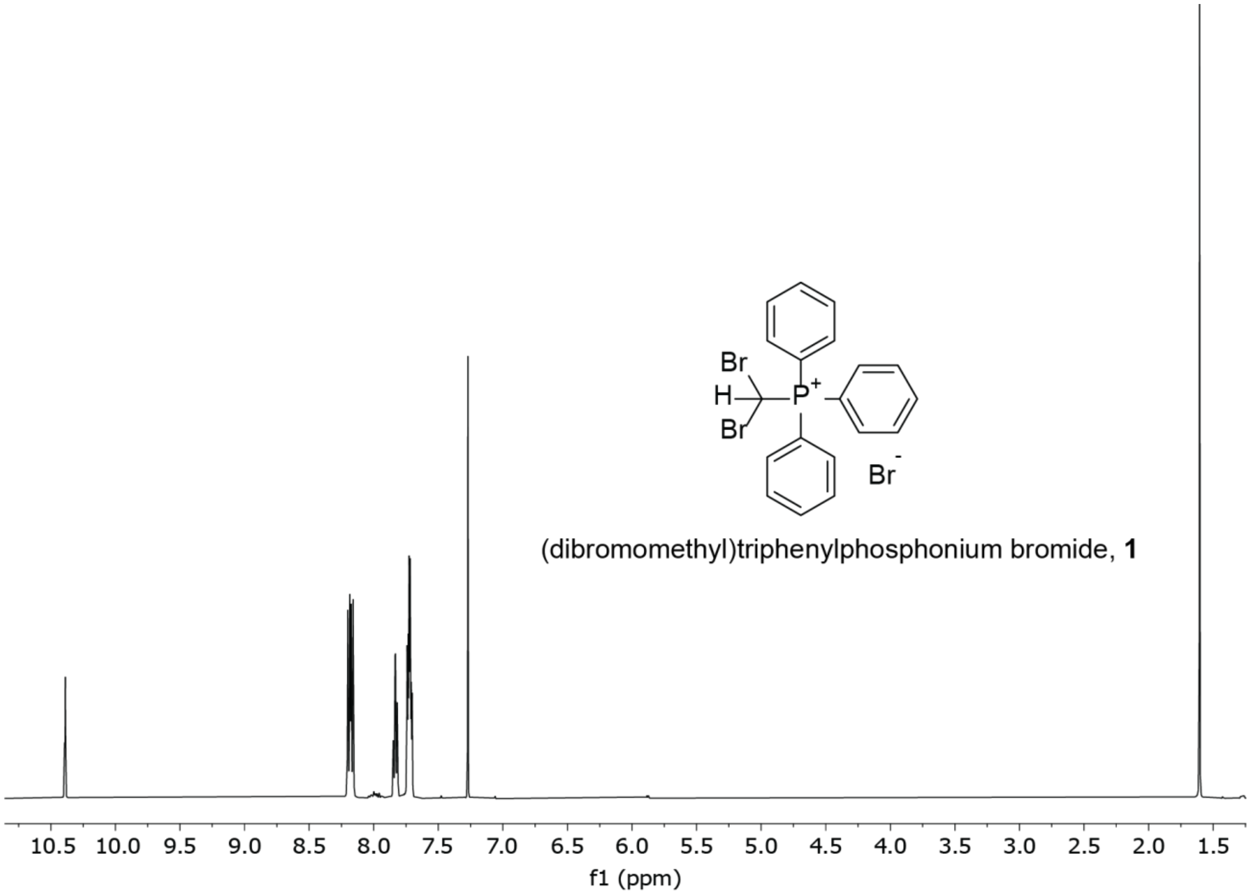

Figure S12.1. ^{1}H NMR spectrum of pure apohost **1**.

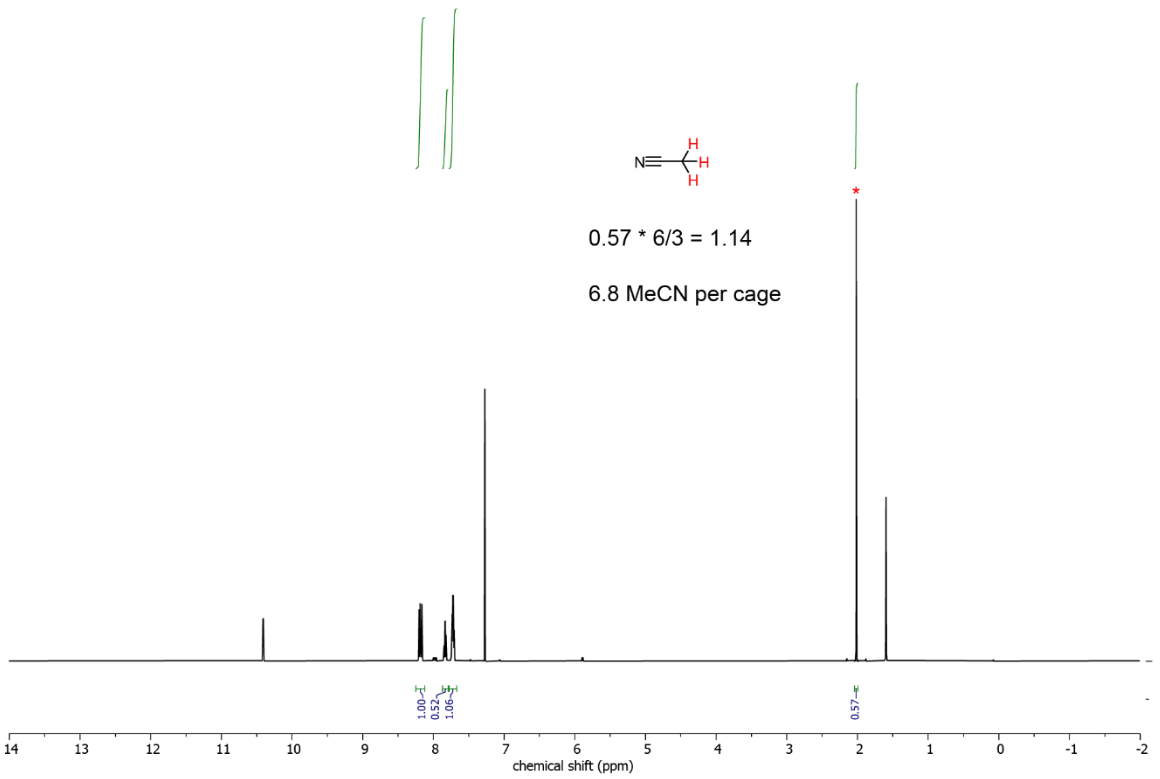

Figure S12.2. ^{1}H NMR spectrum of **1•MeCN**.

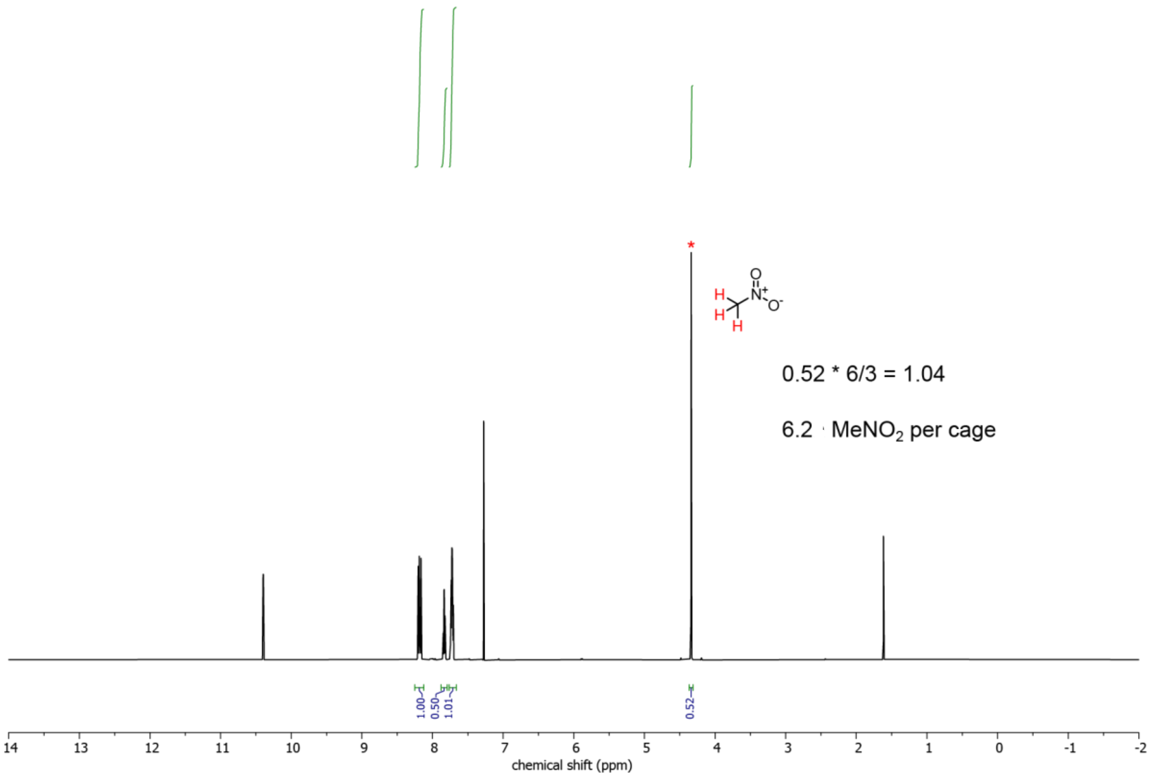

Figure S12.3. ^{1}H NMR spectrum of **1•MeNO$_2$**.

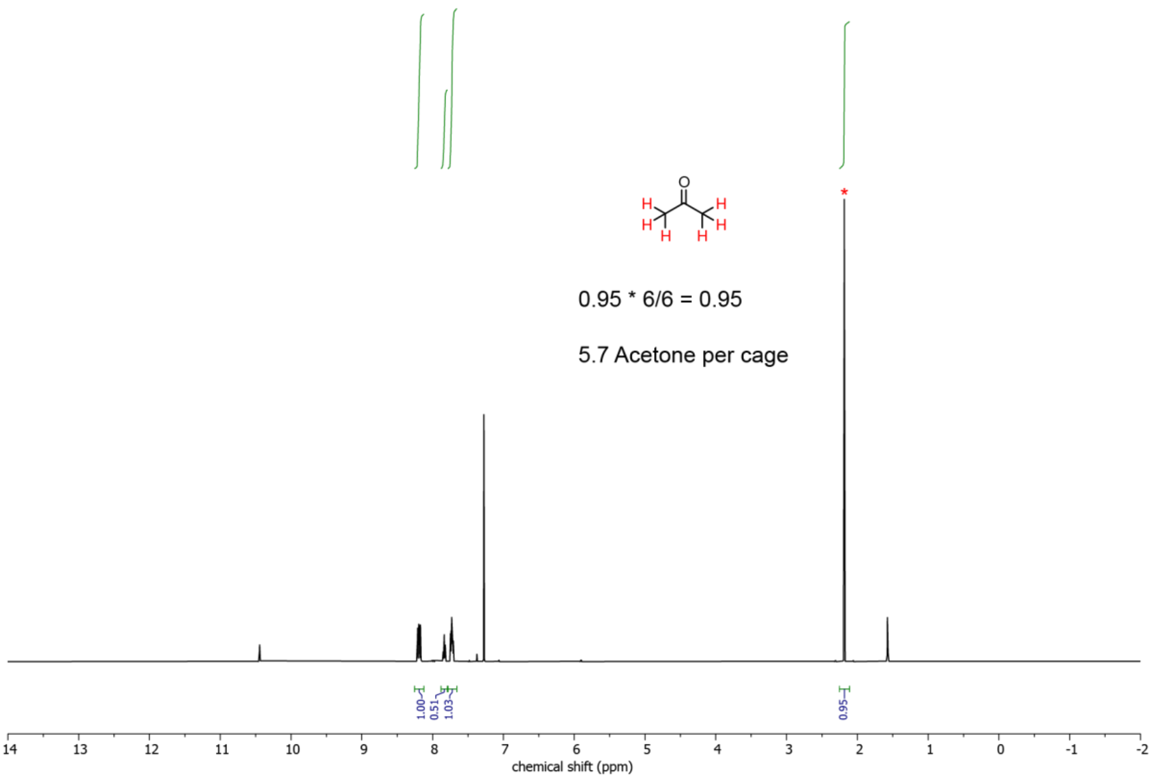

Figure S12.4. ^{1}H NMR spectrum of **1•acetone**.

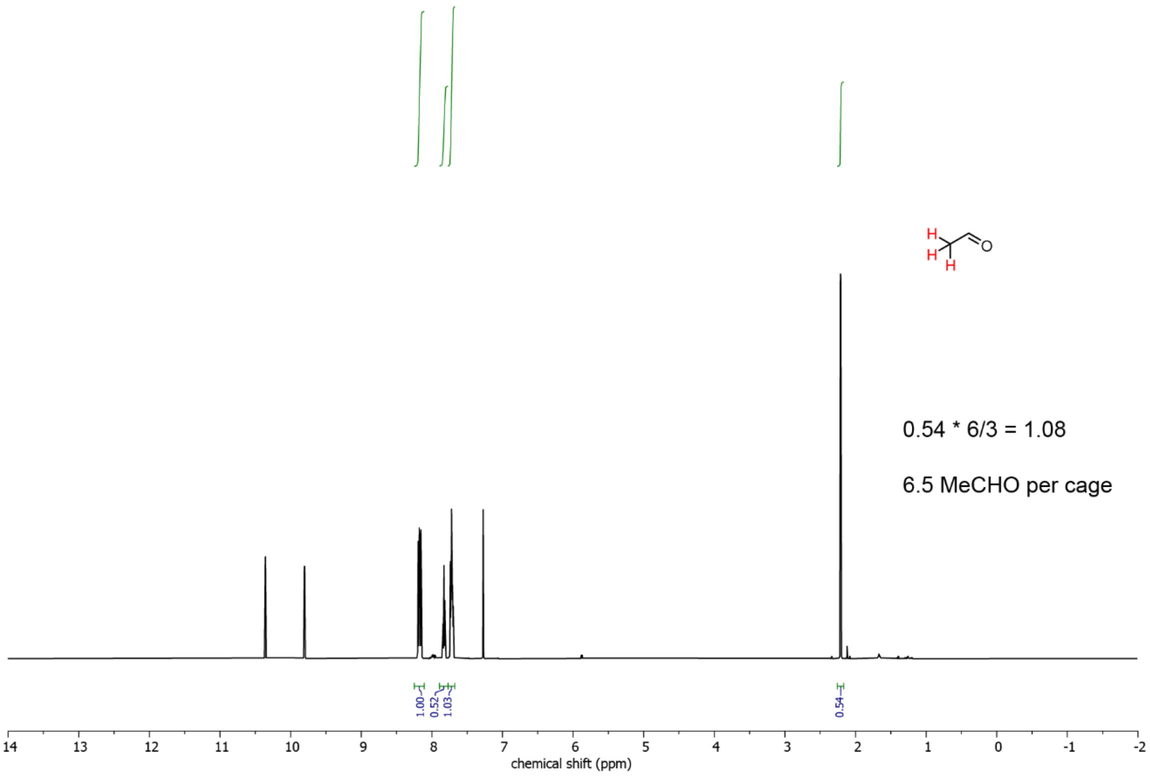

Figure S12.5. ^{1}H NMR spectrum of **1•acetaldehyde**.

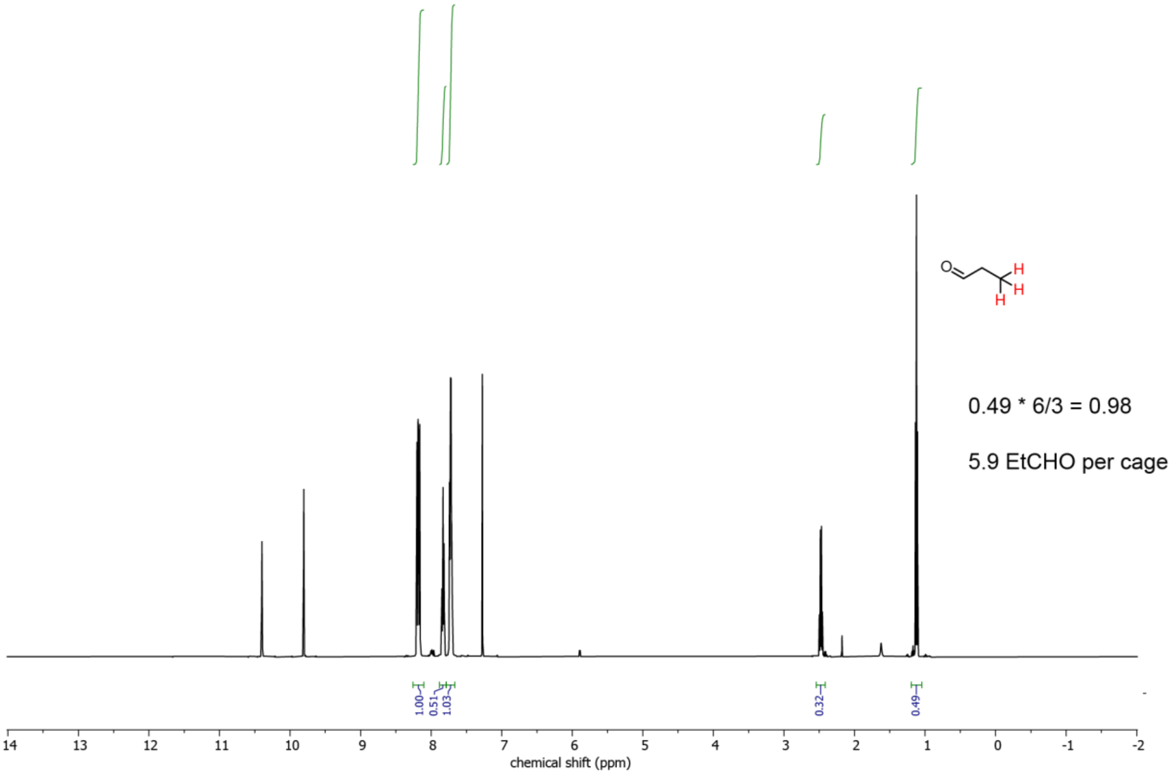

Figure S12.6. ^{1}H NMR spectrum of **1•propionaldehyde**.

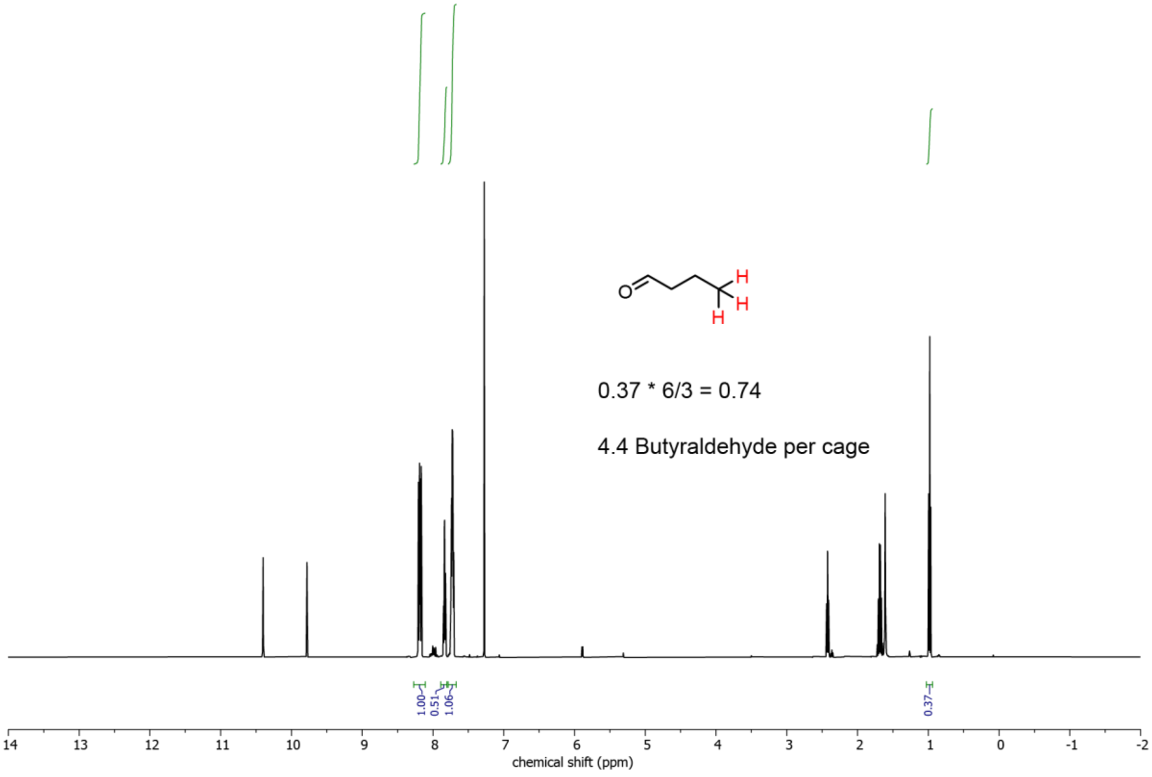

Figure S12.7. ^{1}H NMR spectrum of **1•butyraldehyde**.

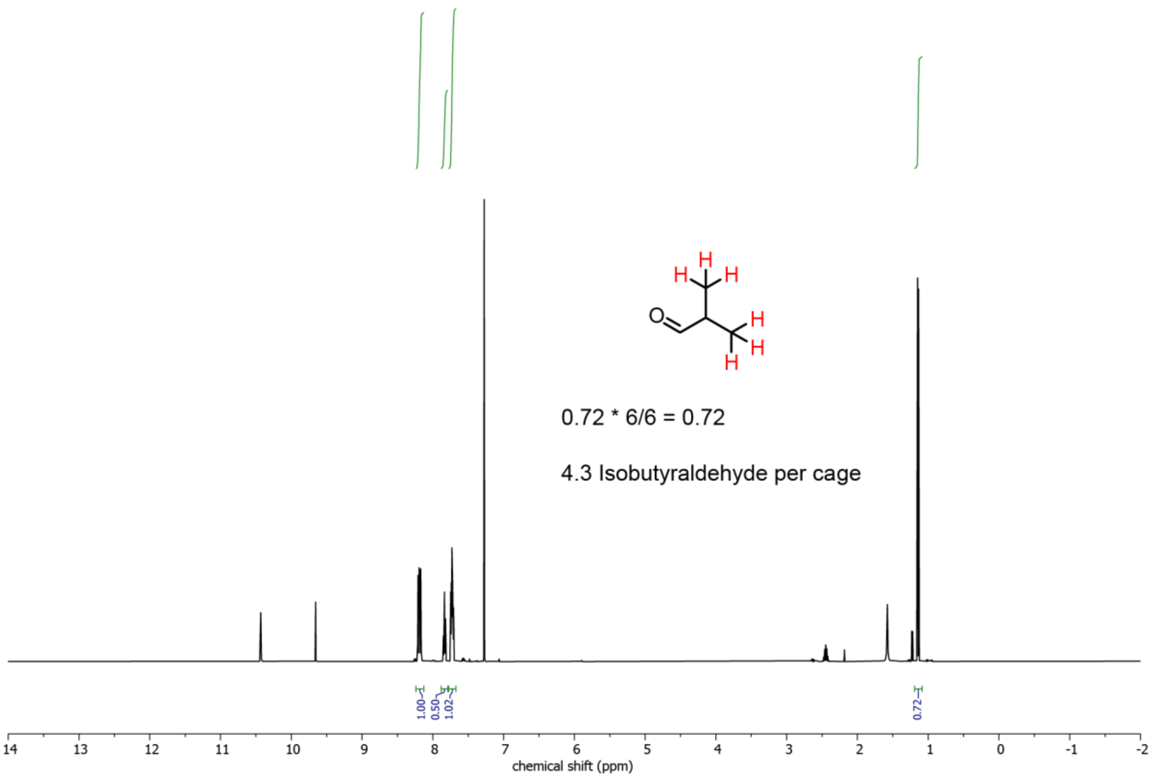

Figure S12.8. ^{1}H NMR spectrum of **1•isobutyraldehyde**.

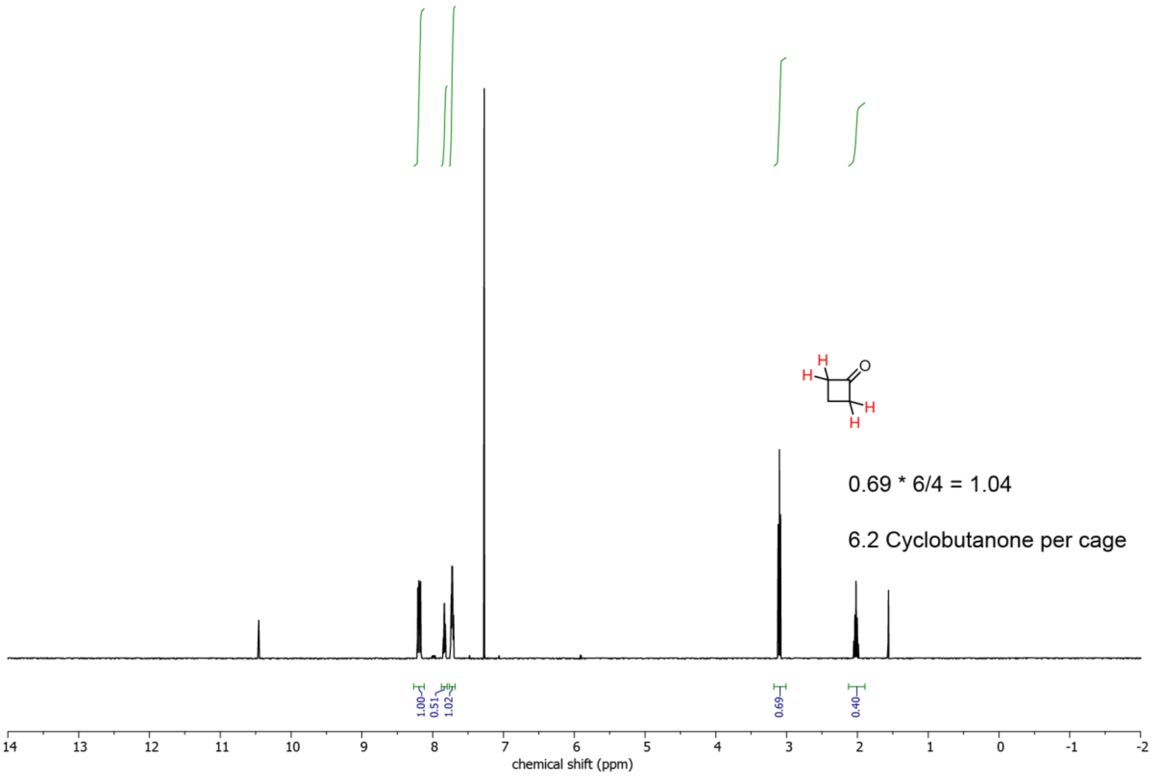

Figure S12.9. ^{1}H NMR spectrum of **1•cyclobutanone**.

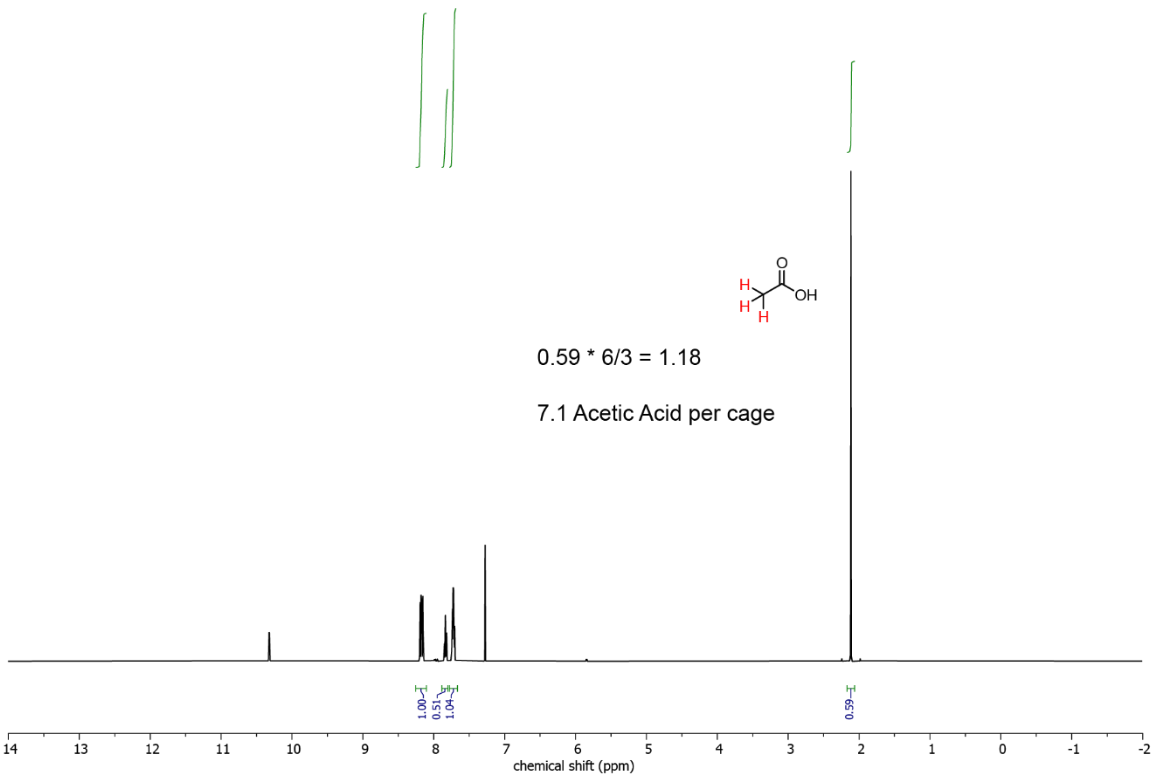

Figure S12.10. ^{1}H NMR spectrum of **1•acetic acid**.

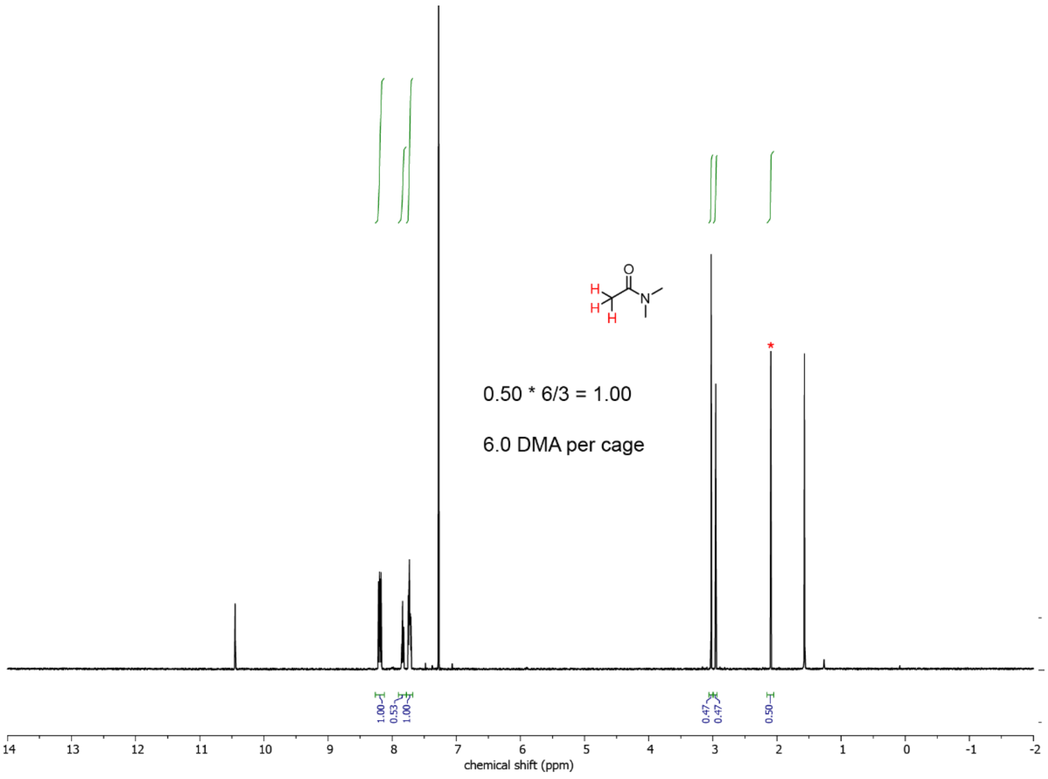

Figure S12.11. ^{1}H NMR spectrum of **1•DMA**.

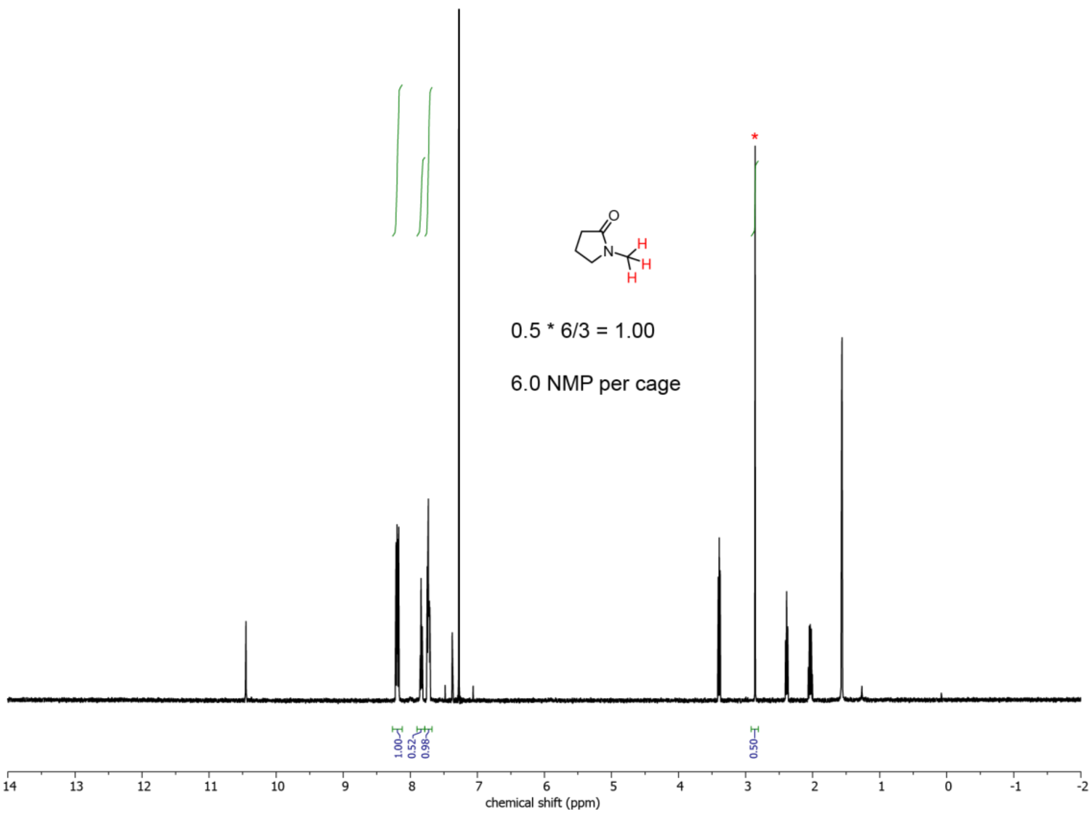

Figure S12.12. ^{1}H NMR spectrum of **1•NMP**.

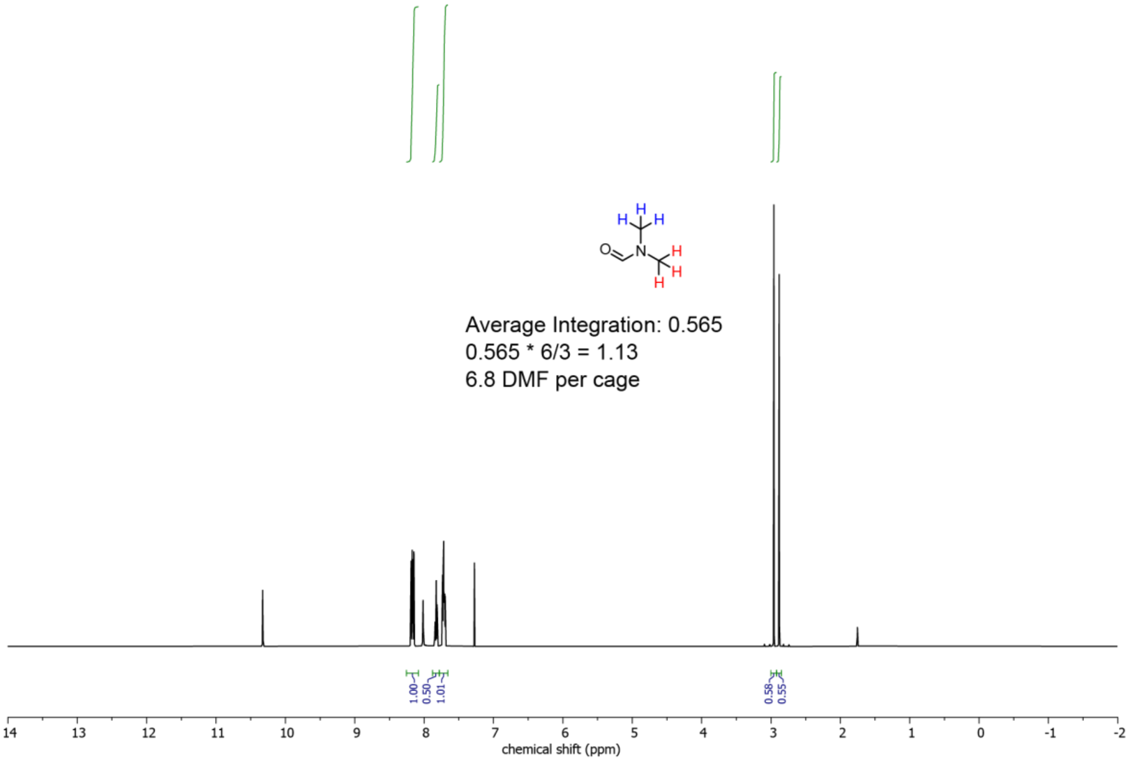

Figure S12.13. ^{1}H NMR spectrum of **1•DMF**.

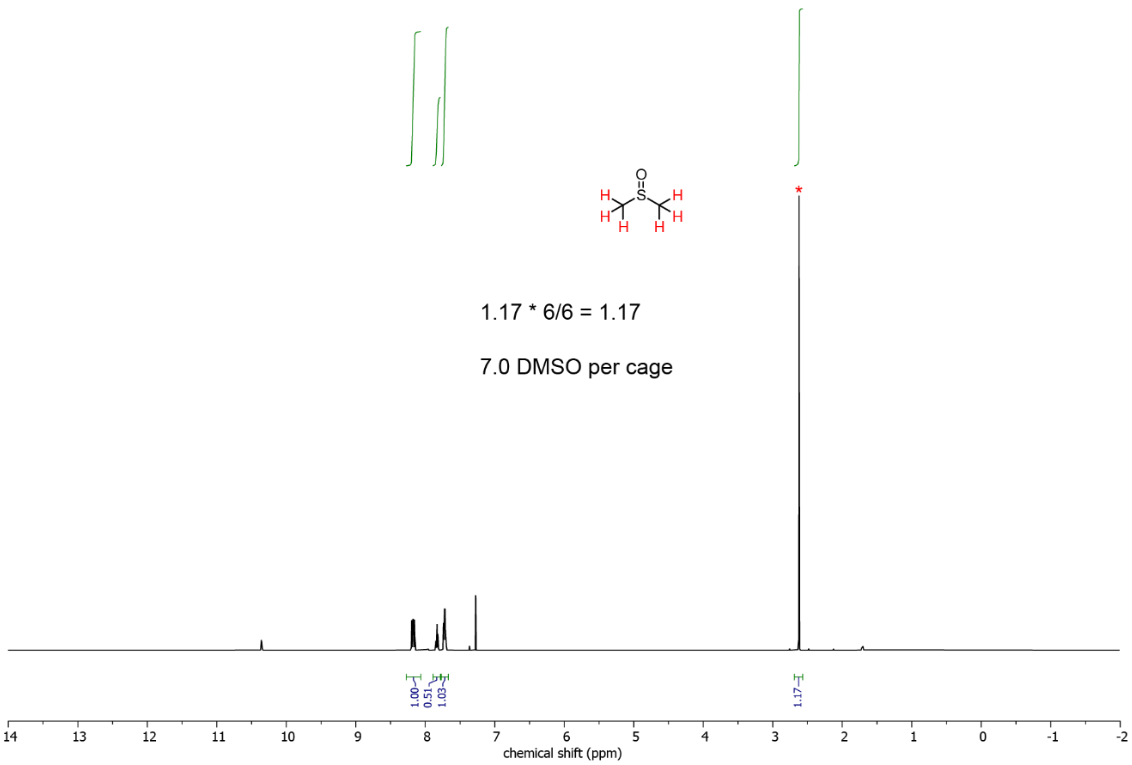

Figure S12.14. ^{1}H NMR spectrum of **1•DMSO**.

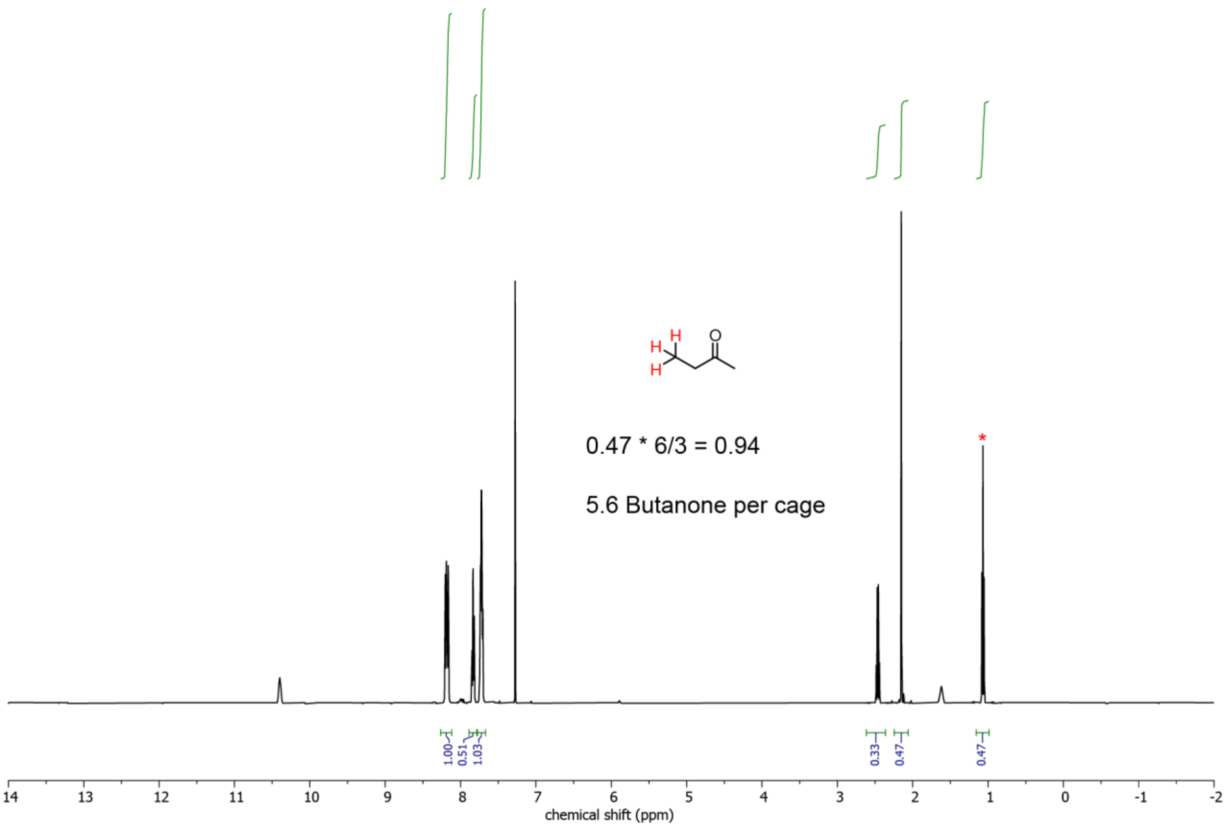

Figure S12.15. ^{1}H NMR spectrum of **1•butanone**.

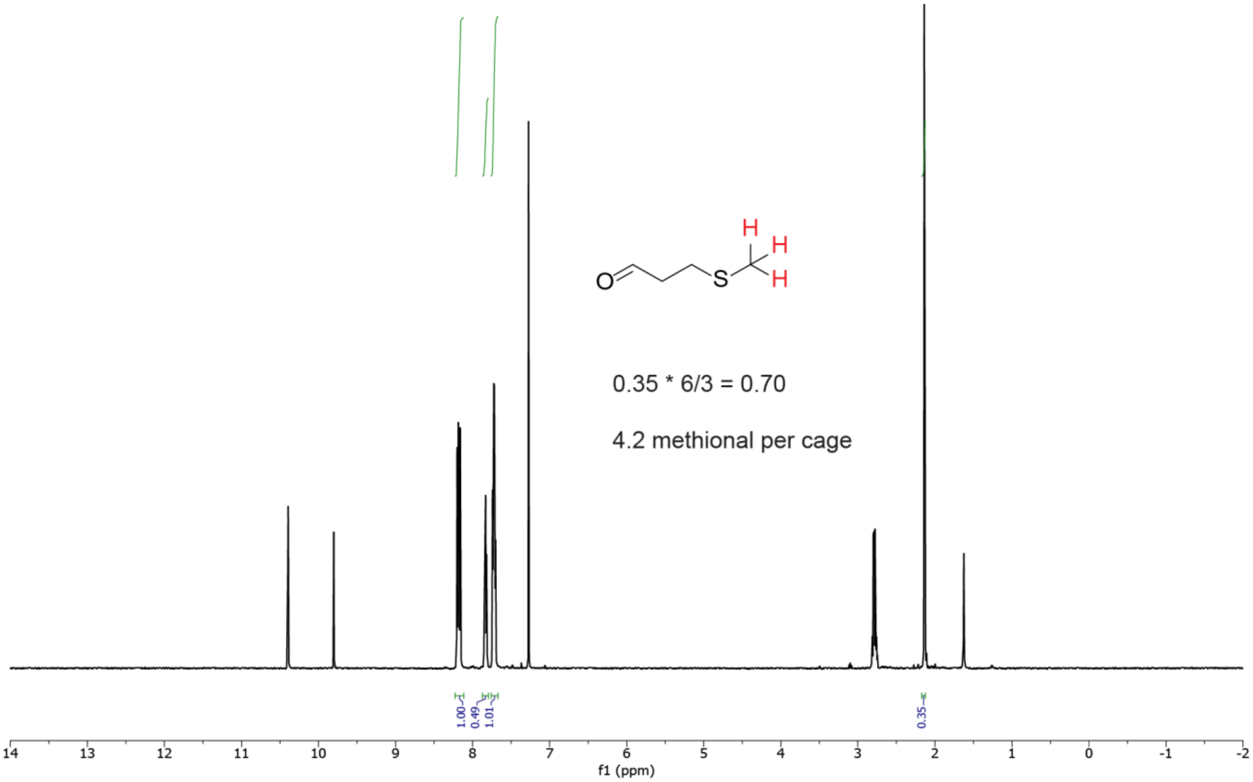

Figure S12.16. ^{1}H NMR spectrum of **1•methional**.

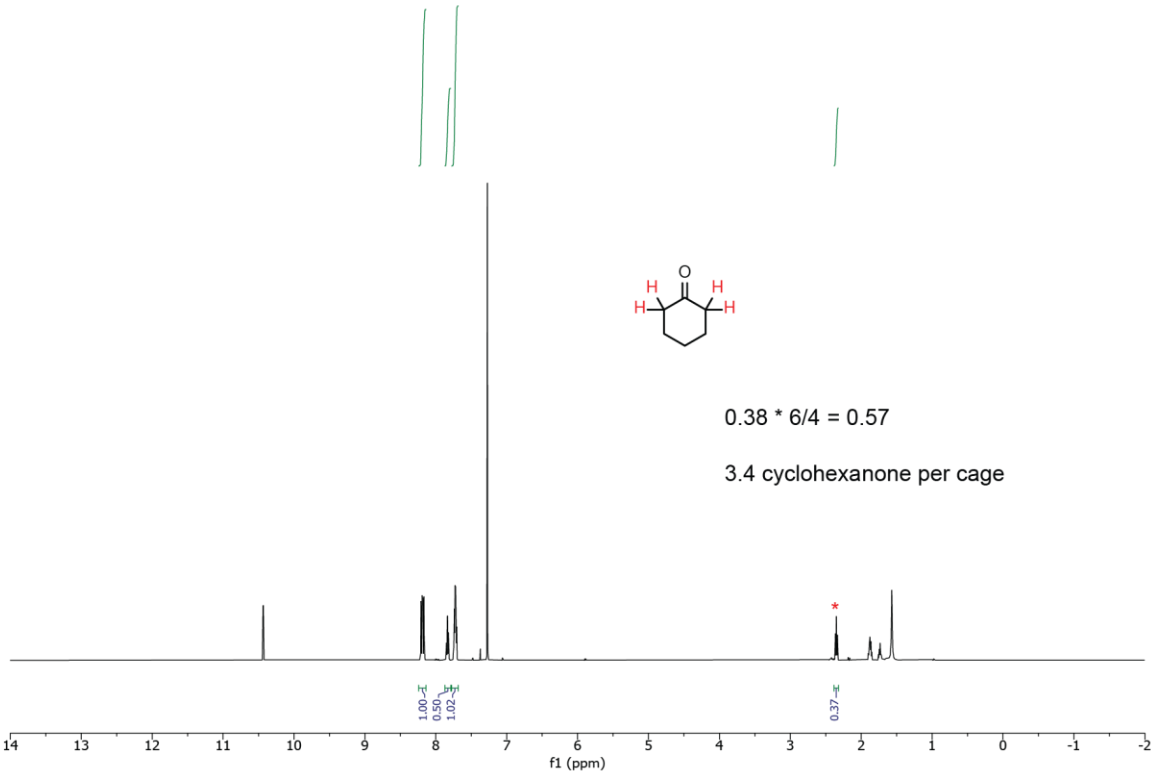

Figure S12.17. ^{1}H NMR spectrum of **1•cyclohexanone**.

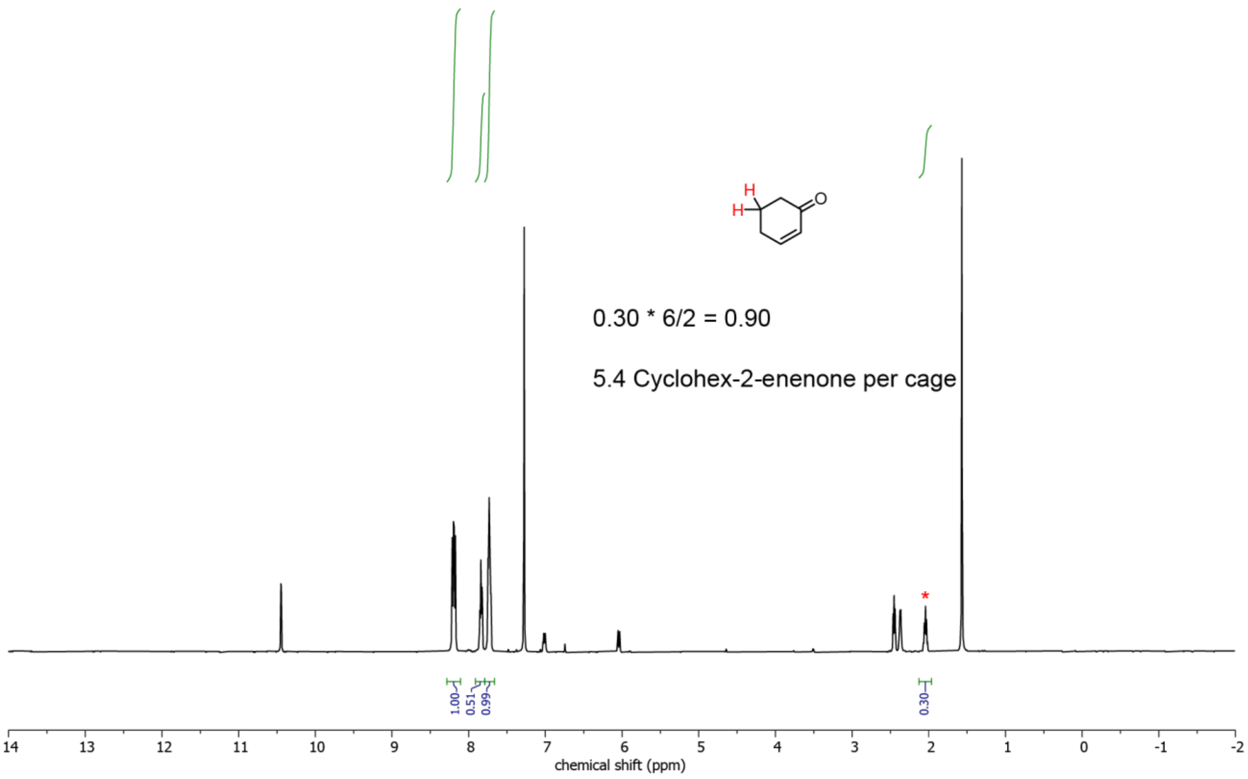

Figure S12.18. ^{1}H NMR spectrum of **1•cyclohex-2-enenone**.

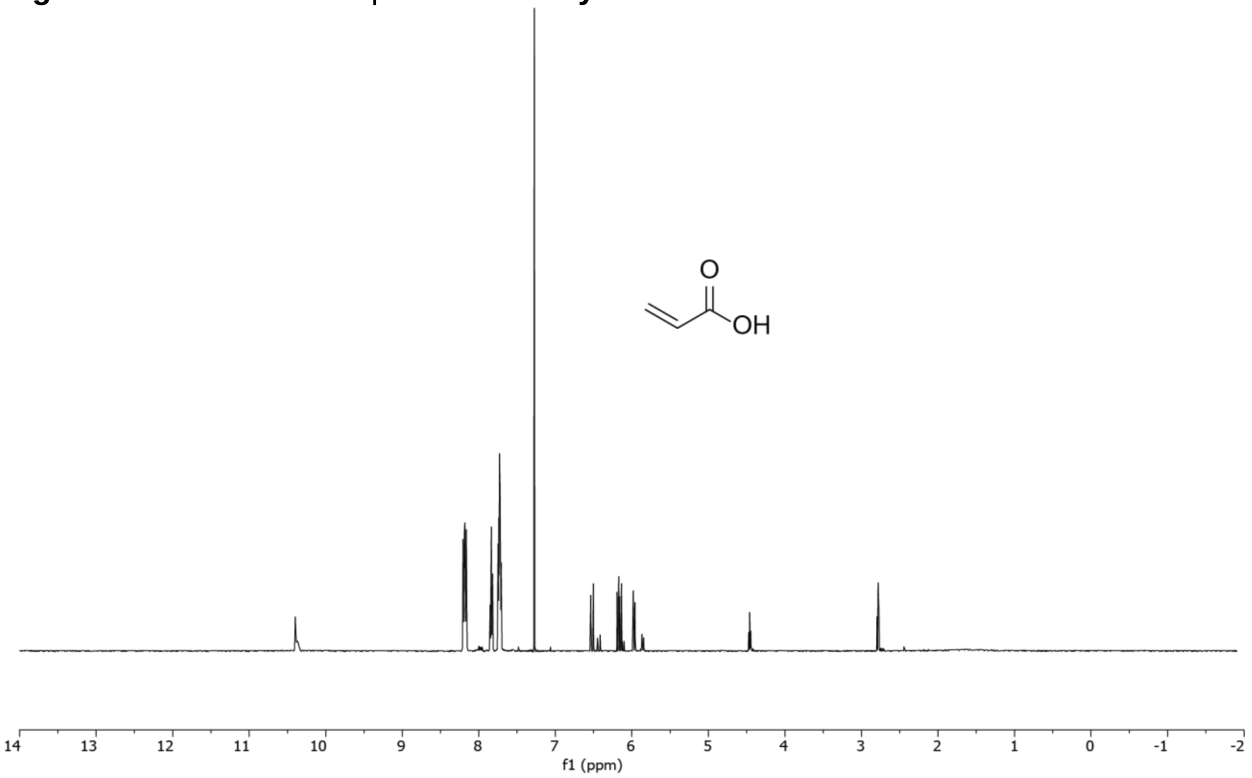

Figure S12.19. ^{1}H NMR spectrum of **1•acrylic acid**.

S13. ^{1}H NMR spectra of crude milling products after Wittig olefination

Conversions are determined by comparing integral values for TPPO with those of the reaction products, as discussed in S11.2. In each spectrum below, the relative signal integrations are denoted with the label "I" above the signal.

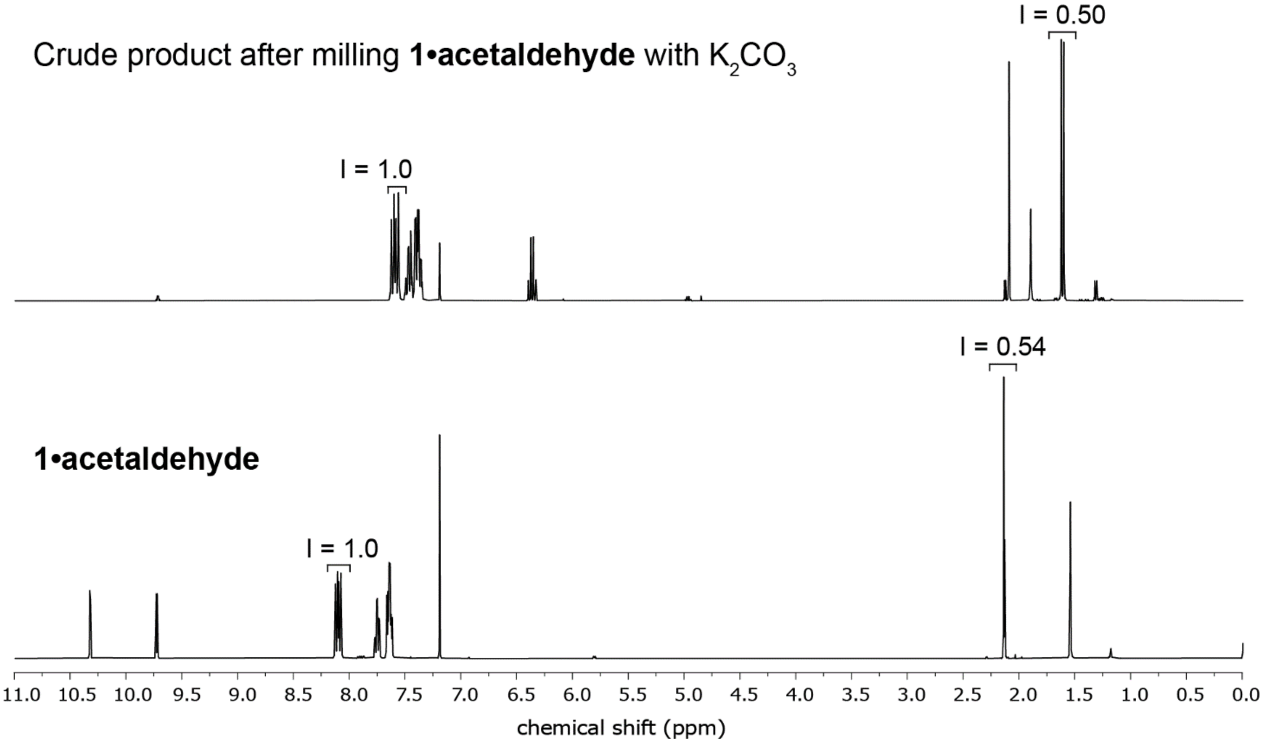

Figure S13.1. ^{1}H NMR spectra of **1•MeCHO** and the crude product of milling **1•MeCHO** with a base.

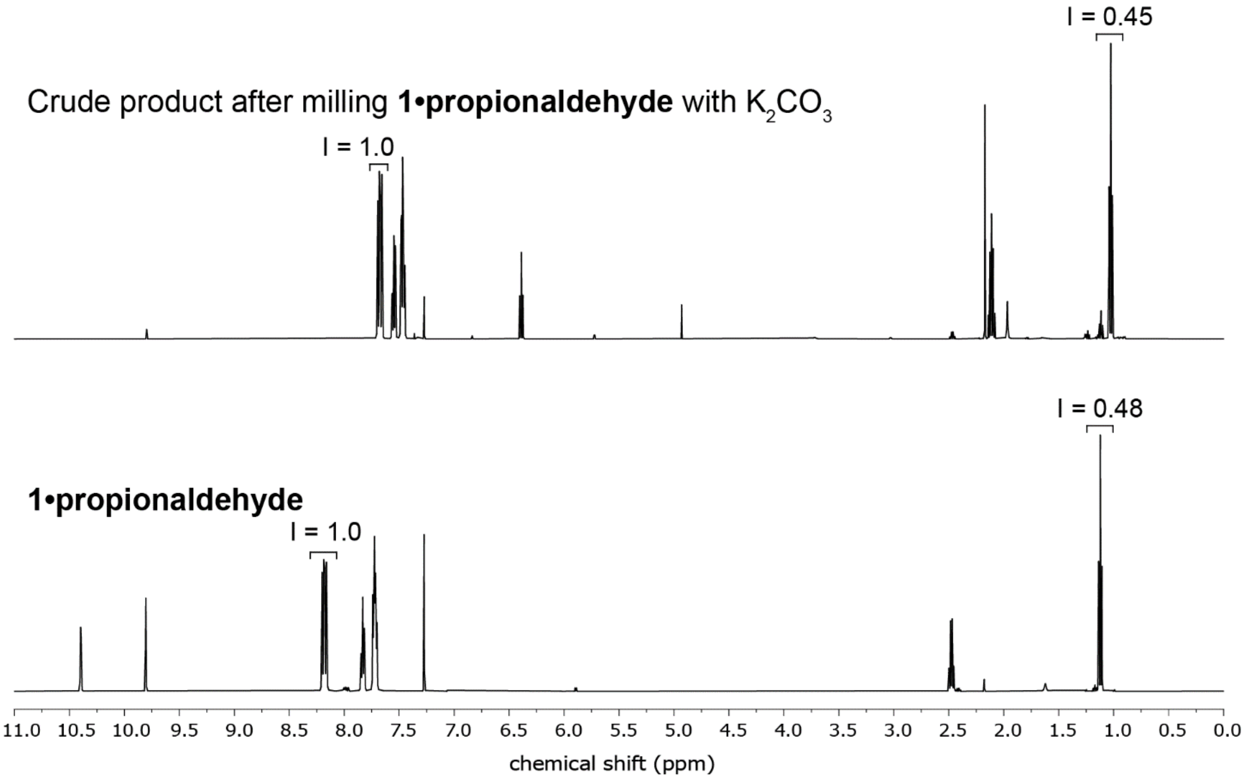

Figure S13.2. ^{1}H NMR spectra of **1•EtCHO** and the crude product of milling **1•EtCHO** with a base.

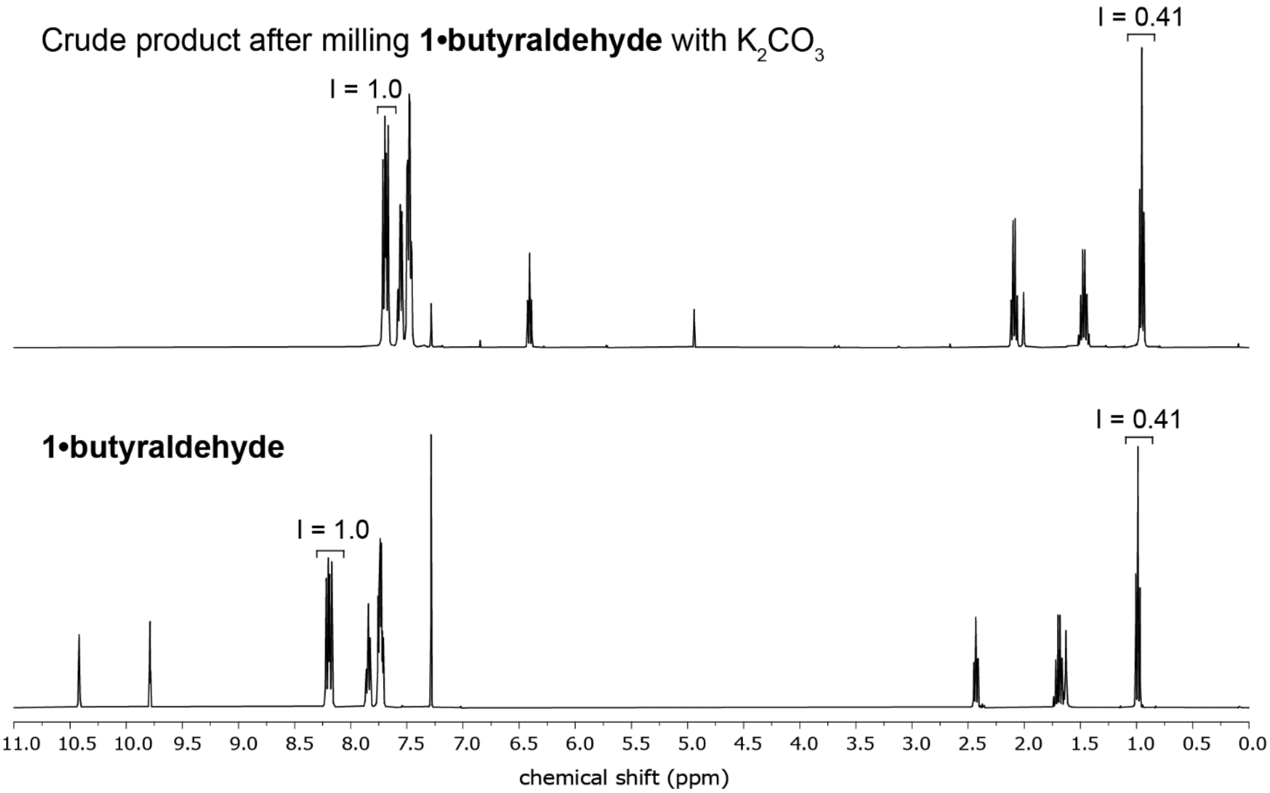

Figure S13.3. ^{1}H NMR spectra of **1•butyraldehyde** and the crude product of milling **1•butyraldehyde** with a base.

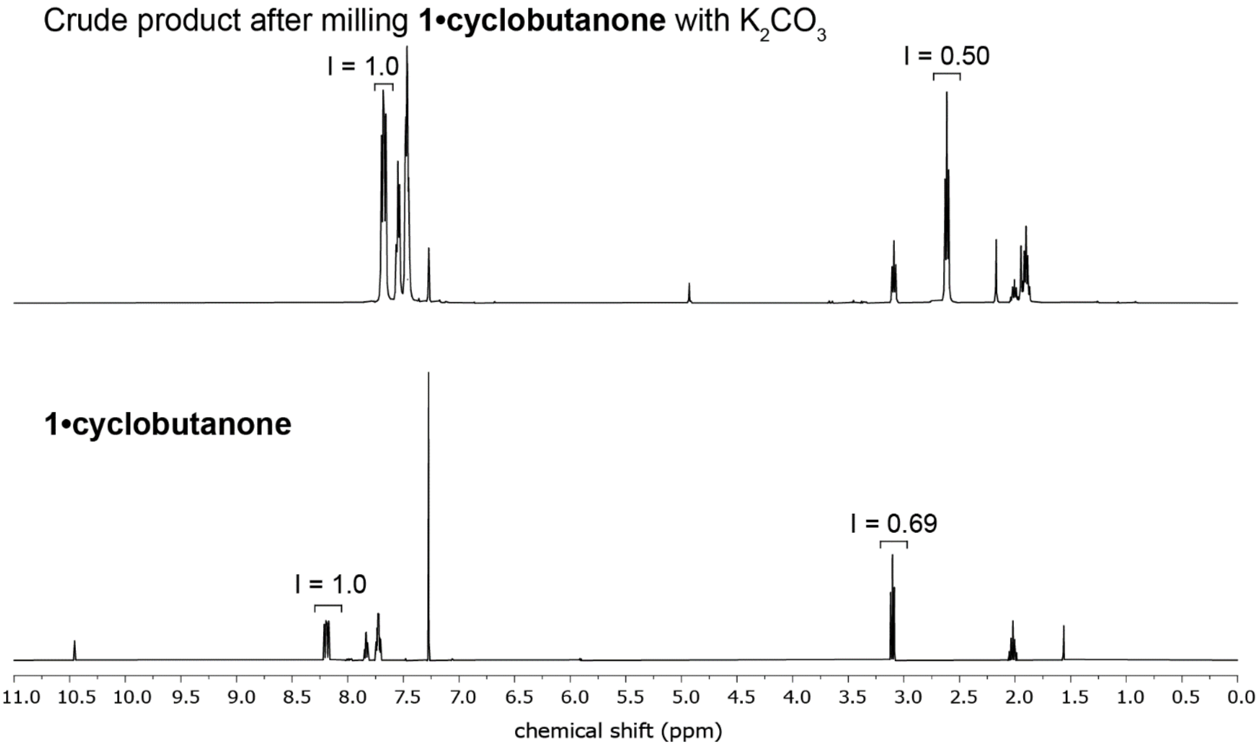

Figure S13.4. ^{1}H NMR spectra of **1•cyclobutanone** and the crude product of milling **1•cyclobutanone** with a base.

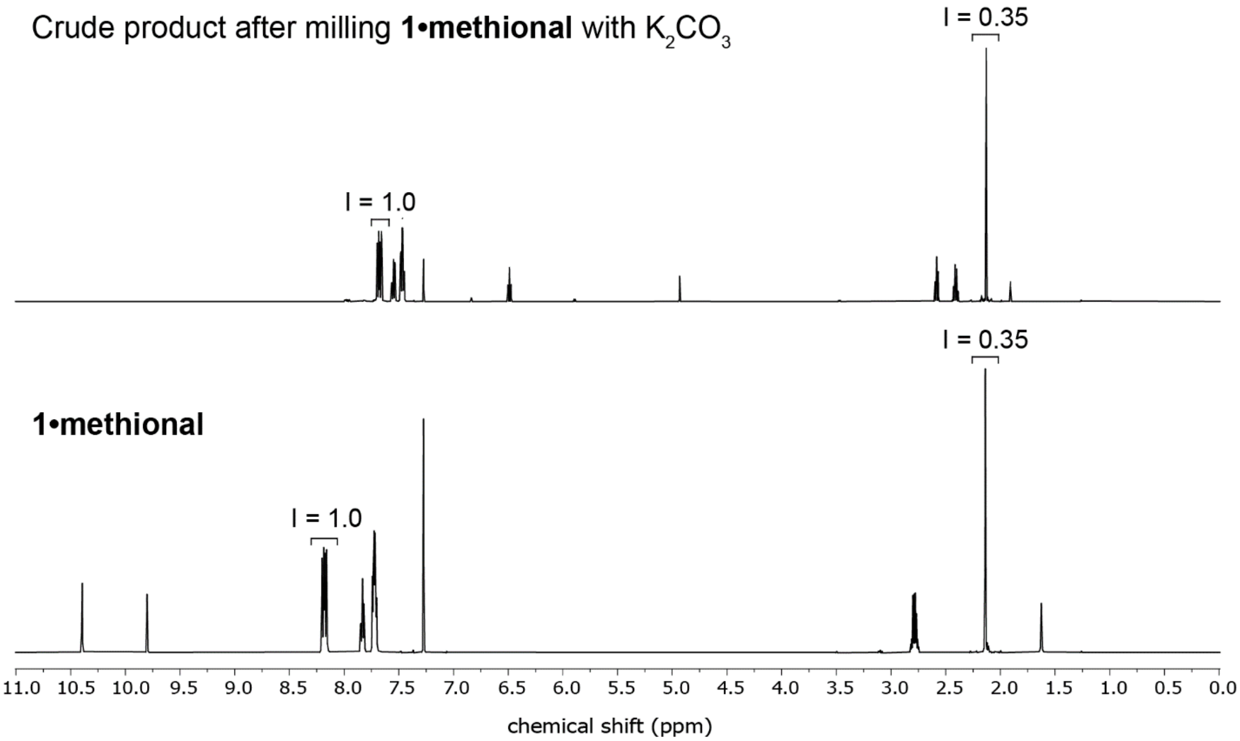

Figure S13.5. ^1H NMR spectra of **1•methional** and the crude product of milling **1•methional** with a base.

S14. ^{1}H and ^{13}C NMR spectra of purified products

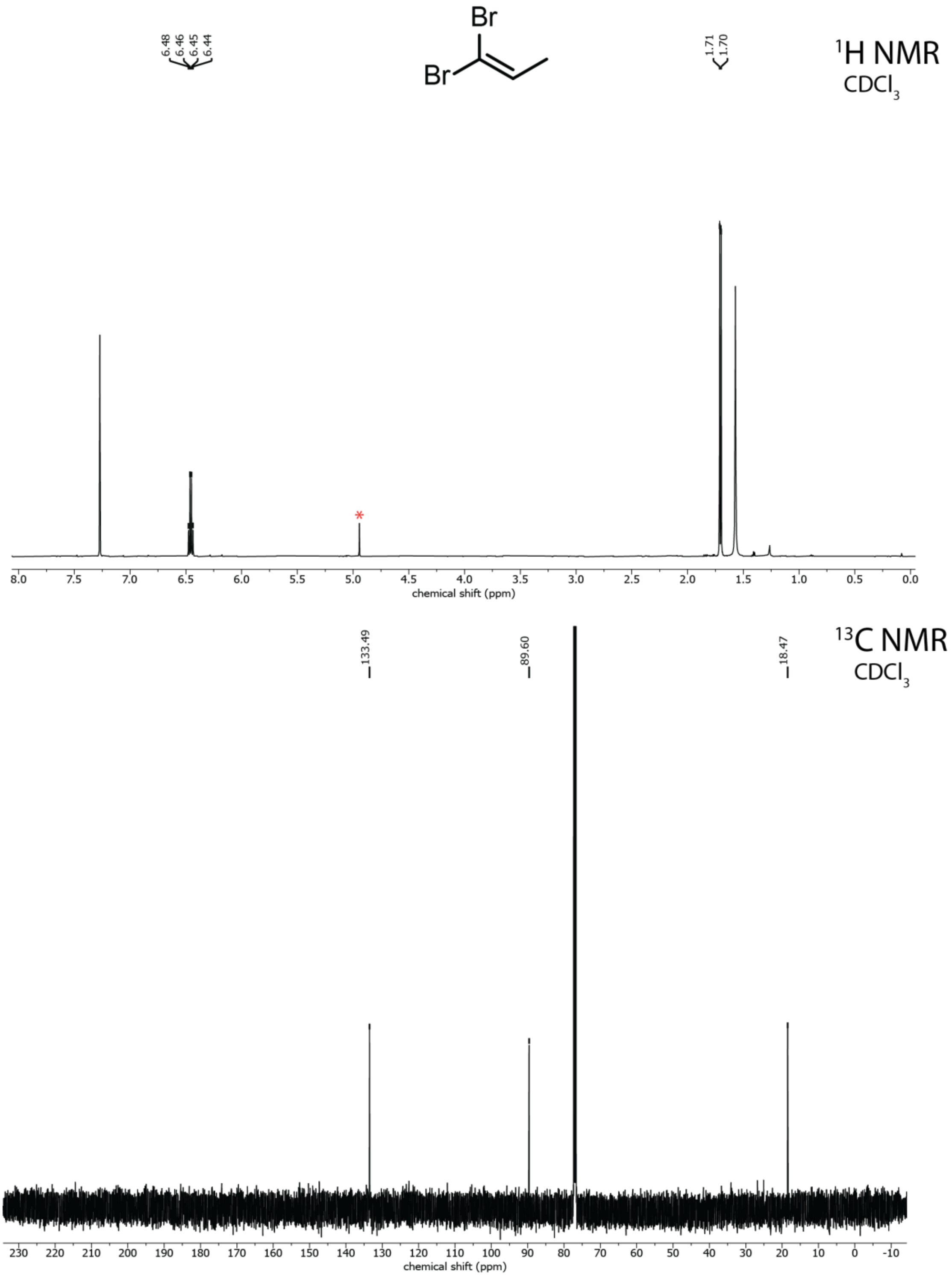

Figure S14.1. ^{1}H and ^{13}C NMR spectra of purified 1,1-dibromopropene produced by mechanochemical Wittig olefination using **1•acetaldehyde**. Signals of the dibromomethane impurity marked with “*”.

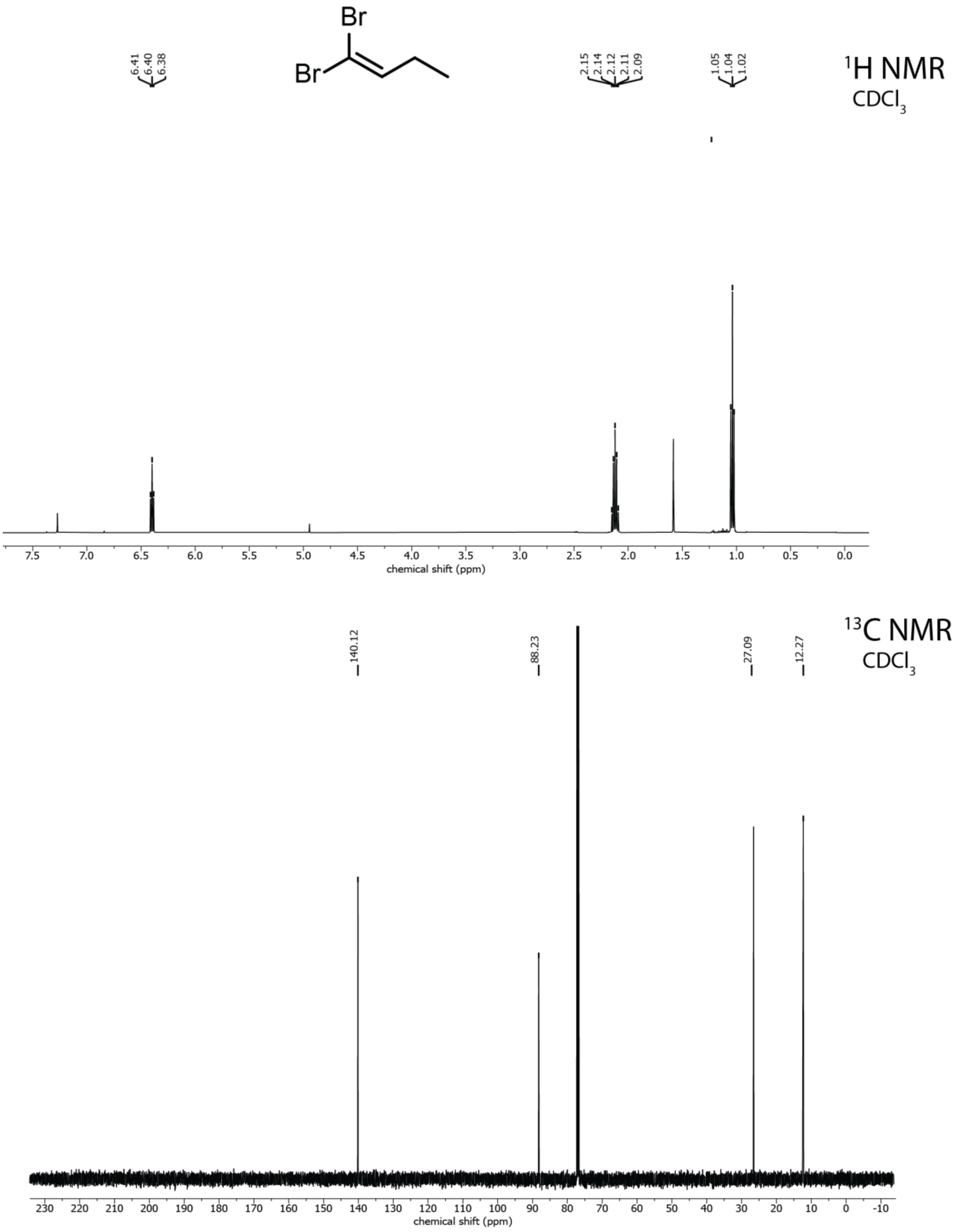

Figure S14.2. ^{1}H and ^{13}C NMR spectra of purified 1,1-dibromobutene produced by mechanochemical Wittig olefination using **1•propionaldehyde**.

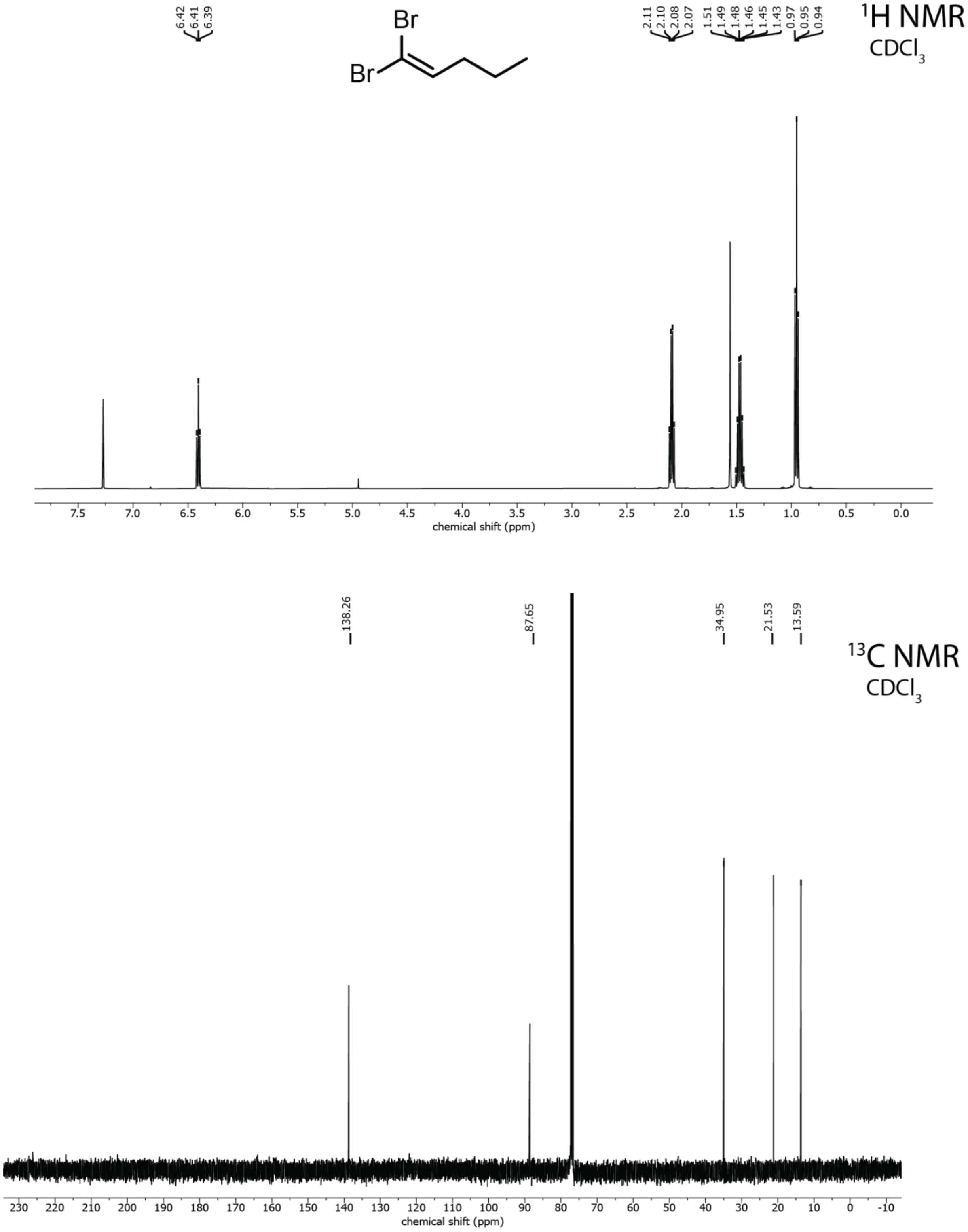

Figure S14.3. ^{1}H and ^{13}C NMR spectra of purified 1,1-dibromopentene produced by mechanochemical Wittig olefination using **1•butyraldehyde**.

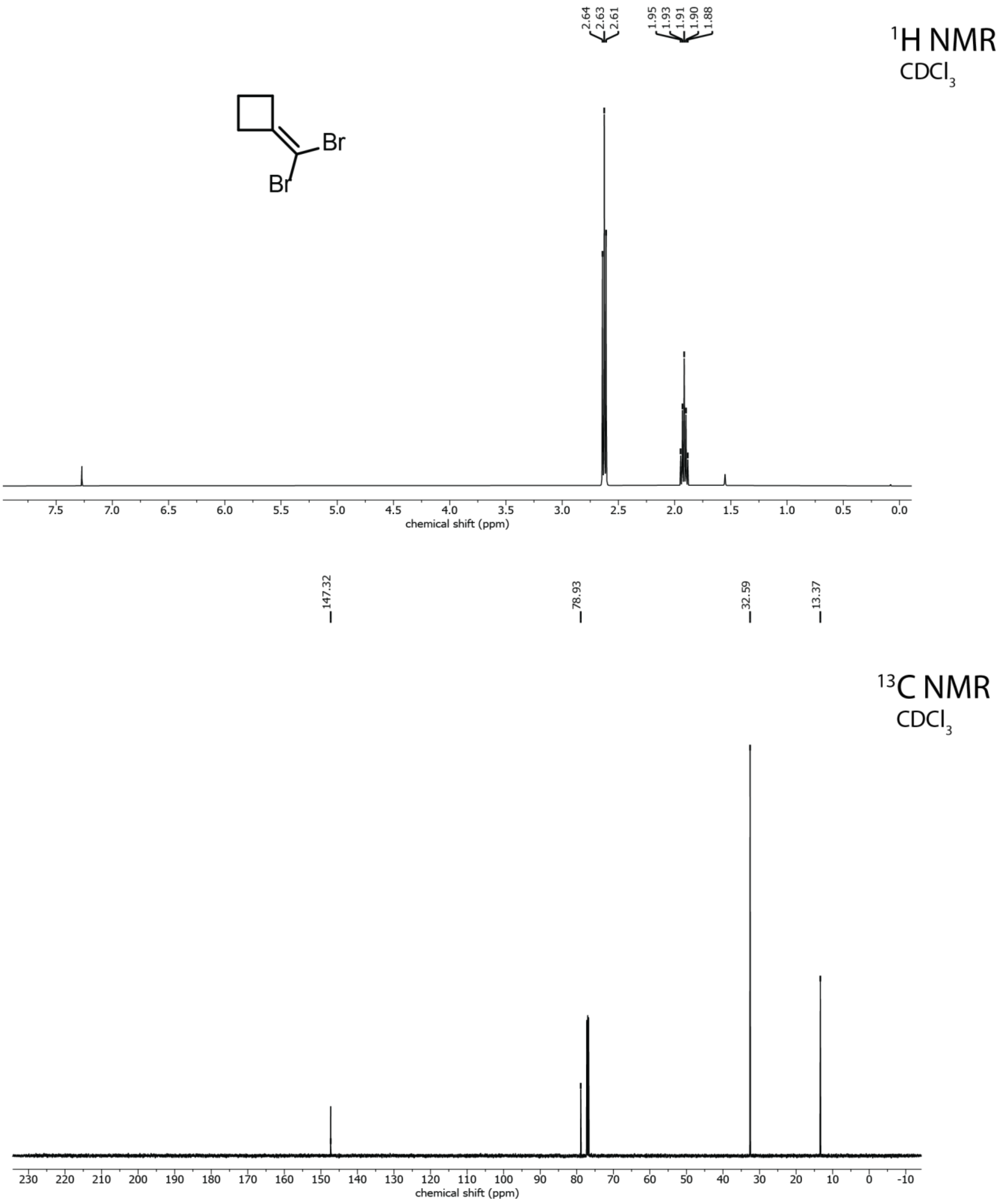

Figure S14.4. ^{1}H and ^{13}C NMR spectra of purified (dibromomethylene)cyclobutane produced by mechanochemical Wittig olefination using **1•cyclobutanone**.

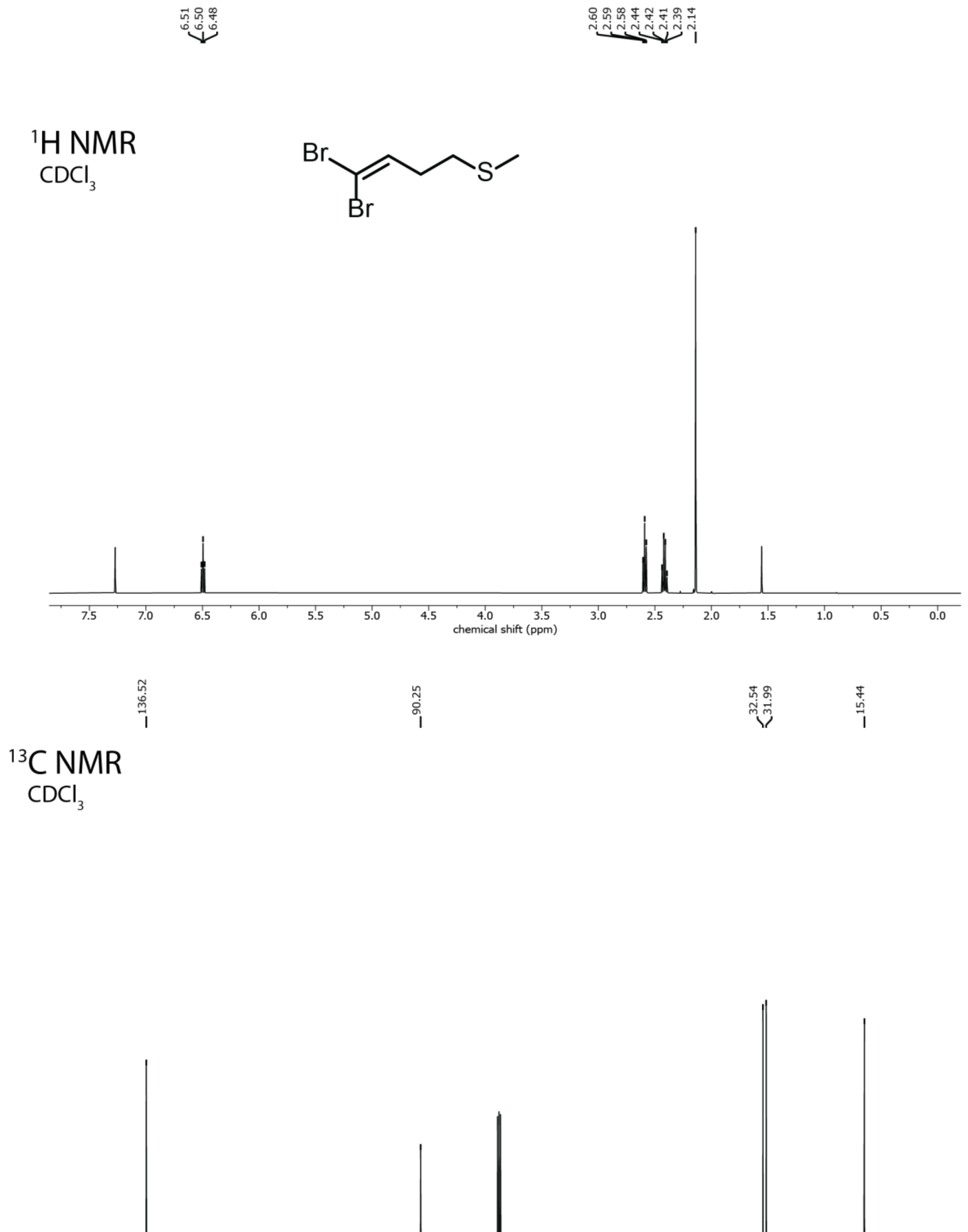

Figure S14.5. ^{1}H and ^{13}C NMR spectra of purified 1,1-dibromo-4-methylthiobutene produced by mechanochemical Wittig olefination using **1•methional**.

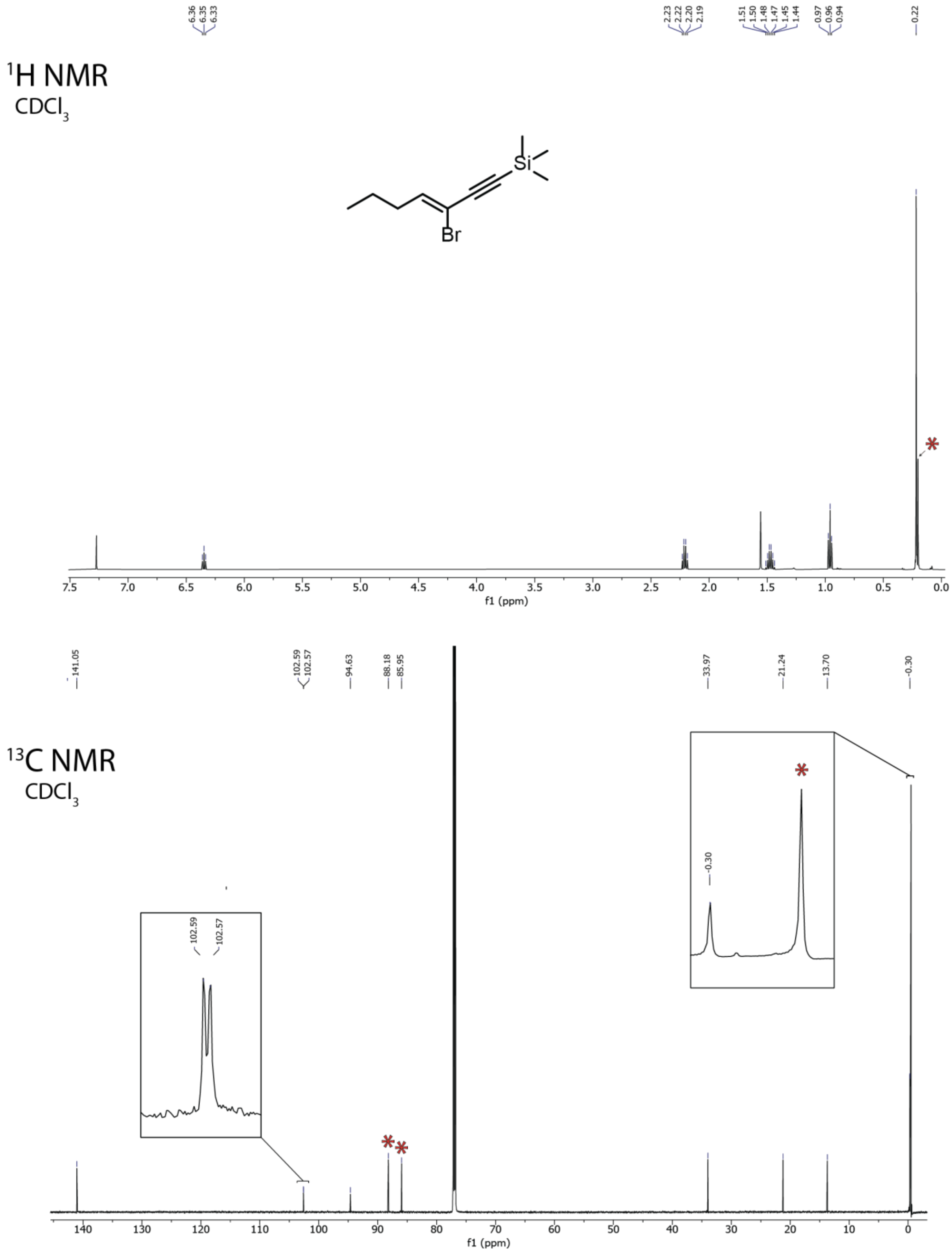

Figure S14.6. ^{1}H and ^{13}C NMR spectra of the purified eneyne Sonogashira product produced by the combined mechanochemical Wittig olefination and Sonogashira coupling using **1•butyraldehyde**. Signals of the (trimethylsiliyl)acetylene homocoupling dimer impurity are marked with “*”.

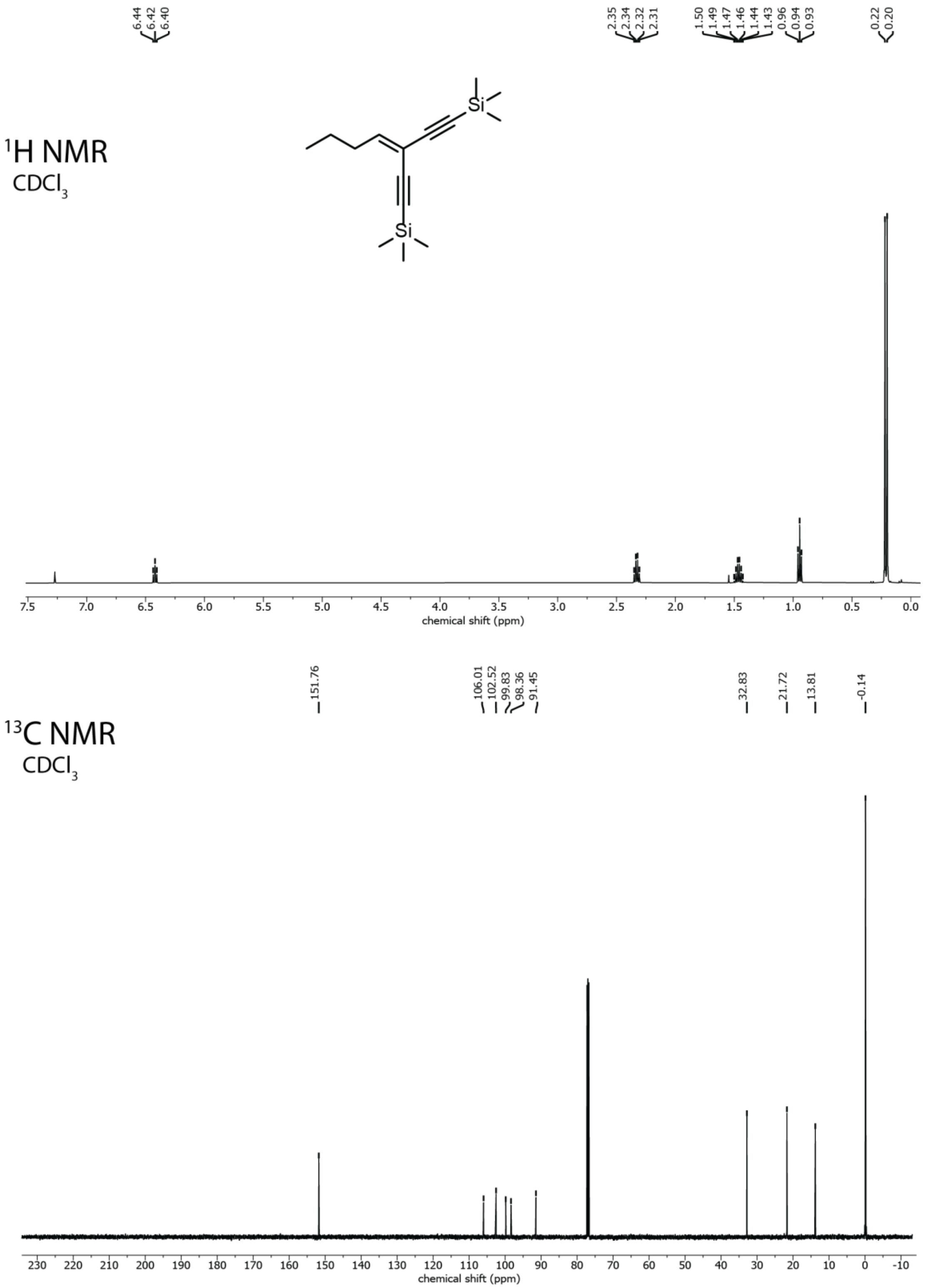

Figure S14.7. ^{1}H and ^{13}C NMR spectra of the purified enediyne Sonogashira product produced by the combined mechanochemical Wittig olefination and Sonogashira coupling using **1•butyraldehyde**.

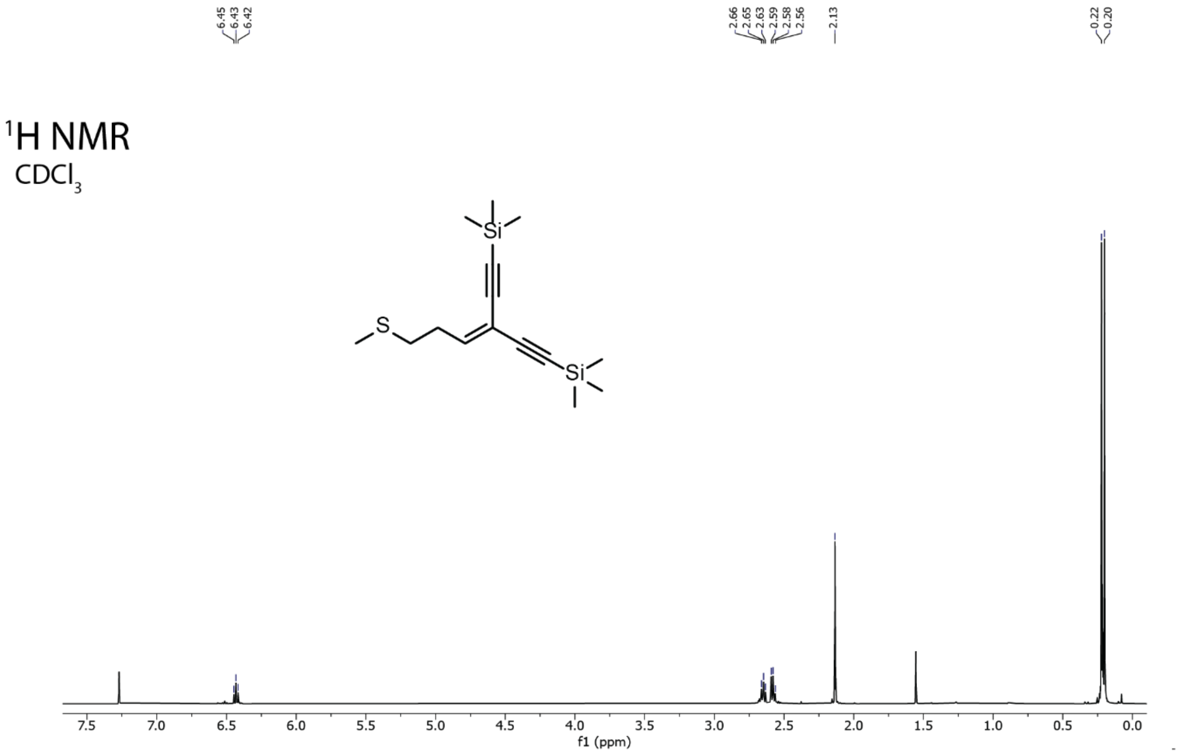

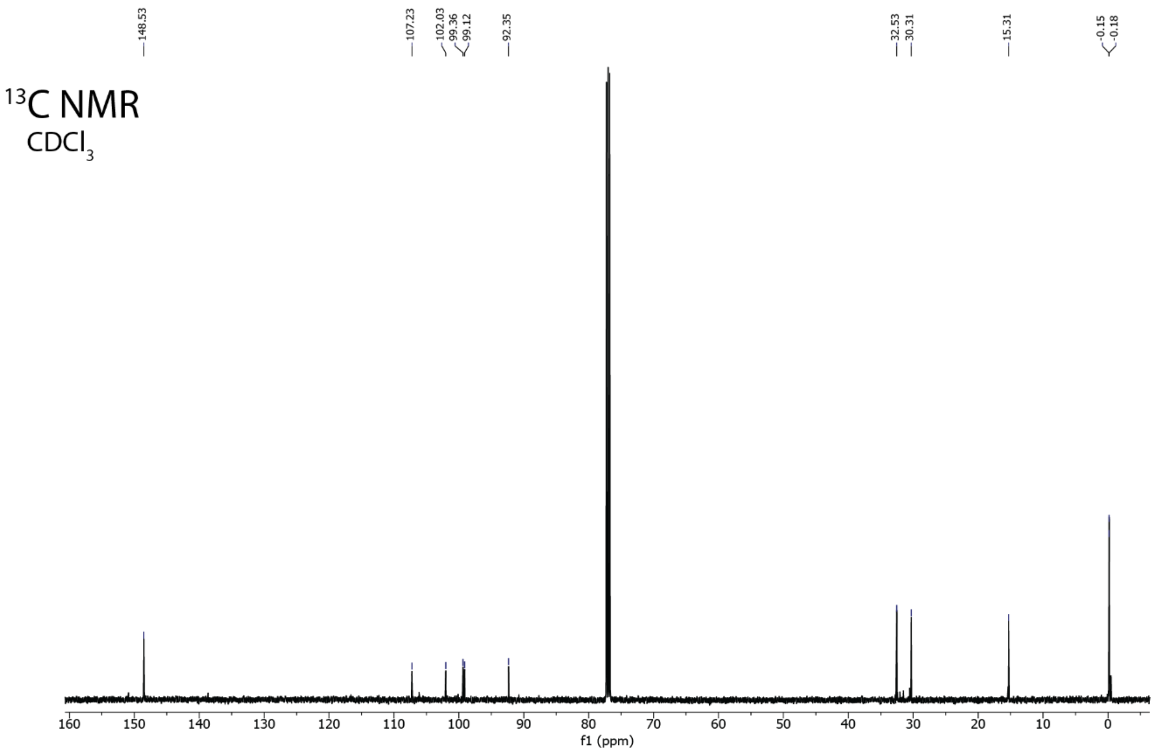

Figure S14.8. ^{1}H and ^{13}C NMR spectra of the purified enediyne Sonogashira product produced by the combined mechanochemical Wittig olefination and Sonogashira coupling using **1•methional**.

S15. References

1. P. Wolkoff, *Can. J. Chem.*, 1975, **53**, 1333-1335.
2. J. Uenishi, K. Matsui and H. Ohmiya, *J. Organomet. Chem.*, 2002, **653**, 141-149.
3. R. Appel and W. Morbach, *Synthesis*, 1977**, 10**, 699-700.
4. M. J. Frisch, *et. al*., Gaussian 16, Revision C.01, Gaussian Inc., Wallingford, CT, 2016.
5. A. D. Becke, *J. Chem. Phys.*, 1993, **98**, 5648-5652;
6. B. P. Pritchard, D. Altarawy, B. Didier, T. D. Gibson and T. L. Windus, *J. Chem. Inf. Model.*, 2019, **59**, 4814-4820.
7. K. Momma and F. Izum, *J. Appl. Cryst.*, 2011, **44**, 1271-1276.
8. K. Wolinski, J. F. Hinton and P. Pulay, *J. Am. Chem. Soc.*, 1990, 112, 8251-8260.
9. W. Deng, J. R. Cheeseman and M. J. Frisch, *J. Chem. Theory Comput.*, 2006, **2**, 1028-1037.
10. G. M. Sheldrick, *Acta Cryst.*, 2015, **A71**, 3-8.
11. G. M. Sheldrick, *Acta Cryst.*, 2015, **C71**, 3-8.
12. A. L. Spek, *Acta Cryst.*, 2015, **C71**, 9-18.
13. G. Ruban and V. Zabel, *Cryst. Struct. Commun.*, 1976, **5**, 671.
14. H. Ott, *Z. Kristallogr. Kristallgeom. Kristallphys. Kristallchem.*, 1926, **63**, 222-230.
15. J. Saurí, J. F. Espinosa and R. Parella, *Angew. Chem. Intl. Ed.*, 2012, **51**, 3919-3922.
